# Single‐nucleus and spatial transcriptomics of paediatric ovary: Molecular insights into the dysregulated signalling pathways underlying premature ovarian insufficiency in classic galactosemia

**DOI:** 10.1002/ctm2.70043

**Published:** 2024-10-23

**Authors:** Raghuveer Kavarthapu, Hong Lou, Thang Pham, Han Do, Mary E. Soliman, Taylor Badger, Ramya Balasubramanian, Victoria Huyhn, Maria De La Luz Sierra, Jacqueline C. Yano Maher, Veronica Gomez‐Lobo

**Affiliations:** ^1^ Division of Pediatric and Adolescent Gynecology Eunice Kennedy Shriver National Institute of Child Health and Human Development Bethesda Maryland USA; ^2^ BioTuring Inc San Diego California USA; ^3^ Division of Pediatric and Adolescent Gynecology Children's National Hospital Washington District of Columbia USA

**Keywords:** classic galactosemia, human ovary, premature ovarian insufficiency, primordial follicles, single‐nucleus RNA sequencing, spatial transcriptomics

## Abstract

**Background:**

Classic galactosemia (CG) is an inborn error of galactose metabolism caused by mutations in the *GALT* gene. Premature ovarian insufficiency (POI) is a later complication that affects 80% of women with CG due to a significant decline in ovarian reserve (primordial follicle pool). The definite mechanisms underlying the early onset of POI in CG patients are not fully understood.

**Methods:**

In this study, we performed single‐nucleus RNA sequencing (snRNA‐seq) and spatial transcriptomics on ovary tissue biopsies from prepubertal girls diagnosed with CG to investigate dynamic changes in gene expression and altered signalling pathways in granulosa cells, oocytes, and stromal cells.

**Results:**

We generated single‐nucleus and spatial transcriptomics atlas of human ovaries from prepubertal girls diagnosed with and without CG. snRNA‐seq profiling of the paediatric ovary revealed a diverse ovarian microenvironment with seven distinct major cell types. Our transcriptomic analysis revealed an increase in the expression of several endoplasmic reticulum stress and oxidative stress associated genes, which can promote apoptosis of granulosa cells in CG. PTEN/PI3K/AKT signalling, which is crucial for primordial follicle activation and survival was dysregulated as supported by upregulated *PTEN* transcripts and a significant reduction in phospho‐AKT levels in the granulosa cells and oocytes. We also found a marked increase in expression of phospho‐H2A.X, LC3A/B and CASP9 in the primordial follicles of CG ovaries suggesting DNA damage, autophagy, and accelerated follicular atresia. Furthermore, we noticed genes participating in extracellular matrix organisation, integrin and gap junction signalling, essential for structural support of the ovarian stroma were profoundly altered.

**Conclusions:**

Our findings provide molecular insights into the dysregulated cellular signalling pathways essential for primordial follicle growth and survival that can explain the etiology of POI in CG patients. This study has implications in the development of future therapeutic interventions to preserve ovarian function and promote female reproductive health.

**Highlights:**

- Created a comprehensive single‐nucleus transcriptomic atlas and spatial landscape of paediatric ovary tissue from prepubertal girls diagnosed with classic galactosemia (CG).
- Our transcriptomic analysis revealed activation of genes associated with ER‐stress signalling, oxidative stress response and ATM signalling/DNA damage response as shown by significant increase in expression of p‐EIF2A, p‐H2A.X and LC3A/B in the primordial follicles of CG ovary.
- PTEN/PI3K/AKT signalling pathways was dysregulated evidenced by a significant reduction in phospho‐AKT expression in the primordial follicles of CG ovary, suggesting impaired follicle activation and survival.

## 1 BACKGROUND

Galactosemia is a condition characterised by a deficiency in enzymes crucial for the Leloir pathway that is required for the metabolism of galactose.^1^ Classic galactosemia (CG) is the most common and severe form of galactosemia, caused by mutations in the *GALT* gene encoding the enzyme galactose‐1‐phosphate uridylyltransferase.^2^, ^3^ It is an autosomal recessive disorder affecting 1:14,000–1:80, 000 individuals globally.^4^ Accumulation of elevated levels of metabolites such as galactose‐1‐phosphate and galactitol due to deficiency of the GALT enzyme has been observed in these patients and may contribute to pathophysiology of CG.^5^ Early signs in infants include failure to thrive, liver damage, kidney failure, jaundice, *E. coli* sepsis, and neonatal death.^2^, ^3^ Hence, newborns are routinely screened for CG by measuring GALT enzyme activity and elevated erythrocyte galactose‐1‐phosphate levels which allows timely diagnosis early in life.^5^ Restricting dietary intake of galactose has been found to effectively alleviate initial neonatal symptoms, but long‐term chronic issues persist with varying severity and include developmental delay, abnormalities in motor function, delays in speech and language acquisition, and early‐onset premature ovarian insufficiency (POI).^2^, ^6^

POI, also known as premature/primary ovarian insufficiency, is a condition characterised by the spontaneous disruption of normal ovarian function, resulting in hypergonadotropic hypogonadism in individuals under the age of 40.^6^, ^7^ POI is one of the most common complications impacting 80%–90% of female patients who adhere to a galactose‐restricted diet.^6^ The way POI manifests in patients with CG in a clinical setting can involve delayed or entirely absent puberty, oligomenorrhea, primary or secondary amenorrhea, or infertility.^6^ Women with CG often show elevated levels of follicle stimulating hormone, lower anti‐mullerian hormone levels, hypoestrogenism and/or normal or increased levels of luteinising hormone.^7^ At birth, the human ovary has a reserve of 1–2 million primordial follicles, comprising an oocyte surrounded by granulosa cells.^8^ Primordial follicles undergo the process of folliculogenesis to reach maturation and release an oocyte/egg through ovulation. Many of these follicles, even in healthy ovaries, undergo atresia rather than ovulation.^9^ The progression of primordial follicles into ovulatory follicles is a continual process throughout a woman's reproductive lifespan, with the number of primordial follicles serving as the ovarian reserve.^10^ Although little is known about the onset of POI in CG, studies have shown that young women with this disorder may have typical ovarian morphology and a normal number of follicles from birth to 5 years of age but show decreased follicles by early puberty.^11^, ^12^ As age progresses, female CG patients with POI exhibit an accelerated loss of follicles with diminished ovarian function, leading to long‐term complications related to hypoestrogenism and infertility.^13^ Our recent clinical data also indicated a significant reduction in mean follicle density and a decline in the primordial follicle count (ovarian reserve) in prepubertal girls with CG compared to controls.^14^ Interestingly, we observed only primordial and transitional primordial stage follicles with almost no primary follicles in the CG ovaries, suggesting impaired follicle activation and growth.^14^ Anti‐mullerian hormone levels were very low to undetectable in all CG patients even though follicles were present, and there was no correlation with mean follicle density. We observed a negative correlation between galactose‐1‐phosphate levels measured from birth to date and mean follicle density in CG patients, although it was not statistically significant.^14^

In recent years, different animal models, including fruit fly, zebrafish, mouse, and rat^15^, ^16^, ^17^, ^18^ have been developed to partially mimic the biochemical and clinical characteristics of CG. Although *GALT*‐KO mouse model revealed growth restrictions, reduced motor functions, and subfertility in adult females, there was no significant reduction in primordial follicles in younger mice at postnatal days 14 and 30.^17^ The animal models of CG may not accurately replicate the condition of POI observed in human CG cases. Moreover, the definite mechanism underlying primordial follicle loss in humans with CG is not fully elucidated. Therefore, to address this question, we utilised both single‐nucleus RNA sequencing (snRNA‐seq) and spatial transcriptomics (ST) techniques to create the first ever transcriptome profiles of human ovary tissue from prepubertal children with CG, which we compared with ‘controls’ (unaffected prepubertal children who underwent ovary removal prior to gonadotoxic therapy for conditions such as cancer). For frozen ovarian tissue samples, snRNA‐seq is a promising method to understand dynamic changes in gene expression patterns in specific cell types such as granulosa cells or oocytes at single cell level. This unique approach to explore the cellular diversity and molecular complexity of ovarian follicles at single cell level will provide an insight into the mechanisms underlying biological processes leading to diminished ovarian reserve in girls with CG. We also performed ST on ovarian tissue sections complementary to the snRNA‐seq method to corroborate our results. ST using the 10x Genomics Visium platform provided an intact two‐dimensional visual landscape of transcripts over an entire ovary section for all our samples. In this study, we successfully identified seven distinct cell types inside the ovarian stroma and characterised four different subpopulations of stromal cells. Our snRNA‐seq and ST results provide insights into the dysregulated cellular stress signalling pathways and PTEN/PI3K/AKT signalling, which can explain the etiology of early POI in prepubertal girls with CG. Finally, our findings are valuable in developing effective future therapeutic strategies to preserve ovarian function to some extent in CG patients and prolong female reproductive health.

## 2 METHODS

### 2.1 Patients and sample collection

This study was conducted in accordance with the Institutional Review Board of Eunice Kennedy Shriver National Institute of Child Health and Human Development (NICHD; protocol numbers IRB000106, IRB00715) at National Institutes of Health and at Children's National Hospital in Washington, DC (IRB protocol numbers Pro00010699, Pro00016433). All patients provided written informed consent. We collected ovarian biopsies from five prepubertal females (age group 5–10 years) diagnosed with CG (CG1‐CG5; Table 1) who underwent laparoscopic unilateral oophorectomy for fertility preservation at NICHD, National Institutes of Health, Bethesda, USA. For comparison, we used ovarian biopsies collected from six prepubertal female children (C1‐C6; Table 1) in a similar age group who underwent the same procedure prior to gonadotoxic therapy for solid tumours or immunodeficiencies prior to bone marrow transplant through a similar protocol at Children's National hospital in Washington, DC. The invasive nature of collecting ovarian tissue precludes the ability to obtain tissue from healthy controls due to ethical considerations. Thus, children with solid tumours or immunodeficiencies which require gonadotoxic therapy were deemed the best controls. All control ovary samples were without histopathological abnormality confirmed by the pathologist. The ovarian tissue collected for research purposes were either flash frozen for snRNA sequencing or fixed in 4% paraformaldehyde for histological evaluation, ST, and immunohistochemistry. Patient demographics showing age, diagnosis, primordial follicle count, and mean follicle density are described in Table 1.

**TABLE 1:** Demographics of 11 patients included in this study for snRNA‐seq and spatial transcriptomics analysis.

Patient #	Diagnosis	Age (year)	Genotype	Primordial follicle count	Mean follicle density (follicles/mm^3^)	SnRNA‐seq	Visium ST	IHC
CG1	Classic galactosemia	10	Q188R/Q188R	30	5.7	Yes	No	Yes
CG2	Classic galactosemia	10	p.L240fs/N97del	86	35.0	Yes	No	Yes
CG3	Classic galactosemia	5	Q188R/Q188R	20	7.9	Yes	Yes	Yes
CG4	Classic galactosemia	7	R148W/R231C	40	6.0	Yes	Yes	Yes
CG5	Classic galactosemia	7	Q188R/Q188R	98	35.4	Yes	Yes	Yes
	**Controls**							
C1	Ewing sarcoma	6	N/A	150	18.2	No	Yes	Yes
C2	DOCK8 deficiency	9	N/A	784	44.5	No	Yes	Yes
C3	Ewing sarcoma	5	N/A	66	16.5	Yes	No	Yes
C4	Rhabdomyosarcoma	7	N/A	191	14.4	Yes	No	Yes
C5	Medulloblastoma	6	N/A	131	12.4	Yes	Yes	No
C6	Medulloblastoma	6	N/A	223	24.7	Yes	No	No

### 2.2 Preparation of single nucleus suspensions from ovary tissue

Nuclei were isolated from flash‐frozen ovarian tissues from five CG and four control patients using a modified method from the 10x Genomics Chromium Nuclei Isolation kit (dx.doi.org/10.17504/protocols.io.6qpvr4jb2gmk/v1). The frozen ovarian biopsies containing both cortex and medulla were placed in eppendorf microtubes containing detergent lysate buffer, minced with scissors, mechanically grinded with a pestle, and incubated on ice for 10 min. The homogenates were collected after repeated pipetting and filtrated through 70 µm filters. The nuclei were purified by washing in nuclei suspension buffer followed by sucrose density gradient centrifugation. The nuclei number and quality were determined using a CellDrop cell counter according to the manufacturer's instructions (DeNovix).

### 2.3 SnRNA‐seq library preparation and sequencing

snRNA‐seq libraries from nuclei isolated from ovarian tissue were constructed using the 10x Genomics Chromium Next GEM Single Cell 3ʹ Kit v3.1 (10x Genomics, PN‐1000268) according to the manufacturer's instructions. In brief, freshly prepared 8000 nuclei were loaded onto a Chromium microfluidic chip, which is used to generate single‐cell gel bead emulsions by the 10x Genomics Chromium controller. Later cDNA amplification was performed, and libraries were prepared as per the manufacturer's protocol. The libraries were quantified, and quality was assessed using a Bioanalyzer (Agilent). Libraries were pooled and sequenced on a S2 flow cell (paired‐end 100 bp) using an Illumina NovaSeq 6000 platform.

### 2.4 SnRNA‐seq data processing and downstream analysis

The Cell Ranger software from 10x Genomics was used to process the raw data. The BCL files generated by Illumina sequencing were demultiplexed using the Cell Ranger v3.1 mkfastq default pipeline to obtain FASTQ files for each sample. Alignment of reads (human reference genome hg38), filtering, barcode and UMI counting were performed to obtain a gene barcode matrix file (gene counts per nucleus) using the Cell Ranger count. For downstream bioinformatic analysis, we used the BioTuring platform (BioTuring Inc.) where filtering of nuclei, clustering and cell type annotation were performed. Initially, the snRNA‐seq count matrix files were loaded in the BioStudio module (BioTuring Inc.) and clustered with the standard Seurat version 4 package. Then, the ambient RNA detection and removal were performed using the SoupX version 1.6.2 with the default parameters. Next, low‐quality nuclei with <400 and >4000 genes/nucleus, and mitochondrial genes >15% were excluded. After filtering, Seurat objects corresponding to individual samples were merged and batch correction was performed using the harmony package. After normalisation and scaling of data, dimensionality reduction was done using the principal component analysis. Cell clustering was performed with Louvain graph clustering method to visualise nuclei clusters in 2D using UMAP. To identify the highly expressed marker genes for cell cluster annotation, the FindAllMarkers function with default Wilcoxon rank sum test was applied. All these steps were performed using BBrowserX module (BioTuring). We then classified different cell types (granulosa cells, oocytes, endothelia cells, muscle cells, epithelial cells, immune cells, and stromal cells) based on specific marker gene expression found in each cluster. Gene ontology (GO) enrichment analysis was performed using the web‐based DAVID functional annotation tool (https://david.ncifcrf.gov/) or the Enrichr tool to obtain significantly enriched biological processes. The R package monocle2 was used for pseudotime analysis to construct cell trajectories for different subpopulations of stromal cells.

### 2.5 ST of human ovary and data analysis

CG and control ovarian sections were processed for complete Visium ST as per the manufacturer's protocol (10x Genomics). Briefly, 5 µm thick formalin‐fixed paraffin‐embedded (FFPE) ovary sections were mounted onto a positively charged slide followed by staining with haematoxylin and eosin. Slides were mounted in 70% glycerol and bright‐field images were taken using microscope. The tissue sections were then transferred immediately onto 6.5 × 6.5 mm or 11 mm × 11 mm capture areas on a Visium gene expression slide using Visium CysAssist (10x Genomics). The tissue sections were then subjected to permeabilisation followed by reverse transcription for cDNA synthesis. Subsequently, the cDNA amplification was performed, and spatial libraries were constructed as per manufacturer's instructions using Visium spatial library construction kit (10x Genomics, PN‐1000187). The libraries were pooled and paired‐end sequencing was performed on S2 flow cell using an Illumina NovaSeq 6000 platform. Spatial barcodes were used to associate the reads back to the tissue section images for Visium gene expression mapping. A total of six capture areas with six tissue sections (3 each for CG and control groups) were sequenced on 10x Visium gene expression slide. Space Ranger v3.0 was used to process raw FASTQ files and histology images of ovary sections to generate feature‐barcode matrices for each ovary section. We processed feature‐barcode files using Seurat package on BioTuring platform for downstream spatial transcriptomic analysis to obtain information about the number of spatial spots, median genes and reads per spot. The data was normalised to account for variance in sequencing depth across and spatially variable genes were identified. To spatially locate ovarian follicles transcriptomic spots, we relied on the cell type specific marker genes for oocytes and granulosa cells that were identified in our snRNA‐seq analysis as reference gene set (*DDX4, ZP2, FIGLA, TUBB8, AMH, HSD17B1* and *FST*). We used AUCell enrichment analysis to identify ST spots corresponding to ovarian follicles highly enriched with active gene set (input reference marker genes) mentioned above. We then performed differential expression analysis on these enriched follicle ST spots between CG and control groups to obtain differentially expressed genes (DEGs).

### 2.6 Functional enrichment and causal network analysis

We first performed differential expression analysis between CG versus control groups on granulosa cells, oocytes, and stromal cells from snRNA‐seq data and ovarian follicle cluster from ST data using the Wilcoxon rank‐sum test to obtain DEGs with fold change ≤ –1 or ≥ 1 and FDR < .05. Functional enrichment analysis was performed on the DEGs using Qiagen Ingenuity Pathway Analysis (IPA) platform to identify significantly enriched canonical signalling pathways, biological functions, upstream transcriptional regulators, and gene networks. Each gene identifier was mapped to its corresponding gene object in the IPA knowledge base. We applied default settings using Fisher's exact test to determine the significantly enriched canonical pathways or biological function with *p* < .05. Upstream regulator and causal network analyses tool by IPA was used to identify potential upstream regulators (transcription factors, kinases, growth factors/receptors) and their corresponding downstream target genes from the DEGs list. Causal network analysis provides mechanistic gene regulatory networks that could explain the observed dynamic changes in gene expression in a canonical signalling pathway(s) or biological function. We also used this causal network analysis tool to understand how different signalling pathways crosstalk among each other through protein‐protein and protein‐gene interactions.

### 2.7 Cell‐cell communication analysis using CellChat

To study and visualise the cell–cell communication changes among different cell types (6 major cell types and 4 stromal cell subpopulations), the R package ‘CellChat’ was used according with default settings. The human ligand‐receptor interaction database CellChatDB was used for the subsequent analysis, and the ‘computeCommunProb’ function from CellChat was employed to calculate the communication probability. We compared the total number of ligand‐receptor interactions and interaction strengths between interacting cell groups and visualised with circle plots and heatmaps.

### 2.8 Immunohistochemistry (IHC) and TUNEL assay

FFPE ovarian sections (5 µm thick) from control and CG patients were deparaffinised and rehydrated in a graded series of ethyl alcohol and distilled water. Antigen retrieval was performed for 20 min with universal epitope recovery buffer (Electron microscopy sciences, PA, USA). IHC was carried out using ImmPRESS Excel Amplified HRP polymer staining kit (Vector Laboratories) as per the manufacturer's protocol. Tissue sections were incubated in BLOXALL reagent for 10 min and washed with PBS for 5 min. Blocking was performed with 2.5% horse serum in PBS for 30 min. After blocking step sections were incubated overnight with 1:250 dilution of respective primary antibodies such as phospho‐AKT (Cell Signaling Technology, cat# 4060), phospho‐EIF2A (Cell Signaling Technology, cat# 3398), phospho‐H2A.X (Cell Signaling Technology, cat# 9718), LC3A/B (Cell Signaling Technology, cat# 12741), cleaved‐CASP3 (Cell Signaling, cat# 9664), and CASP9 (Cell Signaling Technology, cat# 9502). After incubation the sections were washed with PBS and incubated with the amplifier antibody for 15 min. Subsequently, sections were washed with PBS with .1% tween‐20 and incubated with anti‐goat HRP polymer reagent for 30 min. The sections were washed; diaminobenzidine (DAB) reaction was performed to develop the brown colour for visualisation. Sections were counterstained with haematoxylin, washed in 100% ethanol, and mounted using VectaMount medium (Vector Laboratories). To assess ovarian follicular atresia mediated through apoptotic cell‐death, we performed TUNEL staining on ovarian sections according to manufacturer's protocol using HRP‐DAB based TUNEL assay kit (Abcam; cat# ab206386). The immuno‐stained sections were imaged using an EVOS M5000 microscope (Thermo Scientific). The mean intensity of DAB immunosignals in individual follicles were quantitated for CG and control ovary sections (*n* = 3 patients) using QuPath bioimage analysis software (https://qupath.readthedocs.io/en/0.5/).

### 2.9 Mouse ovary culture and D‐galactose treatments

Ovaries were collected from 5‐ to 6‐week albino mice in growth medium containing αMEM (Invitrogen, CA) supplemented with 5% FBS, 1% Insulin‐Transferrin‐Selenium (Sigma‐Millipore, MA), and penicillin‐streptomycin. Each ovary was placed on the top of cell culture inserts (.4 µm) floating on growth medium for 1 day in a CO_2_ incubator at 37°C. Treatments were carried out by adding 5 and 10 mM concentration of D‐galactose to the culture medium for 48 h. After treatment the ovaries were fixed in modified Davidson's fixative and processed to obtain FFPE ovarian sections to perform immunostaining to assess the expression of apoptotic cell‐death marker cleaved‐CASP3 and DNA damage marker phospho‐H2A.X.

### 2.10 Statistical analysis

Statistical analysis was conducted using GraphPad Prism version 9. Data in bar plots are shown as the mean ± SEM obtained from 3 patient samples. Unpaired two‐tailed *t*‐test was used to determine the significant differences between CG and control groups, and *p* value < .05 was considered statistically significant.

## 3 RESULTS

### 3.1 Single‐nucleus transcriptome profiling of human ovary revealed a diverse ovarian microenvironment

**FIGURE 1:**
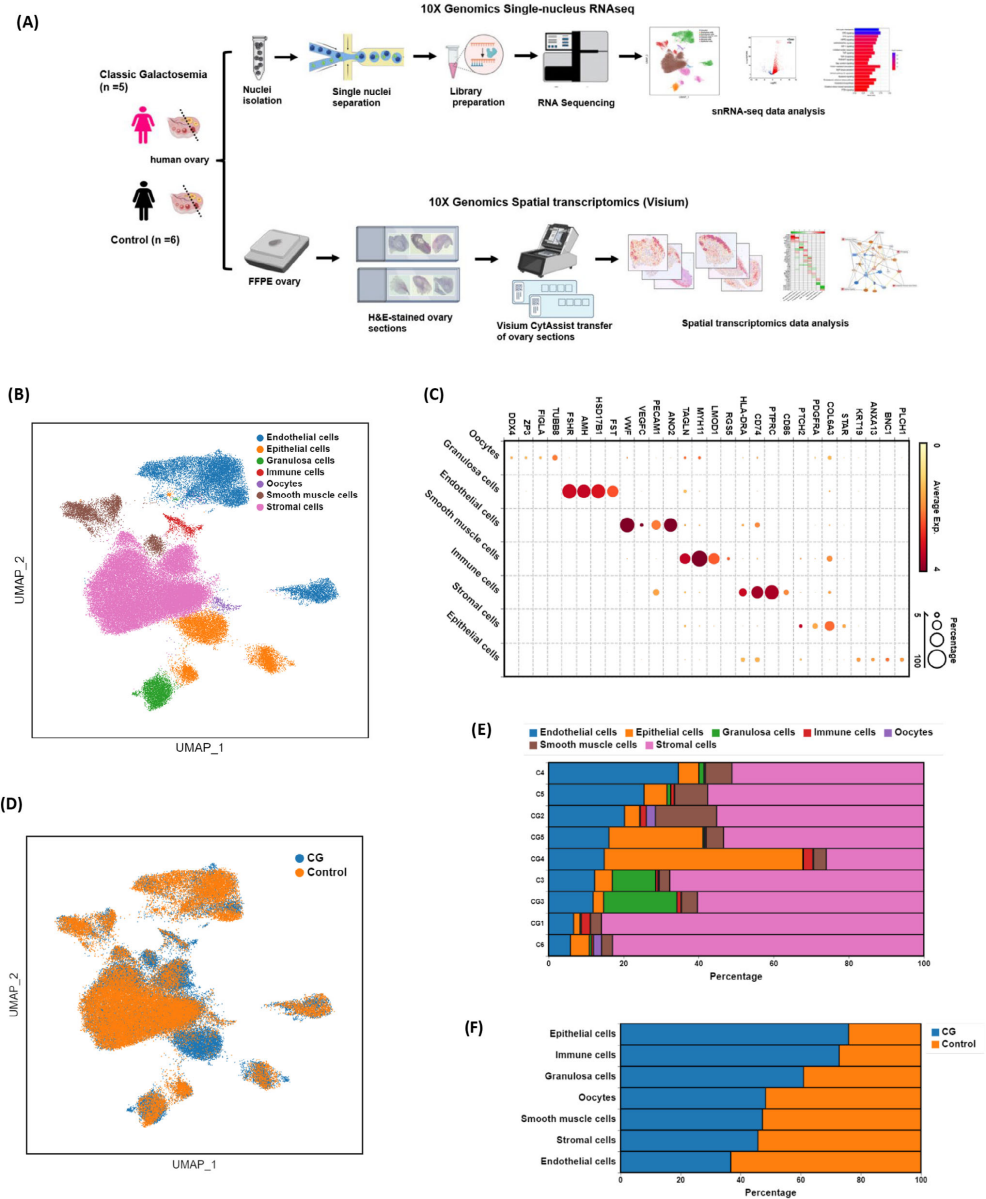
Single‐nucleus transcriptomic map of human ovaries from prepubertal females. (A) Schematic representation of the experimental workflow for snRNA‐seq and ST analysis of human ovaries from CG and control groups. (B) UMAP plot showing the distribution of the seven main distinct cell types of human ovarian tissues from all the samples. (C) Dot plot showing the expression levels of specific marker genes unique to each cell type identified in the human ovary atlas. (D) UMAP plot showing distribution of nuclei in the CG and control groups. (E) Box plot represents the percentage composition of each cell type among all the patient samples. (F) Box plot showing proportion of each cell type between CG and control groups.

We performed 10x Genomics snRNA‐seq on ovarian tissue biopsies collected from CG and control patients (prepubertal females) to create a single‐nucleus transcriptomic atlas of human ovaries (Figure 1A) and evaluated dynamic changes in gene expression pattern in specific cell types of our interest such as granulosa cells, oocytes, and stromal cells. The data from all the five CG and four control samples were combined for downstream analysis using Seurat‐based workflow. After quality control and filtering, a total of 81 603 nuclei, including 39 733 nuclei from CG samples and 41 870 nuclei from control samples were obtained for further analysis. Unbiased clustering and UMAP analysis revealed seven distinct main cell clusters (Figure 1B) based on the specific marker genes identified for each cell type shown in the dot plot (Figure 1C). A UMAP plot was drawn showing the distribution of all the nuclei obtained in both CG and control groups (Figure 1D). The major cell types identified based on the expression of signature marker genes were granulosa cells (*AMH*, *HSD17B1*), oocytes (*DDX4*, *TUBB8*), endothelial cells (*VWF*, *PECAM1*), smooth muscle cells (*TAGLN*, *MYH11*), immune cells (*CD74*, *PTPRC*), epithelial cells (*KRT19*, *PLCH1*), stromal cells (*DCN*, *COL3A1*; Figures 1C and S1). Furthermore, we performed GO analysis of the top 20 differentially expressed specific marker genes for each cell type to ensure the validity of cell clustering (Figure S2). For instance, the genes with high expression in granulosa cells were overrepresented in the GO term ‘female gonad development’ and GO terms ‘oocyte differentiation’ were specific to the oocyte cluster (Figure S2). GO terms including ‘extracellular matrix organisation’ and ‘collagen fibril organisation’ were enriched on stromal cells (Figure S2). We further quantified the relative proportions of these seven cell types in all the ovary samples and their distribution between the two conditions, CG and control groups. Among these cell types, stromal cells accounted for the majority of the proportion, and the lowest number of cells were identified as oocytes and immune cells (Figure 1E). We observed relatively similar percentage composition of different cell types identified in both CG and control groups except for immune cells and epithelial cells (Figure 1F).

### 3.2 Gene expression dynamics and altered signalling pathways in the granulosa cells of CG ovary

**FIGURE 2:**
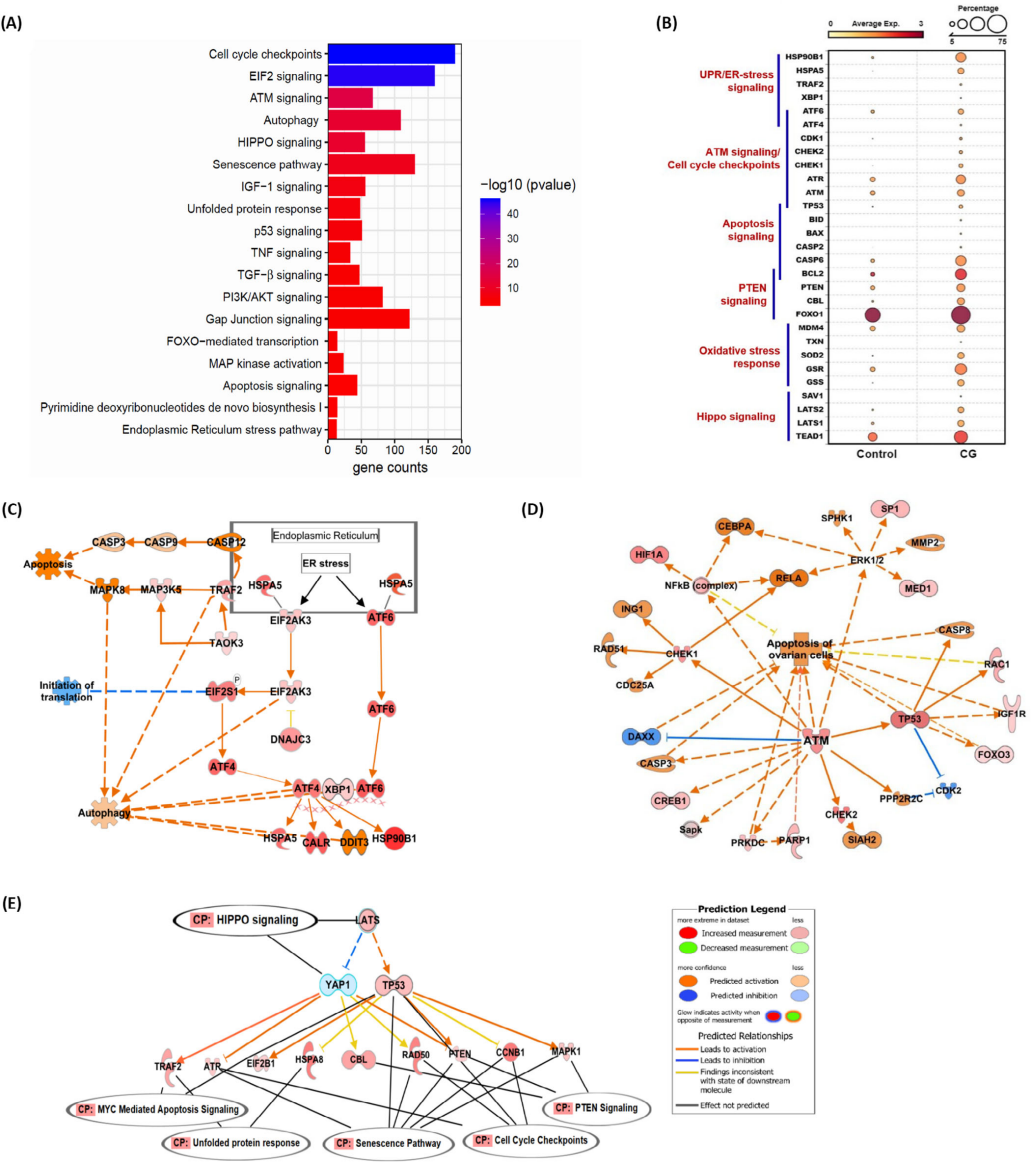
Changes in transcriptional profiles of granulosa cells in the CG ovary. (A) Bar graph of IPA analysis of DEGs in CG versus controls showing canonical signalling pathways and biological functions significantly enriched in the granulosa cell cluster. (B) Dot plot showing expression of key genes participating in different relevant pathways in granulosa cells between CG and control groups. (C) Causal network analysis showing HSPA5, ATF6, TRAF2 as upstream regulator genes involved in ER‐stress signalling pathway can promote apoptosis and autophagy of cell via direct or indirect interaction with their downstream target genes that were upregulated in the dataset. (D) Network analysis identified key genes in cell cycle checkpoint process (CHEK1 and CHEK2) which interact with other master regulator genes participating in ATM signalling and predicted to induce apoptosis of ovarian granulosa cells. (E) Network analysis showing upstream regulators (LATS and YAP1) of Hippo signalling interacting with other key genes participating in ATM signalling, PTEN signalling, senescence pathway, apoptosis signalling and unfolded protein response. The upstream regulators and downstream target genes are coloured by their predicted activation state: activated (orange) or inhibited (blue). The upstream regulators and their target genes in red colour indicates upregulated and green colour indicates downregulated in our snRNA‐seq dataset. Dotted lines indicate indirect interaction/regulation and straight line represents direct interaction/regulation with the target gene.

We compared the gene expression pattern of granulosa cells in CG and controls and performed functional enrichment analysis on DEGs using the Qiagen IPA tool to identify significantly enriched canonical pathways. Enrichment analysis revealed several signalling pathways and biological functions that were significantly altered in the CG group (Table S1). Some of the top 20 relevant pathways that were enriched in granulosa cells are shown in the bar graph (Figure 2A). Notably, we found expression of several key genes participating in endoplasmic reticulum (ER)‐stress signalling (*HSPA5, TRAF2, ATF4, ATF6, XBP1*), ATM signalling (*CHEK1, CHEK2, CDK1, ATM, ATR, TP53*), apoptosis signalling (*BID, BAD, CASP2, CASP9*), PTEN signalling (*PTEN*, *FOXO1*), hippo signalling (*TEAD1*, *LATS1*, *LATS2*, *SAV1)*, and oxidative stress pathways (*TXN*, *SOD1, SOD2*, *GSS, PXDR1*) were upregulated (Figure 2B) in the granulosa cells of CG. Interestingly, we also noticed expression of several genes crucial for cellular autophagy pathway were significantly increased in the CG (Figure S3). We performed Causal/regulatory network analysis on DEGs, which provided a comprehensive approach in identifying upstream regulator genes that regulate the expression of their downstream target genes in our snRNA‐seq dataset. We identified several potential upstream regulators (Table S2) in the DEGs list obtained from granulosa cell cluster. Evidently the network analysis on upstream regulator genes (*HSPA5*, *ATF6*, *TRAF2*) involved in ER stress signalling were shown to induce apoptosis and autophagic cell death, and halt translation initiation process through interactions with their downstream target genes which were upregulated in the CG group (Figure 2C). We also found several genes participating in autophagy pathway were upregulated. Another interesting observation was that cell cycle check point genes (*CHEK1* and *CHEK2*) and ATM signalling genes (*ATM*, *TP53, CASP3*) upregulated in the granulosa cells were shown to interact with each other, and predicted to promote apoptosis of ovarian cells in CG group (Figures 2D and S4). Further, we noticed an increased expression of p53 signalling network genes, which can induce apoptosis signalling and senescence pathway (Figure S5). More interestingly, we identified *LATS1* and *LATS2* genes as upstream regulators of hippo signalling that were upregulated in the granulosa cells of CG can regulate *YAP1* and *TP53* genes (Figure 2E). Network analysis predicted how these two master regulator genes (*YAP1* and *TP53*) can interact with their downstream target genes, which in turn regulate other signalling pathways such as ATM signalling, PTEN signalling, senescence pathway, apoptosis signalling and unfolded protein response (Figure 2E).

### 3.3 Changes in the transcriptomic profiles and signalling pathways enriched in the oocytes of CG ovary

**FIGURE 3:**
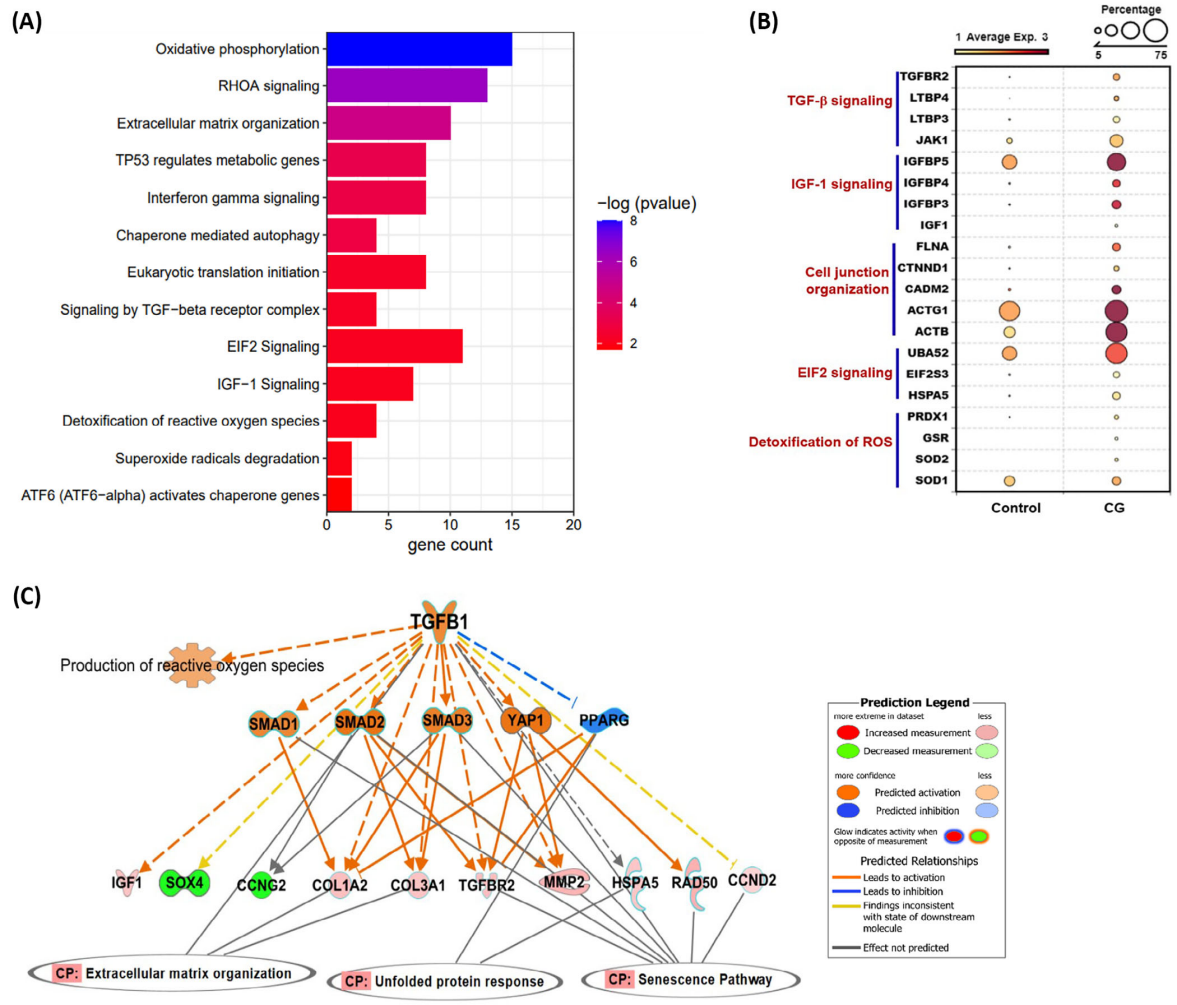
Gene expression dynamics in the oocyte cluster of CG ovary. (A) Bar graph of Qiagen IPA analysis of DEGs in CG versus controls showing canonical signalling pathways and biological functions significantly enriched in the oocytes. (B) Dot plot showing expression of key genes participating in different pathways in oocyte cluster between CG and control groups. (C) Causal network analysis predicted a key master upstream regulator TGFβ1 in the TGF‐β signalling network with intermediate regulators (SMAD1/2/3, YAP1 and PPRAG) that may explain up‐ and downregulation of downstream target genes found in our dataset, which can regulate ECM organisation, senescence pathway and unfolded protein response. The upstream regulators and downstream target genes are coloured by their predicted activation state: activated (orange) or inhibited (blue). The upstream regulators and their target genes in red colour indicates upregulated and green colour indicates downregulated in our snRNA‐seq dataset. Dotted lines indicate indirect interaction/regulation and straight line represents direct interaction/regulation with the target gene.

To investigate changes in gene expression between CG and control groups in oocyte cluster, we performed functional enrichment analysis on DEGs, which revealed top 13 canonical pathways that were significantly overrepresented (Figure 3A; Table S3). We identified several important genes that were altered in pathways such as TGF‐β signalling (*TGFBR2*, *LTBP3*, *LTBP4*), IGF‐1 signalling (*IGF1*, *IGFBP3/5/6*, *JAK1*), and EIF2 signalling (*EIF2S3*, *EIF4A1*, *HSPA5*) (Figure 3B). Biological functions such as oxidative phosphorylation (*ATP5F1B*, *MT‐ATP*6, *NDUFV1*, *UQCR10*) cell‐junction organisation (*ACTB1*, *ACTG1*, *FLNA*), chaperone mediated autophagy (*EEF1A1*, *UBA52*, *VIM*), and NRF2‐mediated oxidative stress response/detoxification of reactive oxygen species (ROS) (*GSR*, *SOD1*, *SOD2*, *PRDX1*) were upregulated in CG group (Figure 3B). Causal network analysis of DEGs from oocyte cluster identified many upstream regulators that regulate their downstream target genes participating in a signalling pathway (Table S4). Our study identified *TGFBR2* which is upregulated in oocytes of CG and network analysis suggests that the activation of *TGFβ1* can indirectly regulate UPR response, extracellular matrix (ECM) organisation and senescence pathway through downstream target genes in the TGFβ pathway, whose transcripts were significantly altered in the oocytes (Figure 3C). Interestingly, we also noticed higher expression of *TGFβ1* predicted to promote production of ROS (Figure 3C).

### 3.4 Characterisation and changes in the gene expression pattern of stromal cell subpopulations in CG ovary

**FIGURE 4:**
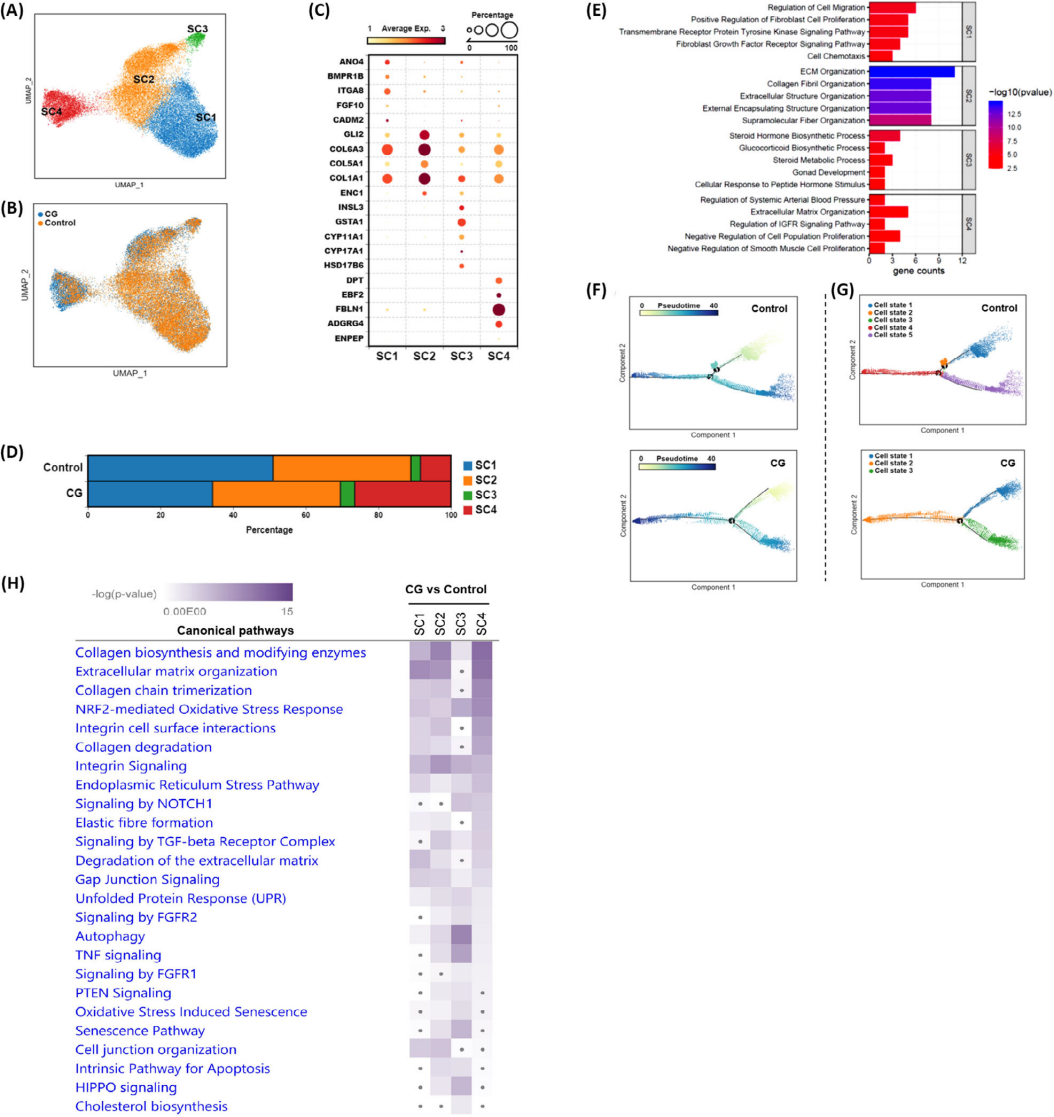
Characterisation and transcriptomic changes in human stromal cell subpopulations of CG ovary. (A) UMAP showing distribution of four different subpopulations of stromal cells. (B) UMAP plot revealing distribution of human ovarian stromal cells in CG and control patient samples. (C) Dot plot showing expression of unique marker gene identified in each stromal cell subtypes. (D) Box plot showing percentage composition of each stromal cell subtype in CG and control samples. (E) GO enrichment results for marker genes in SC1, SC2, SC3 and SC3 stromal subtypes. (F) Single‐cell trajectories of ovarian stromal cells as a function of the developmental pseudo timeline in CG and control ovaries. (G) Comparison of the distribution of stromal cell states in the control and CG groups along the pseudotime trajectories. (H) Qiagen IPA analysis of DEGs in CG versus control showing canonical signalling pathways and biological functions are significantly enriched in the stromal cell subtypes. Grey dots indicate pathways that are not significantly enriched.

Stromal microenvironment is essential for ovarian follicle growth and development as it provides structural support and is in constant communication with granulosa cells via paracrine signalling. Stromal cells (SCs), the predominant component of the ovarian stroma, can undergo differentiation into steroidogenic stromal cells, perifollicular stromal cells, fibroblast‐like cells and major extracellular matrix (ECM)‐producing stromal cells.^19^ Therefore, we were interested in delineating the characteristics of different SC subtypes and evaluate changes in gene expression patterns in the CG group compared to control group. Using sub‐clustering and UMAP analysis, we identified four subpopulations of SCs (Figure 4A) with distinct cellular transcriptomic signatures as shown in the dot plot (Figure 4C). The distribution of SCs in CG and control groups were shown in the UMAP plot (Figure 4B). Percentage composition of different SC subtypes showed relative lower number of SC1 and SC2 subtypes in the CG group compared to the control group (Figure 4D). We then performed GO enrichment analysis on specific marker genes to understand the biological functions in each subcluster of SCs (Figure 4E). Analysis of these markers showed that SC1 subtype with increased expression of *FGF10*, *ANO4*, *CADM2* and *ITGA8* was related to fibroblast‐like cells. The SC2 subpopulation was characterised by the expression of genes associated with the ECM producing cells, such as *COL1A1*, *COL3A, GLI2* and *COL5A1* (Figure 4E). SC3 subtype expressed marker genes like *CYP17A1*, *LHCGR, CYP11A1* and *HSD17B6* which are associated with steroidogenic stromal cells (Figure 4E). The SC4 subpopulation was defined by the expression of *ENPEP*, *ADGRG4, FBLN1, DPT* genes that are associated with perifollicular stromal cells, which have been previously reported (Figure 4E).^20^ The distributions and expression of most significant marker genes in each subpopulation of stromal cell type are outlined in the Figure S6. SC1 and SC2 represent majority of the stromal subpopulations that provide structural support to the stroma in the ovary (Figure 4D). Next, we performed Pseudotime trajectory analysis to identify the differentiation heterogeneity of stromal subpopulations between CG and control groups. Interestingly, trajectory analysis revealed two differentiation branch points with cell differentiation trajectories of SCs comprised of five cell states in the control group, and contents of four SC subtypes differed in various states. While in CG group the stromal cells trajectory showed one branch point that comprised of three cell states (Figure 4F and G). Furthermore, we analysed alterations in gene expression pattern in SC subtypes by performing functional enrichment analysis on DEGs from each SC subpopulation which revealed several pathways and biological functions that are highly enriched. Especially, biological functions that were significantly altered in SC1, SC2 and SC4 subtypes of the CG group which included ECM organisation, degradation of collagen, integrin cell surface interactions, degradation of the ECM, autophagy, NRF2‐mediated oxidative stress response and unfolded protein response (Figure 4H). We also found expression of genes participating in gap junction signalling, integrin signalling, TGF‐β signalling, FGFR2 signalling and Hippo signalling, PTEN signalling, senescence pathways were also significantly changed in the SC2 and SC3 subtypes of the CG group (Figure 4H). We noticed cholesterol biosynthesis pathway was specifically altered in SC3 subpopulation which is known to be associated with steroidogenesis process (Figure 4H).

### 3.5 ST analysis reveals dysregulated signalling pathways essential for primordial follicle growth and survival

**FIGURE 5:**
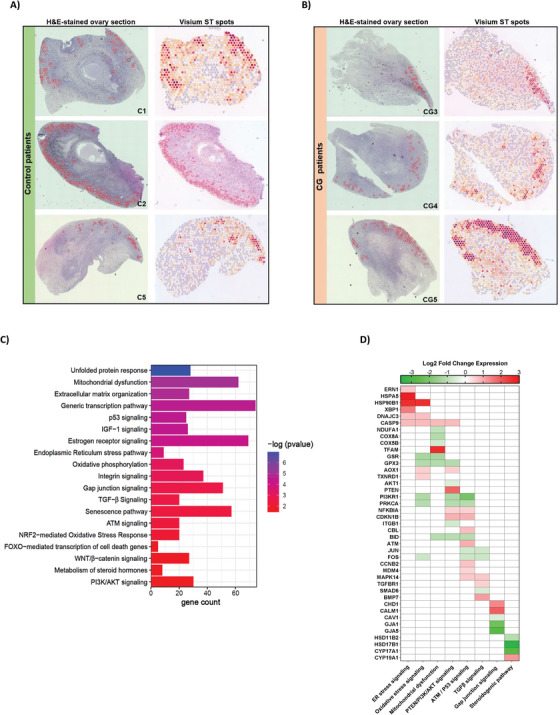
Spatial transcriptomic analysis of human ovarian follicles in CG and control patients. (A) Control samples (*n* = 3) (B) CG samples (*n* = 3). Left panel showing H&E‐stained ovarian sections. The red circles identify early‐stage follicles (primordial, transitional primordial, and primary). Right panel represent Visium capture area showing spatial distribution (ST spots) of ovarian follicles, with orange to dark red spots showing the combined expression of follicle specific marker genes (*DDX4, ZP2, FIGLA, TUBB8, AMH, HSD17B1* and *FST*). (C) Qiagen IPA analysis of DEGs from ovarian follicles in CG versus control showing canonical signalling pathways and biological functions that were significantly enriched. (D) Heatmap representing a list of up‐/downregulated important genes participating in relevant pathways that were enriched in follicles of CG group.

**FIGURE 6:**
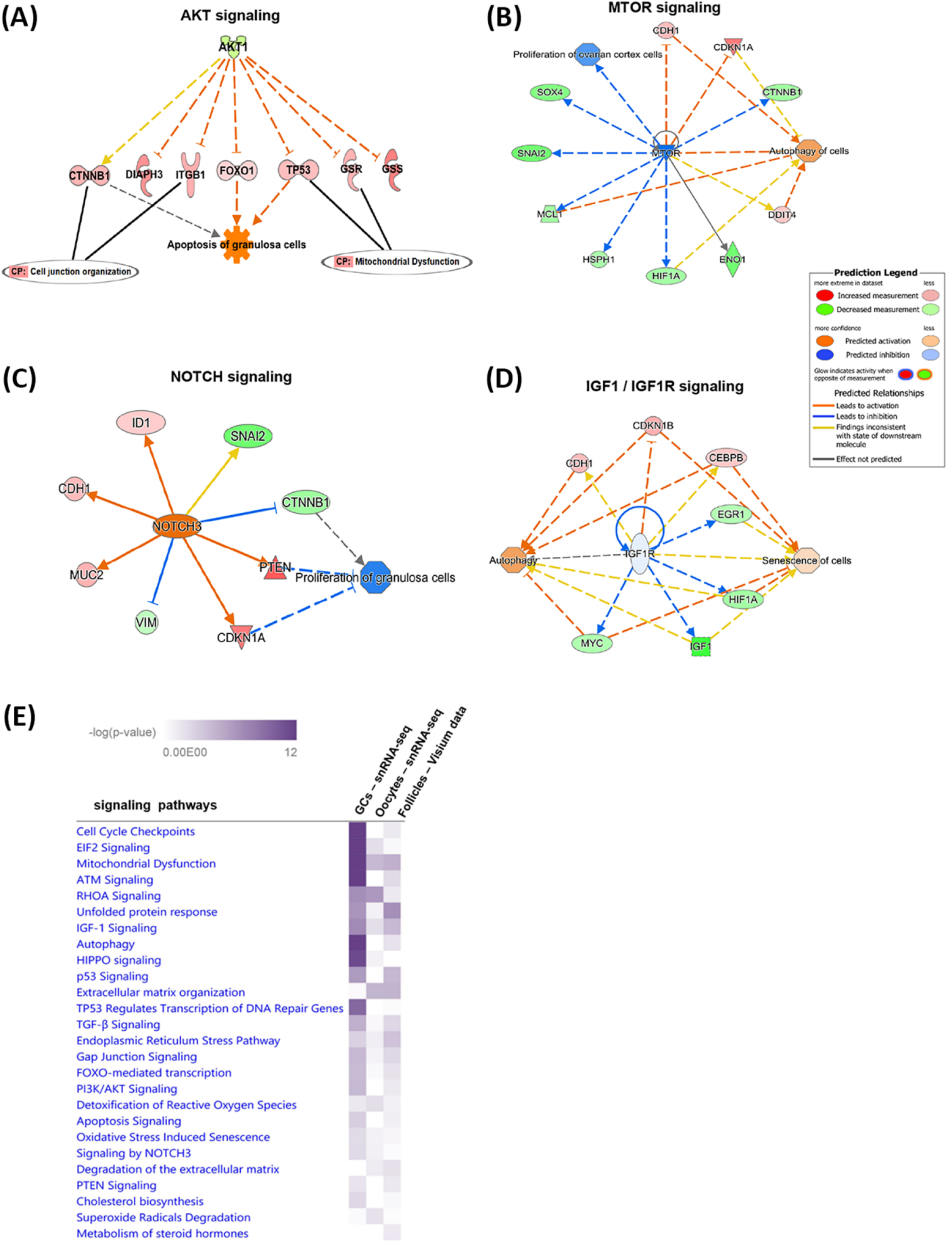
Dysregulated/altered signalling pathways essential for primordial follicle activation, development, and survival. (A) Causal network analysis identified AKT1 as a key upstream regulator gene in PI3K/AKT signalling which can induce apoptosis of granulosa cells via FOXO1 transcription factor. (B) Predicted downregulation of MTOR signalling network can inhibit proliferation of ovarian cortex cells and promote autophagy of cells via their effector genes. (C) Predicted upregulation of NOTCH3 involved in NOTCH signalling may hinder proliferation of granulosa cells. (D) IGF‐1/IGF1R signalling was predicted to be downregulated which can promote autophagy and senescence of cells. (E) IPA comparison analysis of snRNA‐seq and ST datasets showing a list of overlapping and unique signalling pathways and biological functions that were significantly enriched. GCs: granulosa cells.

To corroborate with our snRNA‐seq findings, we created ST landscape of paediatric ovarian tissue sections from CG (*n* = 3) and control (*n* = 3) samples using 10x Visium spatial gene expression method. We spatially identified ST spots corresponding to ovarian follicles (primordial, transitional primordial, and primary) on the capture area of Visium slide based on the specific marker genes for granulosa and oocytes detected in the snRNA‐seq data (Figure 5A and B). Each red to purple ST spot identified as ovarian follicle represents enriched expression of combined signature marker genes such as *DDX4, ZP2, FIGLA, TUBB8, AMH, HSD17B1* and *FST* (Figure 5A and B). We then clustered together ST spots corresponding to ovarian follicles and performed differential expression and enrichment analyses between CG and control groups. The top 20 functionally enriched signalling pathways (Table S5) obtained using IPA canonical pathway analysis tool were shown in the bar graph (Figure 5C). The IPA pathway analysis revealed ER‐stress signalling associated genes (*HSPA5*, *ERN1*, *XBP1*, *CASP9*, *ATF4*), oxidative stress response genes (*AOX1, TXNRD1*), PTEN signalling genes (*PTEN, CASP9, CBL*) and ATM signalling genes (*ATM, BID, CCNB1, JUN, NFKBIA*) were upregulated (Figure 5D). Steroidogenic pathway genes (*CYP17A1, HSD17B1, HSD11B1*), gap‐junction signalling genes (*GJA1*, *GJA5*, *CAV1*), PI3K/AKT signalling genes (*AKT1*, *PRKCA*, *ITGB1*, *BID*) and IGF‐1 signalling genes (*IGF‐1*, *JUN*, *FOS*, *MRAS*, *ELK1*, *CCN1*) were downregulated (Figure 5D). Interestingly, we also observed antioxidant gene such *GSR*, *GPX3*, and *GPX7* that protect the cells from ROS by maintaining redox balance were downregulated, which can lead to more accumulation of ROS in follicles (Figure 5D). We performed causal network analysis on DEGs, which identified 330 potential upstream regulator genes (Table S6). Casual network analysis showed downregulation of *AKT1* participating in PI3K/AKT signalling can induce apoptosis of granulosa cells via *FOXO1* transcription factor (Figure 6A). We found key regulator genes like *MTOR* (mTOR signalling) and *NOTCH3* (Notch signalling) can directly or indirectly affect the proliferation of ovarian cortex cells and granulosa cells, respectively (Figure 6B and C). *IGF1R* participating in IGF‐1 signalling was predicted to be downregulated and can promote autophagy and senescence of cells (Figure 6D). Furthermore, we compared both snRNA‐seq and ST datasets using IPA comparison analysis tool. Comparison analysis revealed mitochondrial dysfunction, unfolded protein response, gap junction signalling, ER stress, PI3K/AKT, IGF‐1 and oxidative stress induced senescence pathways were significantly altered in both in snRNA‐seq and ST datasets (Figure 6E). We noticed genes involved in steroid metabolism and degradation of ECM were more significantly enriched in ST dataset. Hippo, ATM and EIF2 signalling pathways were highly enriched in snRNA‐seq dataset from granulosa cells (Figure 6E). Network analysis of some DEGs that were identified both in snRNA‐seq and ST datasets showed crosstalk among PTEN/PI3K/AKT signalling genes with other pathways including ER‐stress, apoptosis, autophagy, ATM and senescence signalling pathways (Figure S7).

### 3.6 Cell‐cell communication using CellChat identifies intercellular signalling changes among different cell types

**FIGURE 7:**
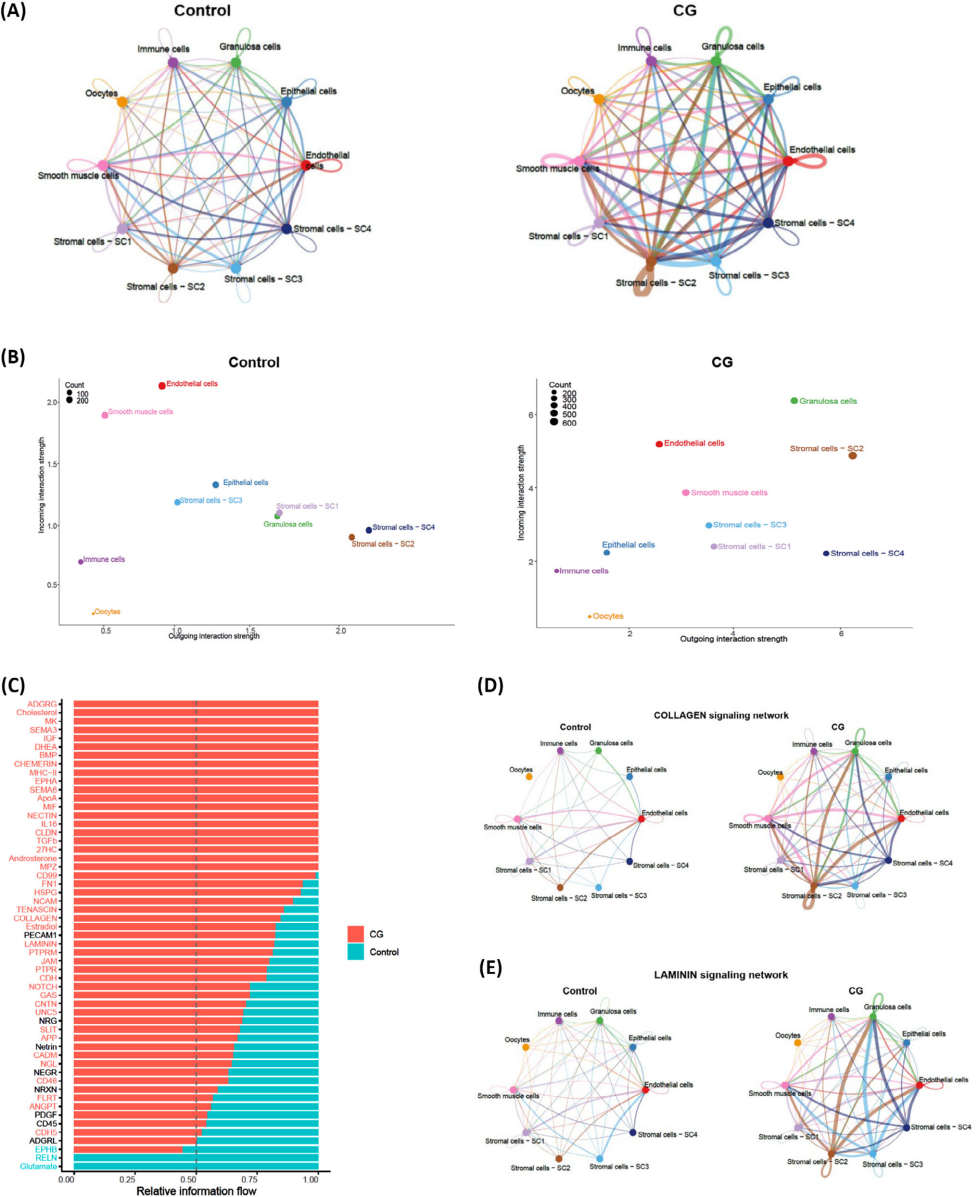
Cell‐cell communication analysis identifies intercellular signalling changes among different cell types. (A) Circle plots showing the total numbers of interactions (ligand‐receptor pairs) among different cell populations between control and CG ovaries. (B) Scatter plots showing the outgoing and incoming interaction strength comparison in 2D among different cell types between control and CG. (C) Bar plot showing the comparison of different signalling pathways based on the relative information flow between control and CG groups. (D) Circle plot depicting COLLAGEN signalling pathway network among different cell types in control and CG. (E) Circle plot portraying LAMIMIN signalling network among different cell types in control and CG.

To explore how the cell‐to‐cell communication changes between control and CG, we first compared the number of implied ligand‐receptor pair interactions among different cell types. Interestingly, we found that the total number of interactions among 6 distinct cell types and 4 stromal cell subpopulations was relatively higher in CG compared to control (Figure 7A). To understand the communications between which cell type were substantially altered, we compared the outgoing and incoming signals of each cell type in control and CG. We noticed that both the outgoing and incoming interaction strength from granulosa, endothelial, smooth muscle, stromal‐SC1, ‐SC2, ‐SC3 and ‐SC4 cells were much higher in CG compared to control (Figure 7B). Next, we matched the overall relative information flow for each signalling pathway between control and CG groups. We observed that, in CG a larger number of the signalling pathways were greatly enriched compared to control (Figure 7C). Some of these signalling pathways that showed greater information flow are involved in ECM organisation and integrin signalling such as COLLAGEN, FLRT, LAMININ, NECTIN, FN1 and TENASCIN which is evident from the strength of outgoing signalling patterns of each cell type (Figure 7C). Further, we specifically compared cell to cell communication network of the COLLAGEN and LAMININ signalling. We observed that these signalling pathways have more interactions and stronger signal strength among different cell types. COLLAGEN signalling was stronger in CG among SC1, SC2, SC4, granulosa, endothelial and smooth muscle cells (Figure 7D). We found that outgoing LAMININ signalling was stronger from SC2, SC3 and SC4 to granulosa and endothelial cells in the CG group (Figure 7E).

### 3.7 Immunohistochemical analysis revealed changes in phospho‐AKT, phospho‐EIF2A, phospho‐H2A.X, LC3A/B and CASP9 expression in the primordial follicles of CG ovary

**FIGURE 8:**
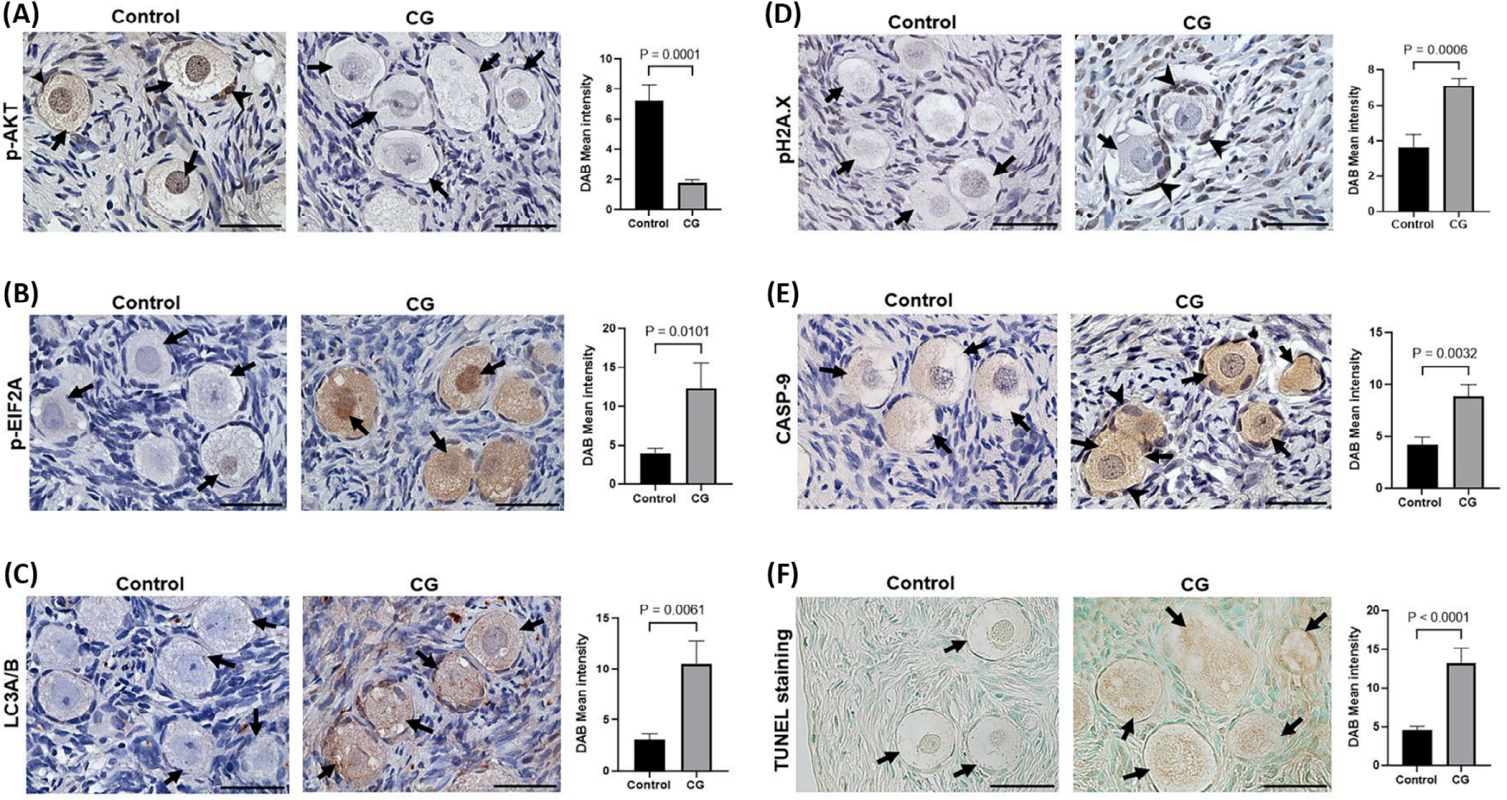
Immunohistochemical analysis of phospho‐AKT and CASP9 expression in the ovary. Representative IHC stained images showing expression of phospho‐AKT (A), phospho‐EIF2A (B), LC3A/B (C), phospho‐H2A.X (D), CASP9 (E), and TUNEL‐positive staining (F) in the primordial follicles of the ovary from CG and control groups. Arrows indicates primordial follicles and arrowheads indicates granulosa cells. Data were represented as bar plots showing DAB signal intensity (mean ± SEM; *n* = 3) and analysed by unpaired Student's *t*‐test.

We performed immunostaining on ovarian sections from CG and control patients to evaluate the protein expression of the following: (1) phospho‐AKT, which is indictive of activated PI3K/AKT signalling, (2) phospho‐EIF2A as a marker for ER stress response, (3) phospho‐H2A.X, a DNA damage marker, (4) LC3A/B to assess autophagy and (5) CASP9, a well‐known initiator caspase which triggers intrinsic apoptosis. IHC analysis revealed phospho‐AKT expression in both granulosa cells and cytoplasm of the oocytes which varied among the primordial follicles. We quantified the DAB immunosignal intensity, which showed a significant reduction in the phospho‐AKT signal in the follicles of CG patients compared to controls (Figure 8A). We also noticed a strong phospho‐AKT signal in the nucleus of several oocytes of controls, suggesting its translocation to the nucleus after phosphorylation (Figure 8A). The expression of phospho‐EIF2A was significantly higher in primordial follicles indicating profound ER‐stress response in CG ovaries (Figure 8B). We found signs of increased autophagy and DNA damage in the oocytes and granulosa cells of primordial follicles as evidenced by marked increase in expression of phospho‐H2A.X and LC3A/B in the CG ovaries (Figures 8C and D and S8). We detected significantly increased expression of CASP9 in primordial follicles of CG ovaries, suggesting an accelerated rate of apoptosis compared to controls (Figure 8E). In addition, TUNEL assay also revealed increased apoptosis of primordial follicles (Figure 8F) and stromal cells (Figure S8) in the CG compared to control ovaries. These IHC findings corroborate with our snRNA‐seq and ST transcriptomics data, where we found increased expression of gene associated with apoptosis and autophagy pathways, and dysregulated PI3K/AKT signalling that can have an adverse effect on primordial follicle activation and survival in the CG patients.

### 3.8 D‐galactose toxicity induces DNA damage and apoptosis of ovarian follicles in mouse ovary cultures

To assess the effect of D‐galactose toxicity on ovarian follicles mouse whole ovary cultures were treated with and without 5 and 10 mM concentration of D‐galactose solution for 48 h. We performed IHC analysis on ovary sections to check the expression of DNA damage marker phospho‐H2A.X and apoptotic cell‐death marker cleaved‐CASP3. We found a significant increase in the expression of phospho‐H2A.X and cleaved‐CASP3 in the granulosa cells of D‐galactose treated ovaries compared to controls, suggesting galactose toxicity can induce apoptosis of granulosa cells (Figure S9).

## 4 DISCUSSION

Our recent histological data analysis of ovaries from prepubertal female children diagnosed with CG showed substantial reduction in mean follicle density with a remarkable decline in the primordial follicle pool leading to POI.^14^ Moreover, the CG patients have no follicles beyond transitional primordial stage in contrast to control patients, which exhibit normal follicular activation/growth from primordial follicle to transitional primordial stage and then to primary follicle stage.^14^ Therefore, to understand the molecular mechanism underlying early onset of POI in these CG patients, we performed snRNA‐seq and Visium ST on ovarian tissue biopsies obtained from prepubertal girls with CG and compared with control patients of similar age that had ovarian tissue removed prior to gonadotoxic treatment for alternate diagnoses. The snRNA‐seq analysis of human ovary identified signature maker genes for seven distinct types of ovarian cells that showed similarities with previously published single‐cell RNA‐seq data in human ovaries.^21^, ^22^

Our snRNA‐seq analysis of granulosa cells and oocytes and ST data from ovarian follicles in the CG group revealed dysregulation of several molecular signalling pathways such as ER‐stress signalling, ATM signalling, oxidative stress signalling, apoptosis signalling, PTEN/PI3K/AKT signalling, TGFβ signalling, IGF‐1 and Hippo signalling. Among these signalling pathways, we found important ER‐stress related genes including *EIF2S1*, *HSPA5*, *XBP1*, *TRAF2*, *ATF4* and *ATF6* were profoundly upregulated in the granulosa cells of CG. In our IPA causal network analysis, we found upstream regulator genes such *ATF6*, *HSPA5*, *TRAF2* involved in ER stress signalling were upregulated, which can promote apoptosis and autophagy of granulosa cells via activation of downstream target genes. ST data on ovarian follicles identified few additional genes such as *ERN1* and *ATF3* that were significantly upregulated in the CG indicating ER stress response. ER stress signalling activates an adaptive unfolded protein response to restore protein homeostasis, however, it can trigger apoptosis, autophagy of cells during prolonged stress.^23^, ^24^ Additionally, both in the granulosa cells and oocytes of the CG group, several key genes (*SOD1*, *SOD2*, *PRDX1*, *TXN*, *GSR*, *GSS*) participating in oxidative stress response involving detoxification of ROS were upregulated. Similarly, ST data from ovarian follicles noted that antioxidant genes (*AOX1*, *TXNDR1*, *GSR*, *GPX3*, GPX4) involved in oxidative stress pathway were differentially expressed, and the expression of other genes such as *NDUFA1*, *COX8A*, *COX5B* and *TFAM* involved in mitochondrial dysfunction were also affected. Furthermore, we found several genes involved in oxidative phosphorylation were upregulated in the oocyte cluster of the CG group. This is consistent with studies indicating that both mitochondrial dysfunction and increased oxidative phosphorylation is often associated with more ROS and other free radical production which can lead to cell damage and DNA damage in ovarian follicles.^25^, ^26^, ^27^ It has been well established that oxidative stress can trigger ER stress response and mitochondrial dysfunction. This redox imbalance‐induced ER stress response can progress to cell death and autophagy, and eventually lead to several pathophysiological changes.^24^, ^28^ Additionally, oxidative stress can also induce DNA damage in cells triggering the ATM signalling pathway implicated in DNA damage repair, cell cycle arrest, and apoptosis.^29^, ^30^, ^31^ This was evident from our snRNA‐seq results, where we noticed genes such as *CHEK1*, *CHEK2*, *ATM*, *TP53*, and others involved in ATM signalling and cell cycle regulation by checkpoints were significantly enriched in the CG group. Moreover, our IHC analysis also revealed significantly increased expression of phospho‐EIF2A, H2A.X and LC3A/B in primordial follicles in the CG ovaries suggesting ER‐stress response, DNA damage and autophagy of cells, respectively.

**FIGURE 9:**
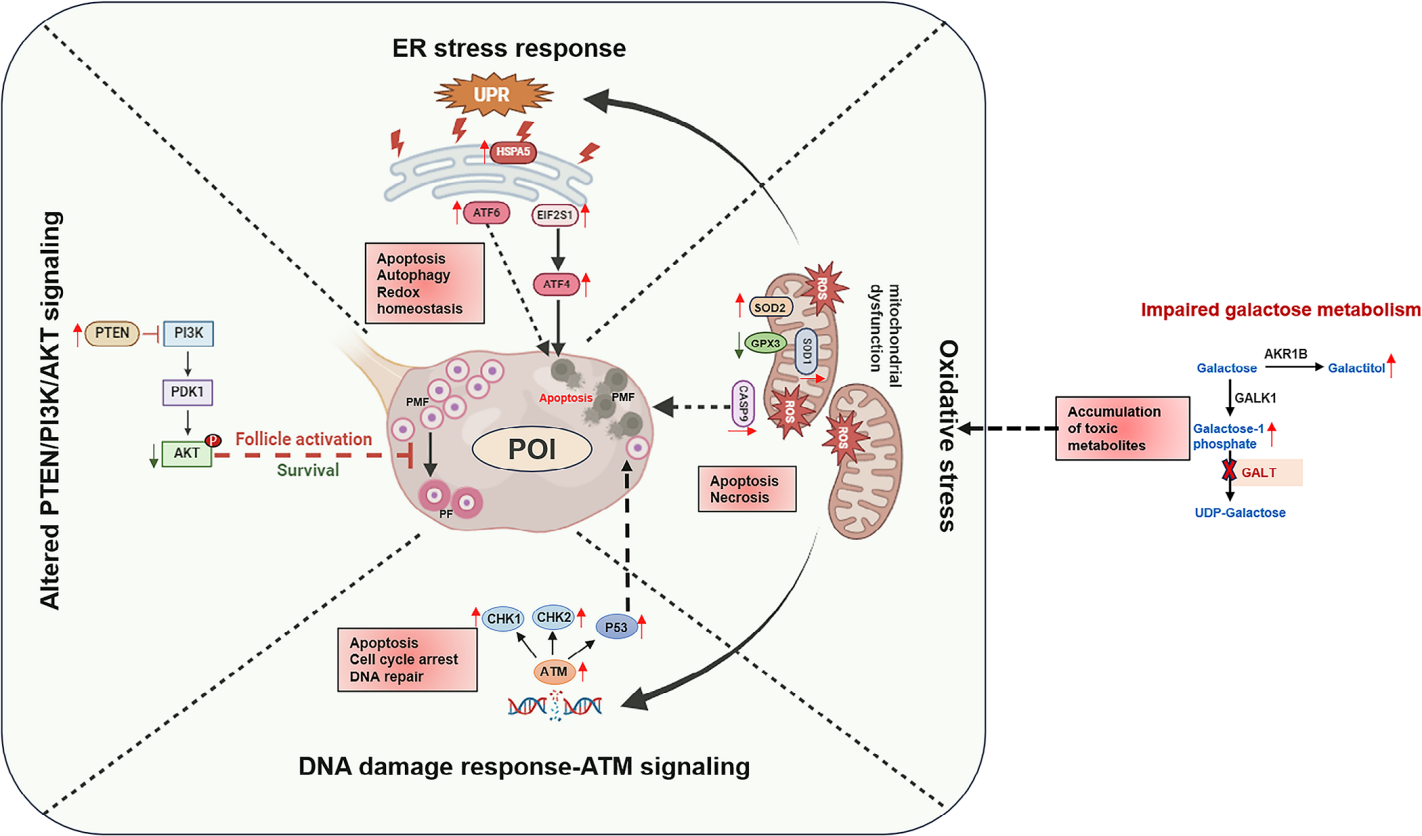
The proposed molecular mechanism of POI in CG patients. The accumulation of toxic metabolites such as galactitol and galactose‐1‐phosphate could induce oxidative stress/mitochondrial dysfunction via increased ROS production in the ovary of CG. This in turn activates the ER stress signalling leading to apoptosis and autophagy of follicles. Oxidative stress can also cause DNA damage in cells thereby activating ATM signalling as evident by increased expression of ATM, CHEK1/2 genes leading to cell arrest/ apoptosis of follicles. Furthermore, dysregulated PI3K/AKT signalling due to reduced phospho‐AKT and increased PTEN can hinder follicle activation and survival. All together these dysfunctional signalling pathways may gradually initiate early onset of POI in prepubertal CG patients. PMF: primordial follicles, PF: primary follicles, POI: premature ovarian insufficiency.

Clinical studies have indicated that impaired galactose metabolism leads to high levels of galactitol and galactose‐1‐phosphate metabolites in the blood and urine of CG patients. The intracellular increase in galactitol induces hyperosmotic and oxidative stress which is responsible for the onset of cataracts in CG patients with *GALT* and *GALK* gene mutations.^32^ An increasing amount of evidence suggests that galactose toxicity can induce senescence in dermal fibroblasts via oxidative stress and mitochondrial impairment.^33^ In mice, D‐galactose and its metabolites were shown to reduce oocyte quality through significant enhancement in production of ROS.^34^ In this study, using in vitro mouse ovary cultures, we showd that D‐galactose toxicity can significantly induce apoptosis and DNA damage in granulosa cells. in vitro experiments in rats also showed toxic effects of galactose lead to increased granulosa cell expression of *TP53* and follicular atresia.^35^ Interestingly, we also noticed upregulation of *TP53* gene in the granulosa cells of CG which is known to trigger intrinsic cell death pathways for the induction of follicular atresia.^36^ Based on our findings from this study, it is plausible that accumulation of these toxic metabolites (galactitol and galactose‐1‐phosphate) can induce oxidative stress and mitochondrial dysfunction via ROS production which in turn trigger ER stress response and ATM‐dependent DNA damage signalling pathways, finally leading to autophagy, apoptosis, and cell arrest of granulosa cells and oocytes in the CG condition (Figure 9).

More importantly, we also noticed both PTEN/PI3K/AKT signalling and Hippo signalling pathways were greatly impaired/dysregulated in the CG group. These two pathways are most well‐studied pathways in the context of ovarian follicle growth and development.^37^, ^38^, ^39^, ^40^, ^41^ PTEN/PI3K/AKT signalling and the Hippo signalling are two critical regulators of primordial follicle quiescence, activation, growth, and survival. In our study, causal network analysis identified that Hippo signalling genes *LATS1* and *LATS2* were upregulated in CG granulosa cells. These two kinases predicted to inhibit YAP and activate TP53, which in turn regulate other canonical pathways through their downstream target genes. Conditional knockout of the *YAP* gene in granulosa cells of mice resulted in abnormal ovarian follicle development and atresia.^42^ It is also noteworthy that *PTEN* was upregulated in the follicles of CG as evident from our snRNA‐seq and ST data. It is well known that PTEN is a negative regulator of PI3K by inhibiting phosphorylation of AKT protein kinase.^41^, ^43^ Activation of AKT via phosphorylation promotes follicle survival by inhibiting proapoptotic proteins and activating prosurvival proteins.^40^, ^41^ In our snRNA‐seq data, we found proapoptotic genes such as *BAX*, *BAD*, *FOXO3* were upregulated in granulosa cells. Our IHC analysis revealed significant reduction in the phosphorylated AKT protein expression in the primordial follicles of CG patients, suggesting impaired follicle activation and survival. Lack of follicle activation is supported by our recent data where almost no activated follicles (primary and secondary) beyond transitional primordial were noted in the CG ovaries indicating failure of primordial follicle activation.^14^ Further, the AKT1‐KO murine study revealed significantly fewer primordial follicles leading to infertility.^44^ In our network analysis, we also identified downregulation of *AKT1*, an important upstream regulator involved in PI3K/AKT signalling that can induce apoptosis of granulosa cells via *FOXO1* transcription factor.

Additionally, we noticed increased expression of *FOXO1* and *FOXO3* transcripts in CG transcriptome profiles. In mammalian ovaries, the FOXO1/3 proteins regulate follicular growth and atresia by promoting induction of apoptosis in granulosa cells.^45^, ^46^ It has been reported that increased *FOXO1* expression in granulosa cell can lead to apoptosis which is induced by oxidative stress.^46^ All these findings suggest that hypoactivation or downregulation regulation of the PI3K/AKT/FOXO pathway can impede follicle activation and may result in excessive primordial follicle atresia via apoptosis. This was further supported by our IHC data where we showed increased expression of CASP9 protein and more TUNEL staining in the primordial follicles of CG group that can lead to increased follicular atresia. Additionally, we noticed IGF‐1, MAPK, and TGF‐β signalling pathways implicated in ovarian folliculogenesis were also impaired in the CG group. The crosstalk between the MAPK and IGF‐1 signalling pathways seems to be linked to PI3K/AKT signalling in the ovary and is vital for the activation of primordial follicles.^47^, ^48^ The pathogenesis of POI has been associated to MAPK signalling, and the inhibition of this pathway has shown promising results in improving ovarian outcomes.^49^ IGF‐1 is a follicular survival protein, capable of activating MAPK and PI3K/AKT signalling, and protects follicles against apoptosis in the ovary.^49^ There is evidence suggesting that IGF‐1 signalling is impaired in CG and through its receptor it can mediate autophagy. Galactose‐1‐phosphate effectively decreased the expression of the IGF‐1 gene in fibroblast cultures obtained from healthy neonates.^50^ Therefore, it is possible that the IGF‐1 signalling impairment we found in our ST data can promote excessive follicle atresia in CG through autophagy.

SCs are a type of connective tissue that surround follicles and make up the majority of the ovarian stroma. In addition to their intricate interaction with the follicles via complex paracrine signalling, SCs provide structural and steroidogenic support for follicle growth. SCs may also play an important role in primordial follicle activation and differentiation of steroidogenic theca cells.^21^, ^51^ Follicles and ovarian stromal cells secrete enzymes to soften the surrounding ECM and allow for follicular expansion. ECM components (collagen types I/III/IV/VI, fibronectins, laminins and proteoglycans) play a crucial role in regulating cell functions including adhesion, migration, and proliferation through both direct and indirect signalling.^21^, ^51^ SCs which accounted for most of the somatic cells in our scRNA‐seq data were altered dramatically in both number and cellular status in the CG group. We characterised 4 different subpopulations of SCs with distinct cellular transcriptomic signatures that were also identified as marker genes in previously reported human ovary single‐cell atlas.^20^, ^51^, ^52^ There were significant changes in gene expression profiles largely in SC1, SC2, and SC4 subtypes in the CG group. Enrichment analysis revealed several key genes involved in ECM signalling, integrin signalling, collagen biosynthesis/assembly, and gap‐junction signalling were impaired. Moreover, cell‐cell communication analysis using CellChat revealed NECTIN, FN1, TENASCIN, LAMININ, COLLAGEN signalling pathways were significantly enriched in CG ovaries and their signal strength. Apparently, a dysregulation/imbalance in ECM or integrin or gap‐junction signalling has an adverse impact on ECM composition and structure affecting stiffness of ovarian cortex where primordial follicles reside.^53^, ^54^, ^55^ Additionally, our snRNAseq data showed that cellular stress related pathways were activated including NRF2‐mediated oxidative stress signalling and ER‐stress signalling in all four subpopulations of SCs. Cholesterol biosynthesis was significantly altered only in SC3 subtype.

In conclusion, our work successfully resulted in a comprehensive single‐nucleus atlas and spatial landscape of paediatric ovarian tissues, thoroughly examining changes in gene expression profiles in ovarian follicles and stromal cells between prepubertal CG and control females. Our findings from snRNA‐seq and ST analysis of the human ovary from prepubertal girls with CG revealed an increased activation of cellular stress pathways such as ER‐stress signalling and oxidative stress response that can trigger DNA damage, apoptosis, and autophagic cell death of ovarian follicles. We also noticed changes in the transcriptome profiles in four different subpopulations of stromal cells, where genes participating in ECM organisation, collagen assembly, integrin signalling and gap‐junction signalling pathways were significantly impaired. Furthermore, we showed downregulation of PI3K/AKT signalling as supported by reduced phospho‐AKT expression and increased expression of CASP9 can lead to failure in the primordial follicle activation/development and early atresia. Taken together, our data suggests dysregulation in the above‐mentioned signalling pathways that are crucial for ovarian follicle development and survival can eventually lead to early onset of POI in CG (Figure 9). Understanding the etiology of POI in prepubertal girls with CG may have implications in the development of future therapeutic interventions to preserve ovarian function and promote women's reproductive health.

## AUTHOR CONTRIBUTIONS

Veronica Gomez‐Lobo and Jacqueline C. Yano Maher conceptualised this project, performed the surgeries on both the CG and control patients, reviewed and edited the manuscript. Raghuveer Kavarthapu, Mary E. Soliman, Hong Lou, Taylor Badger, Ramya Balasubramanian, Victoria Huyhn, and Maria De La Luz Sierra performed snRNA‐seq, Visium experiments and IHC experiments. RK, Han Do and Thang Pham performed bioinformatic analysis and interpreted the transcriptome data. RK analysed overall data, wrote and edited the manuscript. RK, VGL and JCY verified the underlying data reported in this work. All authors have read and approved the final manuscript.

## CONFLICT OF INTEREST STATEMENT

Authors declare no conflict of interest.

## ETHICS STATEMENT

This study was conducted in accordance with the Institutional Review Board of Eunice Kennedy Shriver National Institute of Child Health and Human Development (NICHD; protocol numbers IRB000106, IRB00715) at National Institutes of Health and at Children's National Hospital in Washington, DC (IRB protocol numbers Pro00010699, Pro00016433). All patients provided written informed consent.

## Supporting information



Supporting information

Supporting information

Supporting information

Supporting information

Supporting information

Supporting information

Supporting information

## Data Availability

RNA‐sequencing data files from snRNA‐seq and spatial transcriptomics analysis are deposited in Gene Expression Omnibus (GEO) with accession number GSE267932 and GSE268523.

## References

[1] Wada Y , Kikuchi A , Arai‐Ichinoi N , et al. Biallelic GALM pathogenic variants cause a novel type of galactosemia. Genet Med. 2019;21:1286‐1294. 30451973 10.1038/s41436-018-0340-x

[2] Hagen‐Lillevik S , Rushing JS , Appiah L , et al. Pathophysiology and management of classic galactosemic primary ovarian insufficiency. Reprod Fertil. 2021;2:R67‐R84. 35118398 10.1530/RAF-21-0014 PMC8788619

[3] Fridovich‐Keil JL , Berry GT . Pathophysiology of long‐term complications in classic galactosemia: what we do and do not know. Mol Genet Metab. 2022;137:33‐39. 35882174 10.1016/j.ymgme.2022.07.005

[4] Jumbo‐Lucioni PP , Garber K , Kiel J , et al. Diversity of approaches to classic galactosemia around the world: a comparison of diagnosis, intervention, and outcomes. J Inherit Metab Dis. 2012;35:1037‐1049. 22450714 10.1007/s10545-012-9477-y PMC3774053

[5] Pyhtila BM , Shaw KA , Neumann SE , Fridovich‐Keil JL . Newborn screening for galactosemia in the United States: looking back, looking around, and looking ahead. In JIMD Reports. 2015;15:79‐93. 10.1007/8904_2014_302 PMC4413015 24718839

[6] Fridovich‐Keil JL , Gubbels CS , Spencer JB , Sanders RD , Land JA , Rubio‐Gozalbo E . Ovarian function in girls and women with GALT‐deficiency galactosemia. J Inherit Metab Dis. 2011;34:357‐366. 20978943 10.1007/s10545-010-9221-4 PMC3063539

[7] Nelson LM . Clinical practice. Primary ovarian insufficiency. N Engl J of Med. 2009;360:606‐614. 19196677 10.1056/NEJMcp0808697 PMC2762081

[8] Strauss III JF , Williams CJ . Ovarian life cycle. In Yen and Jaffe's Reprod Endocrinol. 2019;167 e169‐205.

[9] Liu K , Rajareddy S , Liu L , et al. Control of mammalian oocyte growth and early follicular development by the oocyte PI3 kinase pathway: new roles for an old timer. Develop Biol. 2206;299:1‐11. 10.1016/j.ydbio.2006.07.038 16970938

[10] Ford EA , Beckett EL , Roman SD , McLaughlin EA , Sutherland JM . Advances in human primordial follicle activation and premature ovarian insufficiency. Reproduction. 2020;159:R15‐R29. 31376814 10.1530/REP-19-0201

[11] Rubio‐Gozalbo ME , Gubbels CS , Bakker JA . Gonadal function in male and female patients with classic galactosemia. Hum Reprod Update. 2010;16:177‐188. 19793842 10.1093/humupd/dmp038

[12] Mamsen LS , Kelsey TW , Ernst E , Macklon KT , Lund AM , Andersen CY . Cryopreservation of ovarian tissue may be considered in young girls with galactosemia. J Assist Reprod Genet. 2018;35:1209‐1217. 29804175 10.1007/s10815-018-1209-2 PMC6063818

[13] Panay N , Anderson RA , Nappi RE , et al. Premature ovarian insufficiency: an International Menopause Society White Paper. Climacteric. 2020;23:426‐446. 32896176 10.1080/13697137.2020.1804547

[14] Badger T , Kastury R , Kavarthapu R , et al. Ovarian histology in children with classic galactosemia and correlation with endocrine and metabolic markers. Fertil Steril. 2024;16. S0015‐0282(24)00456‐4. 10.1016/j.fertnstert.2024.05.146 38761847

[15] Kushner RF , Ryan EL , Sefton JM , et al. A *Drosophila melanogaster* model of classic galactosemia. Dis Model Mech. 2010;3:618‐627. 20519569 10.1242/dmm.005041 PMC2931538

[16] Vanoevelen JM , van Erven B , Bierau J , et al. Impaired fertility and motor function in a zebrafish model for classic galactosemia. J Inherit Metab Dis. 2018;41:117‐127. 28913702 10.1007/s10545-017-0071-1 PMC5786655

[17] Hagen‐Lillevik S , Johnson J , Lai K . Early postnatal alterations in follicular stress response and survival in a mouse model of Classic Galactosemia. J Ovarian Res. 2022;15:122. doi: 10.1186/s13048-022-01049-2 36414970 PMC9682695

[18] Rasmussen SA , Daenzer JMI , MacWilliams JA , et al. A galactose‐1‐phosphate uridylyltransferase‐null rat model of classic galactosemia mimics relevant patient outcomes and reveals tissue‐specific and longitudinal differences in galactose metabolism. J Inherit Metab Dis. 2020;43:518‐528. 31845342 10.1002/jimd.12205 PMC7318568

[19] Kinnear HM , Tomaszewski CE , Chang AL , et al. The ovarian stroma as a new frontier. Reproduction. 2020;160:R25‐R39. 32716007 10.1530/REP-19-0501 PMC7453977

[20] Wu M , Tang W , Chen Y , et al. Spatiotemporal transcriptomic changes of human ovarian aging and the regulatory role of FOXP1. Nat Aging. 2024;4:527‐545. 38594460 10.1038/s43587-024-00607-1 PMC11031396

[21] Fan X , Bialecka M , Moustakas I , et al. Single‐cell reconstruction of follicular remodeling in the human adult ovary. Nat Commun. 2019;10:3164. 31320652 10.1038/s41467-019-11036-9 PMC6639403

[22] Wagner M , Yoshihara M , Douagi I , et al. Single‐cell analysis of human ovarian cortex identifies distinct cell populations but no oogonial stem cells. Nat Commun. 2020;11:1147. 32123174 10.1038/s41467-020-14936-3 PMC7052271

[23] Oslowski CM , Urano F . Measuring ER stress and the unfolded protein response using mammalian tissue culture system. Methods Enzymol. 2011;490:71‐92. 21266244 10.1016/B978-0-12-385114-7.00004-0 PMC3701721

[24] Bhattarai KR , Riaz TA , Kim HR , Chae HJ . The aftermath of the interplay between the endoplasmic reticulum stress response and redox signaling. Exp Mol Med. 2021;2:151‐167. 10.1038/s12276-021-00560-8 PMC8080639 33558590

[25] Shimura T . Mitochondrial signaling pathways associated with DNA damage responses. Int J Mol Sci. 2023;24:6128. 37047099 10.3390/ijms24076128 PMC10094106

[26] Yan F , Zhao Q , Li Y , et al. The role of oxidative stress in ovarian aging: a review. J Ovarian Res. 2022;15:100. 36050696 10.1186/s13048-022-01032-x PMC9434839

[27] Smits MAJ , Schomakers BV , van Weeghel M , et al. Human ovarian aging is characterized by oxidative damage and mitochondrial dysfunction. Hum Reprod. 2023;38:2208‐2220. 37671592 10.1093/humrep/dead177 PMC10628503

[28] Cao SS , Kaufman RJ . Endoplasmic reticulum stress and oxidative stress in cell fate decision and human disease. Antioxid Redox Signal. 2014;21:396‐413. doi:10.1089/ars.2014.5851 24702237 PMC4076992

[29] Cadet J , Davies KJA . Oxidative DNA damage & repair: an introduction. Free Radic Biol Med. 2017;107:2‐12. 28363603 10.1016/j.freeradbiomed.2017.03.030 PMC5510741

[30] Sasaki H , Hamatani T , Kamijo S , et al. Impact of oxidative stress on age‐associated decline in oocyte developmental competence. Front Endocrinol (Lausanne). 2019;10:811. 31824426 10.3389/fendo.2019.00811 PMC6882737

[31] Immediata V , Ronchetti C , Spadaro D , Cirillo F , Levi‐Setti PE . Oxidative stress and human ovarian response‐from somatic ovarian cells to oocytes damage: a clinical comprehensive narrative review. Antioxidants (Basel). 2022;11:1335. 35883826 10.3390/antiox11071335 PMC9311552

[32] Pintor J . Sugars, the crystalline lens and the development of cataracts. Biochem Pharmacol. 2012;1:1‐3.

[33] Umbayev B , Askarova S , Almabayeva A , Saliev T , Masoud AR , Bulanin D . Galactose‐induced skin aging: the role of oxidative stress. Oxid Med Cell Longev. 2020;2020:7145656. doi:10.1155/2020/7145656 32655772 PMC7317321

[34] Thakur M , Shaeib F , Khan SN , et al. Galactose and its metabolites deteriorate metaphase ii mouse oocyte quality and subsequent embryo development by disrupting the spindle structure. Sci Rep. 2017;7:231. doi:10.1038/s41598-017-00159-y 28331195 PMC5427935

[35] Banerjee S , Chakraborty P , Saha P , Bandyopadhyay SA , Banerjee S , Kabir SN . Ovotoxic effects of galactose involve attenuation of follicle‐stimulating hormone bioactivity and up‐regulation of granulosa cell p53 expression. PLoS One. 2012;7:e30709. 22319579 10.1371/journal.pone.0030709 PMC3271100

[36] Shi XY , Guan ZQ , Yu JN , Liu HL . Follicle stimulating hormone inhibits the expression of p53 up‐regulated modulator of apoptosis induced by reactive oxygen species through PI3K/Akt in mouse granulosa cells. Physiol Res. 2020;69:687‐694. 32584135 10.33549/physiolres.934421 PMC8549883

[37] Grosbois J , Demeestere I . Dynamics of PI3K and Hippo signaling pathways during in vitro human follicle activation. Hum Reprod. 2018;33:1705‐1714. 30032281 10.1093/humrep/dey250

[38] Maher JY , Islam MS , Yin O , et al. The role of Hippo pathway signaling and A‐kinase anchoring protein in primordial follicle activation and inhibition. F S Sci. 2022;3:118‐129. 35560009 10.1016/j.xfss.2022.03.002 PMC11096729

[39] Kawamura K , Cheng Y , Suzuki N , et al. Hippo signaling disruption and Akt stimulation of ovarian follicles for infertility treatment. Proc Natl Acad Sci USA. 2013;110:17474‐17479. 24082083 10.1073/pnas.1312830110 PMC3808580

[40] Zhao Y , Feng H , Zhang Y , et al. Current understandings of core pathways for the activation of mammalian primordial follicles. Cells. 2021;10:1491. 34199299 10.3390/cells10061491 PMC8231864

[41] De Felici M , Klinger FG . PI3K/PTEN/AKT signaling pathways in germ cell development and their involvement in germ cell tumors and ovarian dysfunctions. Int J Mol Sci. 2021;22:9838. 34575999 10.3390/ijms22189838 PMC8467417

[42] Lv X , He C , Huang C , et al. Timely expression and activation of YAP1 in granulosa cells is essential for ovarian follicle development. FASEB J. 2019;33:10049‐10064. 31199671 10.1096/fj.201900179RR PMC6704445

[43] Novella‐Maestre E , Herraiz S , Rodriguez‐Iglesias B , Diaz‐Garcia C , Pellicer A . Short‐term PTEN inhibition improves in vitro activation of primordial follicles, preserves follicular viability, and restores AMH levels in cryopreserved ovarian tissue from cancer patients. PLoS One. 2015;10:e0127786. 26024525 10.1371/journal.pone.0127786 PMC4449215

[44] Brown C , LaRocca J , Pietruska J , et al. Subfertility caused by altered follicular development and oocyte growth in female mice lacking PKB alpha/Akt1. Biol Reprod. 2010;82:246‐256. 19794155 10.1095/biolreprod.109.077925 PMC6058744

[45] John GB , Gallardo TD , Shirley LJ , Castrillon DH . Foxo3 is a PI3K‐dependent molecular switch controlling the initiation of oocyte growth. Dev Biol. 2008;321:197‐204. 18601916 10.1016/j.ydbio.2008.06.017 PMC2548299

[46] Shen M , Lin F , Zhang J , Tang Y , Chen WK , Liu H . Involvement of the up‐regulated FoxO1 expression in follicular granulosa cell apoptosis induced by oxidative stress. J Biol Chem. 2012;287:25727‐25740. 22669940 10.1074/jbc.M112.349902 PMC3406661

[47] Du XY , Huang J , Xu LQ , et al. The proto‐oncogene c‐src is involved in primordial follicle activation through the PI3K, PKC and MAPK signaling pathways. Reprod Biol Endocrinol. 2012;10:58. 22905678 10.1186/1477-7827-10-58 PMC3444437

[48] Jia CY , Li HH , Zhu XC , et al. MiR‐223 suppresses cell proliferation by targeting IGF‐1R. PLoS One. 2011;6:e27008. doi:10.1371/journal.pone.0027008 22073238 PMC3206888

[49] Quirk SM , Cowan RG , Harman RM , Hu CL , Porter DA . Ovarian follicular growth and atresia: the relationship between cell proliferation and survival. J Anim Sci. 2004;82:E‐Suppl:E40‐52. 10.2527/2004.8213_supplE40x 15471814

[50] Dhaunsi GS , Al‐Essa M . Downregulation of insulin‐like growth factor‐1 via nitric oxide production in a hypergalactosemic model of neonate skin fibroblast cultures. neonatology. 2016;110:225‐230. 27225493 10.1159/000446173

[51] Guahmich NL , Man L , Wang J , et al. Human theca arises from ovarian stroma and is comprised of three discrete subtypes. Commun Biol. 2023;6:7. 36599970 10.1038/s42003-022-04384-8 PMC9812973

[52] Zhou C , Guo Q , Lin J , et al. Single‐cell Atlas of Human ovaries reveals the role of the pyroptotic macrophage in ovarian aging. Adv Sci (Weinh). 2024;11:e2305175. 38036420 10.1002/advs.202305175 PMC10811476

[53] Irving‐Rodgers HF , Rodgers RJ . Extracellular matrix of the developing ovarian follicle. Semin Reprod Med. 2006;24:195‐203. 16944417 10.1055/s-2006-948549

[54] McIntush EW , Smith MF . Matrix metalloproteinases and tissue inhibitors of metalloproteinases in ovarian function. Rev Reprod. 1998;3:23‐30. 9509986 10.1530/ror.0.0030023

[55] Monniaux D , Huet‐Calderwood C , Le Bellego F , Fabre S , Monget P , Calderwood DA . Integrins in the ovary. Semin Reprod Med. 2006;24:251‐261. 16944422 10.1055/s-2006-948554

