# Supplementary material for: Single‐nucleus and spatial transcriptomics of paediatric ovary: Molecular insights into the dysregulated signalling pathways underlying premature ovarian insufficiency in classic galactosemia
Source: Clin Transl Med. 2024 Oct 23;14(10):e70043. doi: 10.1002/ctm2.70043 (PMC11812122; doi:10.1002/ctm2.70043)

Supplementary Figures S1 to S9

Figure S1: UMAP plot showing gene expression of signature markers specific to seven distinct major cell types identified.

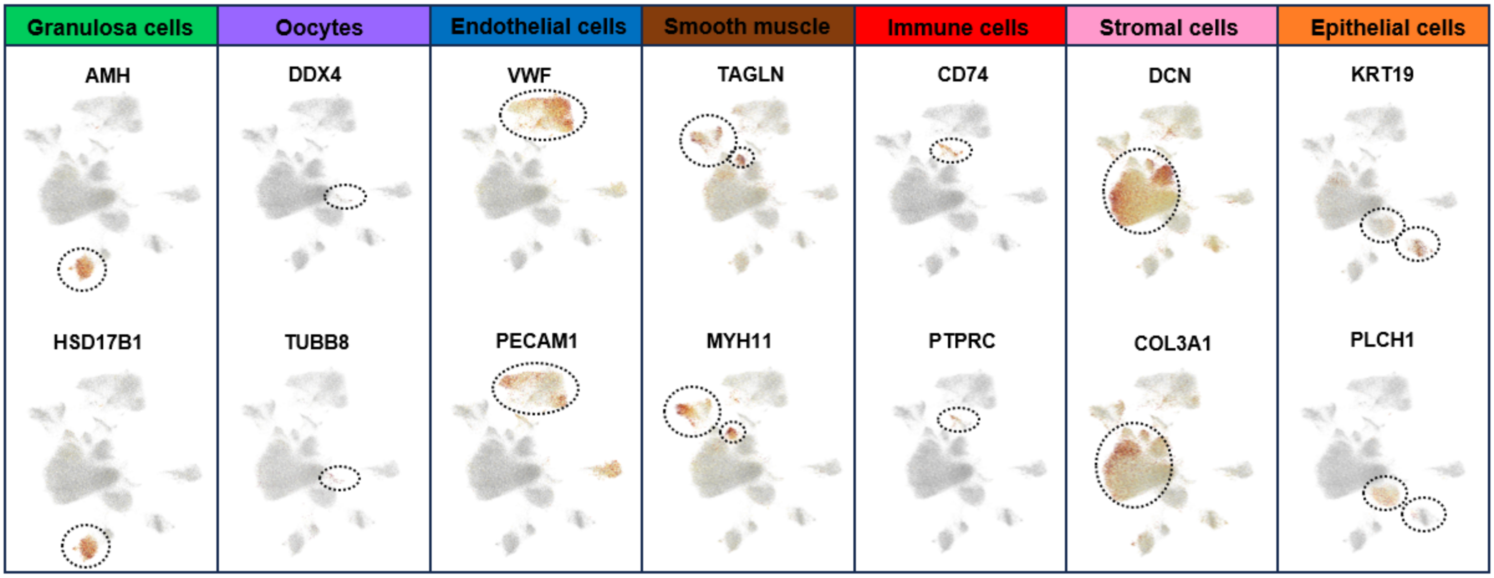

Figure S2: Bar plots of GO enrichment results for differentially expressed marker genes in seven distinct ovarian cell types.

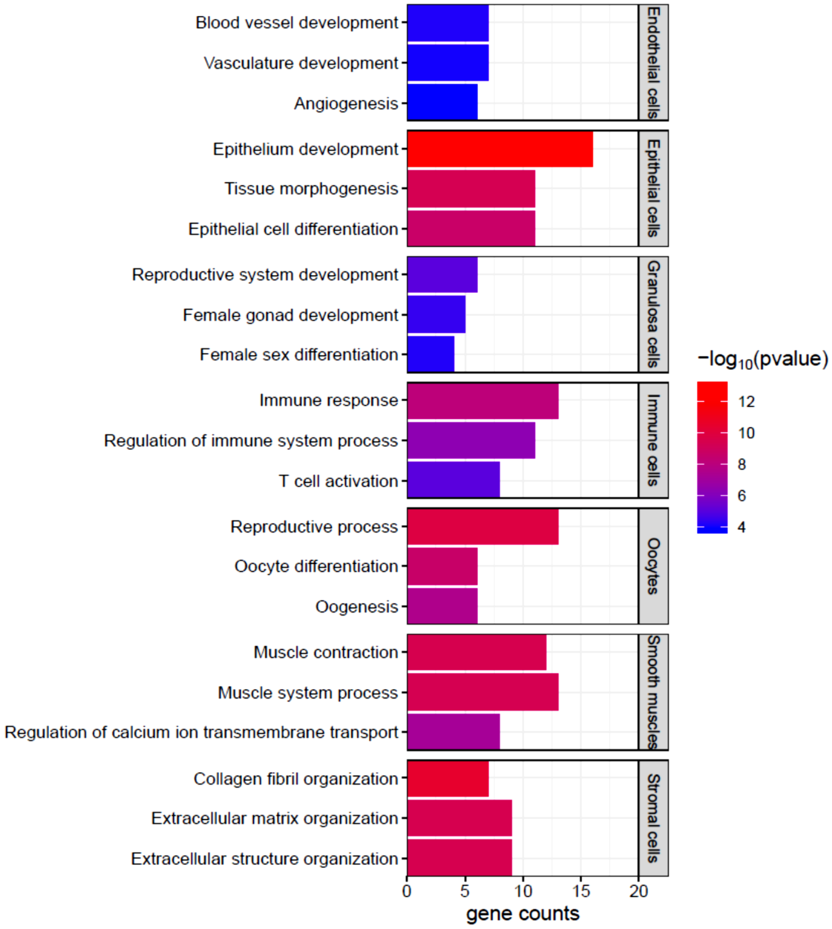

Figure S3: List of key genes participating in autphagy pathway that were upregulated in the granulosa cells of CG group

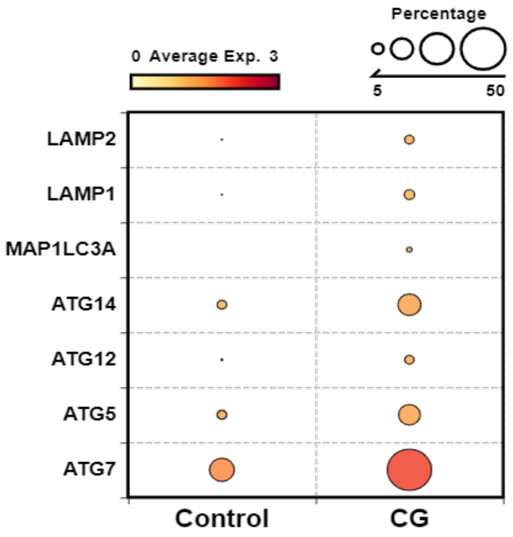

Figure S4: Causal network analysis: CHEK2 gene upregulated in the granulosa cells of CG patients is interacting with other downstream targets genes predicted to induce apoptosis of gonadal cells.

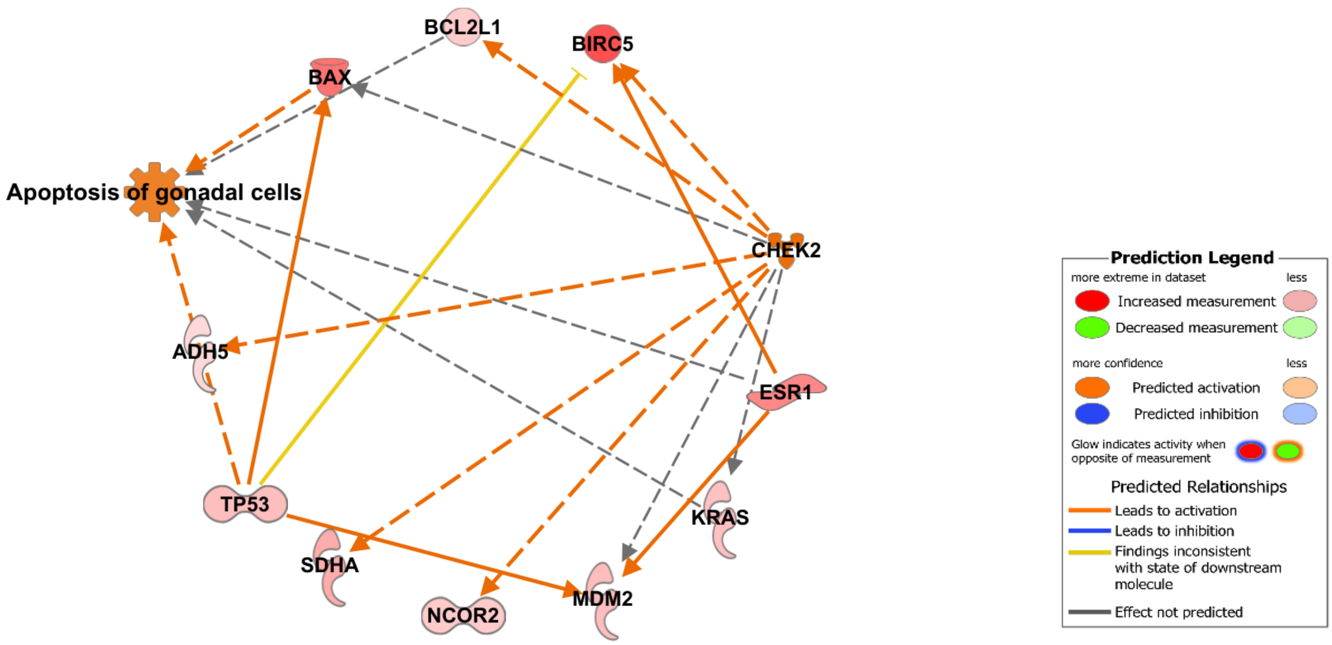

Figure S5: Causal network analysis: TP53 gene invloved in p53 signaling pathway is upregulated in the granulosa cells of CG patients, and is shown to be regulating apoptosis and senescence pathways genes.

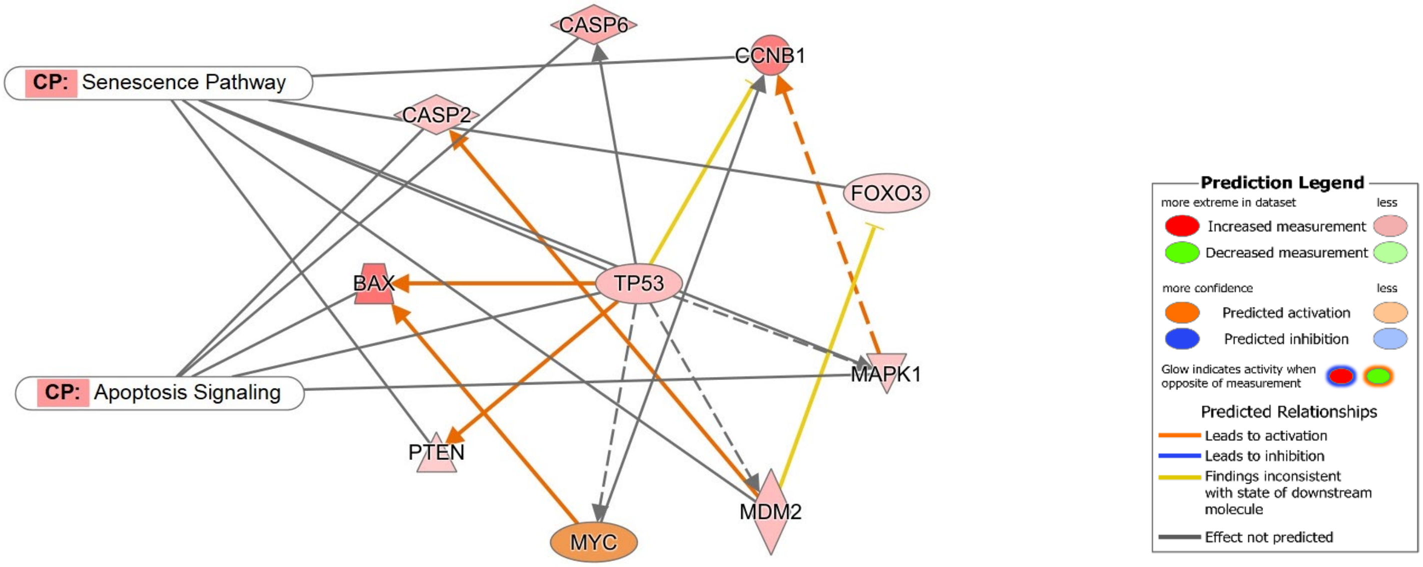

Figure S6: UMAP plot showing gene expression of signature markers specific to SC1, SC2, SC3 and SC4 subclusters.

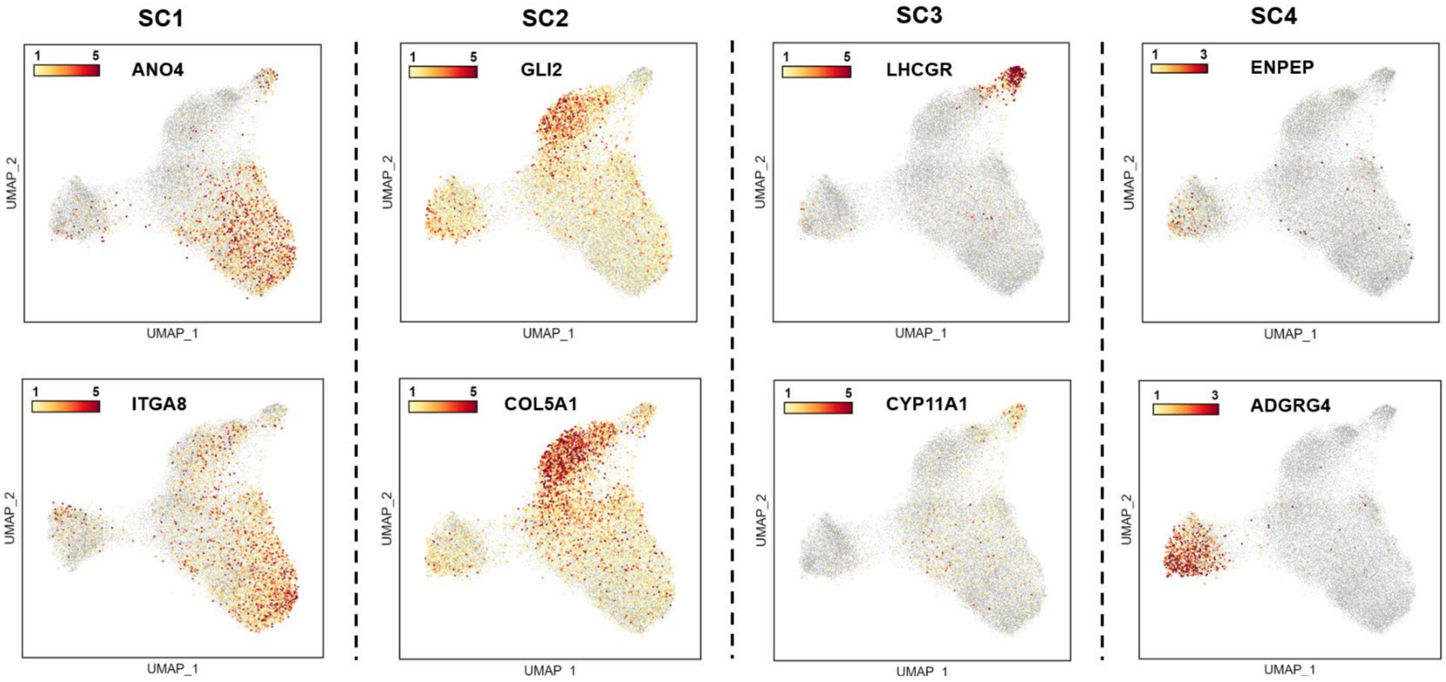

Figure S7: Network analysis showing crosstalk between PTEN/PI3K/AKT signaling genes with other signaling pathways such as ER-stress, apoptosis, autophagy, ATM and senescence.

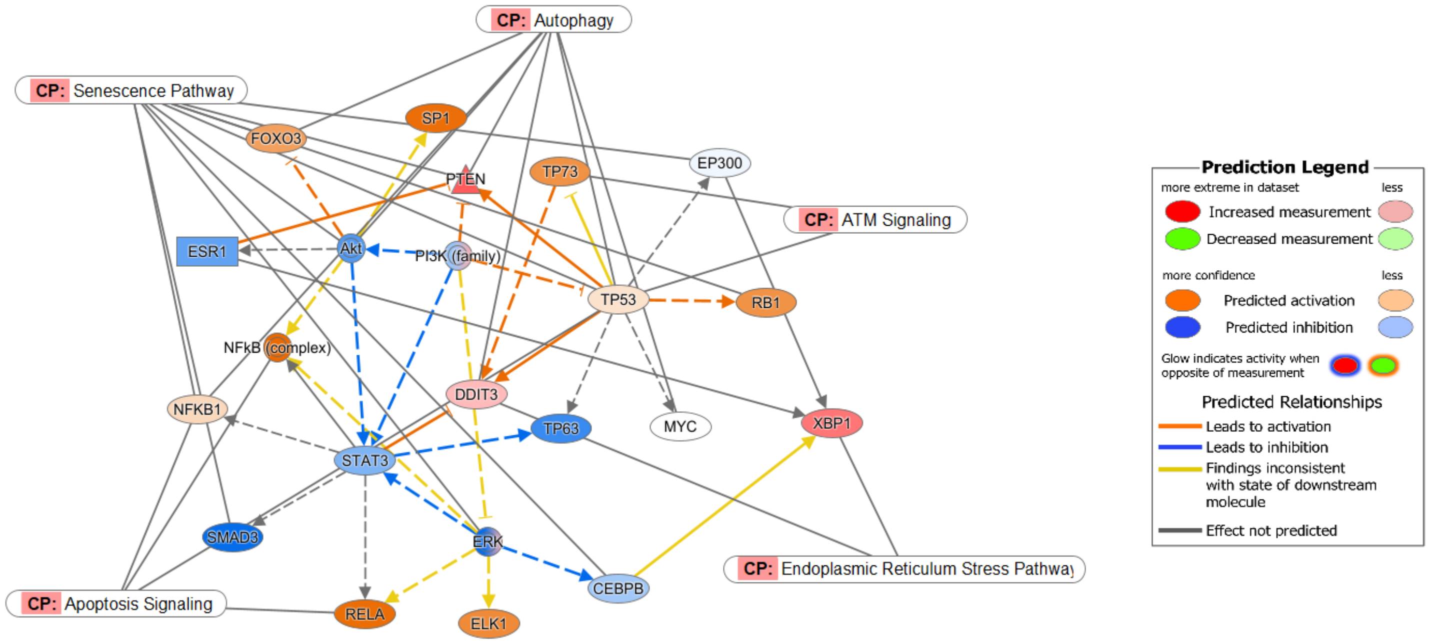

Figure S8: IHC analysis showing expression of p-H2A.X in the primordial follicles and TUNEL positive staining in the stromal cells of control and CG ovaries. DAB staining is shown as brown color. Scale bar is 50 μm.

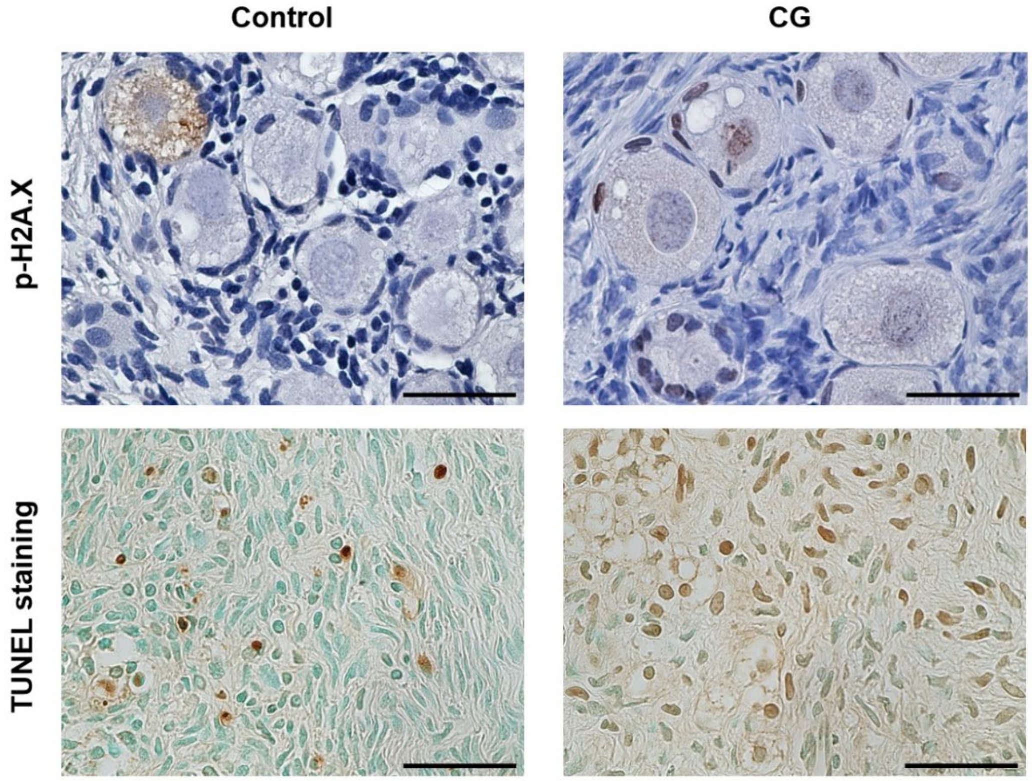

Figure S9: IHC analysis showing expression of cleaved-CASP3 and p-H2A.X in the granulosa cells of mouse ovaries that were treated with and without 5mM and 10mM D-galactose (D-Gal) for 48 h in culture medium. DAB staining is shown as brown color. Scale bar is 200 μm.

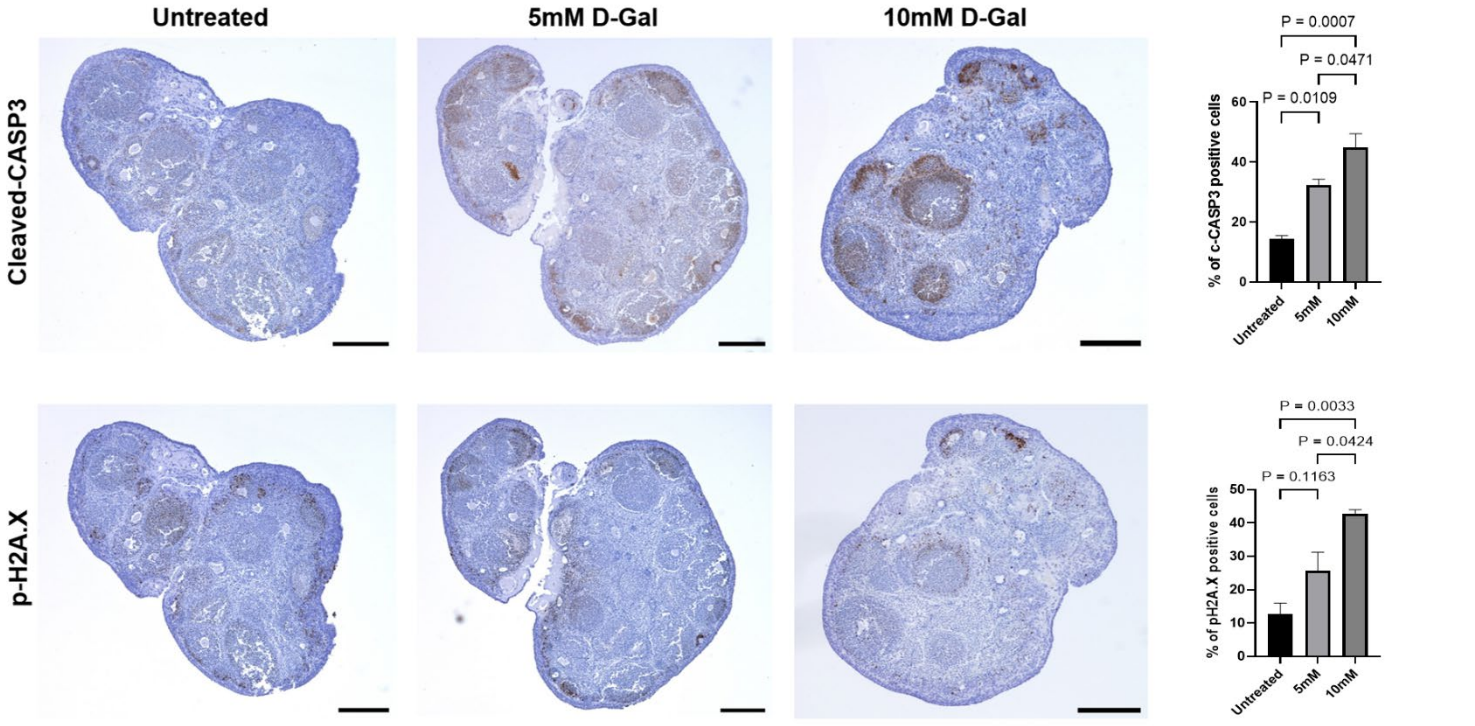

Supplement: Supplementary file 1 — Supporting information [file CTM2-14-e70043-s001.pdf]
