# Supplementary material for: Single‐nucleus and spatial transcriptomics of paediatric ovary: Molecular insights into the dysregulated signalling pathways underlying premature ovarian insufficiency in classic galactosemia
Source: Clin Transl Med. 2024 Oct 23;14(10):e70043. doi: 10.1002/ctm2.70043 (PMC11812122; doi:10.1002/ctm2.70043)
Supplement: Supplementary file 3 — Supporting information [file CTM2-14-e70043-s005.docx]

**Supplementary Table S2**

IPA Causal Network Analysis : Upstream regulator genes identified in THE DEGs from granulosa cell cluster [SnRNAseq dataset: CG vs control]

| **Upstream Regulator** | **Molecule Type** | **Predicted Activation State** | **Target Molecules in Dataset** | |  |  |  |  |  |  |  |
| --- | --- | --- | --- | --- | --- | --- | --- | --- | --- | --- | --- |
| AKR1C1/AKR1C2 | enzyme | Activated | GFPT1,GRIK2,HIF1A,HK1,LDHA,LDHB | | |  |  |  |  |  |  |
| AKT1 | kinase |  | CTNNB1,DIAPH1,DIAPH2,DIAPH3,FOXO1,GCLC,GSR,GSS,ITGB1,SLC4A7 | | | | | | |  |  |
| ALKBH1 | enzyme | Activated | METTL3,MT-ATP6,MT-CO1,MT-CO2,MT-CYB,MT-ND2,MT-ND4,MT-ND4L,MT-ND5 | | | | | | |  |  |
| ALKBH7 | other | Activated | MT-ATP6,MT-CO1,MT-CO2,MT-CYB,MT-ND1,MT-ND2,MT-ND3,MT-ND4,MT-ND5,MT-ND6 | | | | | | | |  |
| ALOX5 | enzyme | Activated | AMN1,APOBEC3C,APOBEC3F,APOBEC3G,ATF4,ATP11C,BST2,CDH6,CDKL1,CFI | | | | | | |  |  |
| AMFR | transmembrane receptor | Activated | ACAT2,FDFT1,FDPS,HMGCR,HMGCS1,IDI1,LSS,MSMO1,SQLE,TM7SF2 | | | | | |  |  |  |
| ANXA2 | other | Inhibited | BAX,MDM2,PPM1D,PTEN,SESN1,TP53,TP53I3 | | | |  |  |  |  |  |
| APOC3 | transporter | Activated | APOA1,CLPX,HSPA9,HSPD1,IL6ST,LONP1,SOD2,TGFB1 | | | | |  |  |  |  |
| AR | ligand-dependent nuclear receptor | Activated | ABCA1,ABCC4,ACTR3,ADGRA3,AMBRA1,AR,ARHGEF7,BCL2,BCL2L1,BIRC5 | | | | | | |  |  |
| AREG | growth factor | Activated | ARHGAP11A,AURKB,BCL2,BIRC5,BSG,CCNB1,CCNF,CDC45,CENPF,DESI2 | | | | | | |  |  |
| ARID1A | transcription regulator |  | ABI2,ACTA2,AGL,ANKH,ARAP1,ARID5B,ARMH3,ATG7,ATP5F1D,ATP5PF | | | | | |  |  |  |
| ARL4D | enzyme | Activated | AURKB,BUB1B,CDK1,DUT,GINS2,RRM1 | | |  |  |  |  |  |  |
| ATF3 | transcription regulator | Activated | ARF1,ATF4,AURKB,CAD,CDK1,CTNNB1,CTPS1,DUT,GSN,HSPA5 | | | | | |  |  |  |
| ATF6 | transcription regulator | Activated | ATP2A2,BAX,BCL2,BUB1,C8orf44-SGK3/SGK3,DAPK1,FDPS,HMMR,HSP90B1,HSPA5 | | | | | | | |  |
| ATG7 | enzyme |  | ACTA2,AIFM1,AKAP11,CD44,CDH2,COL1A1,COL1A2,FN1,IRS1,LAMP1 | | | | | |  |  |  |
| ATM | kinase | Activated | ATF4,ATF6,ATR, BAX,BID,BIRC2,CASP3,CHEK1,CHEK2,CREB1,DAXX, MAPK1,MAPK2,PARP1,PPP2R2C,SAPK,TP53,  TP53I3,TRAF2 | | | | | | | | |
| BACH1 | transcription regulator | Inhibited | ABCB7,ATP5F1B,ATP5F1D,ATP5PF,ATP5PO,CALM1 (includes others),COX15,DNAJC7,EWSR1,FTH1 | | | | | | | | |
| BANF1 | other | Inhibited | ADAR,BST2,DDX60,EIF2AK2,HERC5,HLA-A,IFI27,IFI44,IFI6,IFITM2 | | | | | |  |  |  |
| BARX2 | transcription regulator | Activated | ANLN,DMXL1,DYNLL1,ESR1,FGF12,FLNA | | | |  |  |  |  |  |
| BCR (complex) | complex | Activated | AIMP2,ATP6V0A1,ATP6V0A2,ATP6V0B,ATP6V1C1,ATP6V1D,ATP6V1E1,ATP6V1H,BCL2L1,CBX3 | | | | | | | | |
| BIRC3 | enzyme |  | BCL2,BCL2L1,REL,XIAP | |  |  |  |  |  |  |  |
| BIRC5 | other |  | BCL2L1,BIRC5,CHUK,MDM2,TP53,UVRAG | | | |  |  |  |  |  |
| BMPR2 | kinase | Activated | CLIC4,GATA6,GTSE1,INHBA,JUP,KIF5B,LEPROT,MAP2K1,MAPK1,MSN | | | | | |  |  |  |
| BPIFB1 | other | Inhibited | CCNC,CDH2,CDK7,CDK8,MAP2K1,MAP3K1,MAP3K7,MAP4K5,MAPK9 | | | | | |  |  |  |
| BRCA1 | transcription regulator |  | ACTB,ATM,BACH1,BAX,BIRC5,BRCA1,CCNB1,CDH3,CTNNB1,CYP19A1 | | | | | |  |  |  |
| CA9 | enzyme |  | ATP5F1A,ATP5F1B,ATP5F1C,ATP5F1D,ATP5MC2,ATP5ME,ATP5MG,ATP5PF,ATP5PO,COX10 | | | | | | | |  |
| CAB39L | kinase | Activated | ATP5F1E,ATP5MC2,ATP5ME,ATP5MF,ATP5MG,ATP6V0E1,ATP6V1E1,COX4I1,COX5B,COX6C | | | | | | | |  |
| CAPN6 | peptidase | Activated | ACTG1,AMH,BIRC2,BIRC5,BMPR1A,BMPR2,BTRC,CSNK1D,CSNK1E,CTNNA1 | | | | | | |  |  |
| CAVIN3 | other |  | BRCA1,BRCC3,MDC1,UBE4A | |  |  |  |  |  |  |  |
| CCAR2 | peptidase | Activated | ATF2,CAMK2G,CNN3,CREB1,DDB2,KIF1B,MDM2,MED15,RAD50,SKI | | | | | |  |  |  |
| CCAT1 | other |  | CDH2,EGFR,HMGA2,VIM | |  |  |  |  |  |  |  |
| CCN5 | growth factor |  | CD44,ERBB2,ESR1,FN1,HIF1A,IGFBP7,JUP,SMAD3,SPARC,TGFB1 | | | | | |  |  |  |
| CCND1 | transcription regulator | Inhibited | AFAP1,AHI1,AR,ATP6V0E1,BCL2,BCLAF1,BRWD1,C7,CALU,CBFA2T2 | | | | | |  |  |  |
| CCNK | kinase | Activated | ATR,BRCA1,BRCC3,COPS6,FANCD2,FANCI,GABPB2,MDC1,ORC3,POLA2 | | | | | | |  |  |
| CD24 | other | Activated | ADD3,ASPM,ATP13A3,BCLAF1,CDC27,CTTN,DEPDC1B,DIAPH2,DLG1,DNAJC13 | | | | | | |  |  |
| CD44 | other | Activated | AAK1,ABCA5,ACTA2,ADPGK,BCL2L1,BIRC2,BIRC5,CCNG1,CD44,CTTN | | | | | |  |  |  |
| CD3E | transmembrane receptor | Activated | BCL2L1,BLM,BRCA1,CCND2,CDC25C,CDC45,CDC7,CHEK1,CLSPN,DNA2 | | | | | | |  |  |
| CDH1 | other |  | ACTB,BIRC2,BIRC5,CDC42,CDH2,CTNNB1,DDB1,EGFR,ERBB2,FN1 | | | | | |  |  |  |
| CDK4 | kinase |  | ATP6V0E1,BRWD1,C7,CBFA2T2,CDC45,CDCA2,CDCA7,CENPF,CENPK,CEP55 | | | | | | |  |  |
| CDK6 | kinase |  | ACACA,APBB2,ATP5ME,ATP5PF,AUH,BNIP3L,CACNB4,DGKD,DGKH,FN1 | | | | | | |  |  |
| CDK12 | kinase | Activated | ATR,BRCA1,FANCD2,FANCI,SMARCC2 | | |  |  |  |  |  |  |
| CDK4/6 (family) | group | Inhibited | AFAP1,AHI1,BCLAF1,CALU,CDC45,DYNC1LI1,EEF1B2,EIF5,EMSY,ENO1 | | | | | | |  |  |
| CDKN1A | kinase | Inhibited | APP,BAX,BCL2,BCL2L1,BIRC5,BRCA1,CCNB1,CD44,CDC25C,CDK1 | | | | | |  |  |  |
| CELF2 | other |  | BAX,BCL2,SIRT1,TP53 | |  |  |  |  |  |  |  |
| CG (complex) | complex | Activated | ABCD3,ACSS2,ADAMTS9,ALAD,ANK2,APPL2,AR,ARHGAP6,ARID5B,ATF4 | | | | | | |  |  |
| CHEK2 | kinase | Activated | ADH5,BAX,BCL2L1,BIRC5,KRAS,MDM2,NCOR2,SDHA,TP53BP2 | | | | | |  |  |  |
| CK2 ALPHA (family) | group | Activated | BCL2L1,BIRC2,BIRC5,CTNNB1,MGMT | | |  |  |  |  |  |  |
| CKAP2L | other | Activated | AURKB,BIRC5,BUB1,CCNB1,CDK1,CENPE,CENPF,CENPP,CKAP5,ESPL1 | | | | | | |  |  |
| CLOCK | transcription regulator |  | ANKRD12,APP,ARHGEF10,BLTP2,CBFB,CEMIP,CLIP1,CNIH1,COMMD2,CPD | | | | | | |  |  |
| CLPB | transcription regulator | Inhibited | ACO2,CS,DLST,NDUFA10,NDUFA11,NDUFA12,NDUFA13,NDUFA7,NDUFA9,NDUFB8 | | | | | | | |  |
| CNOT7 | transcription regulator |  | B2M,BST2,HERC6,IFI27,IFI35,IFI44L,IFI6,LGALS3BP,PARP12,PLSCR1 | | | | | |  |  |  |
| CNTNAP4 | other | Activated | CREBBP,GSK3B,MAP2K6,MAP3K4,MAPK1IP1L,MTOR,PIK3CB | | | | |  |  |  |  |
| COPS5 | transcription regulator | Activated | AMD1,ANP32E,APAF1,ATRX,AURKB,BAX,BIRC5,BLCAP,BUB1,CBX1 | | | | | |  |  |  |
| CST5 | other | Inhibited | ABCC1,ABLIM1,ACAT2,ADSL,AHCTF1,ANXA2,ANXA6,AP3B1,AP3D1,ARHGAP12 | | | | | | |  |  |
| CTSB | peptidase | Activated | ACTA2,CTSB,PRKDC,XRCC5,XRCC6 | | |  |  |  |  |  |  |
| CUL3 | enzyme | Inhibited | BRD2,BRD3,BRD4,KATNA1,SETD2 | | |  |  |  |  |  |  |
| CUL7 | enzyme |  | BIRC5,CDH2,TP53,VIM,ZEB1 | |  |  |  |  |  |  |  |
| CUL4B | other | Inhibited | CCNH,DARS1,GLO1,HSPA9,NDUFB1,NME1,PIK3CA,PRDX3,PSMA1,SOD1 | | | | | | |  |  |
| CXCL12 | cytokine |  | ACTA2,ANKRD12,BAX,BCL2,BCL2L1,CANX,CCNT1,CD44,CELF1,CTNNB1 | | | | | | |  |  |
| CYB561A3 | enzyme |  | ABCA1,ACTG2,AP1B1,ATF5,COTL1,FTH1,FTL,IREB2,TTC7B | | | | |  |  |  |  |
| CYGB | transporter |  | CD74,FBLN1,LONRF1,MDK,WIPF3,ZNF486,ZNF736 | | | |  |  |  |  |  |
| CYTOR | other |  | CDH2,CREB1,MAPK14,SIRT3,SOD1,SOD2,STAT1,STAT3,VIM,ZEB1 | | | | | |  |  |  |
| DANCR | other |  | CDH2,CTNNB1,LIPG,RAB1A,RUNX2,TIMP2,VIM,ZEB1 | | | | |  |  |  |  |
| DAP3 | other | Activated | MT-ATP6,MT-CO1,MT-CO2,MT-CO3,MT-CYB,MT-ND1,MT-ND2,MT-ND3,MT-ND4,MT-ND4L | | | | | | | |  |
| DCK | kinase |  | DNMT1,DNMT3A,TET1 | |  |  |  |  |  |  |  |
| DDX5 | enzyme | Activated | ACTA2,ATP5F1C,ATP5MC2,ATP5ME,ATP5MG,ATP5PF,BAX,BCL2,COX5A,COX5B | | | | | | |  |  |
| DDX3X | enzyme | Activated | AR,CTNNB1,EIF2A,EZH2,MAP3K7,MAPK14,PRKRA,RAC1,RPL11,RPL13 | | | | | |  |  |  |
| DHPS | enzyme | Activated | ATP5F1A,MT-CO1,NDUFB8,SDHB | | |  |  |  |  |  |  |
| DLEU2 | other |  | ACTA2,PRKACB,RAP1B,VIM | |  |  |  |  |  |  |  |
| DLX1 | transcription regulator | Activated | ASPM,BCL2L1,CAP2,CD44,CDH6,COPA,DAB2,FBLN1,FGF13,FGFR1 | | | | | |  |  |  |
| DNAJB11 | other |  | ATF4,EIF2AK3,HSPA5 | |  |  |  |  |  |  |  |
| DNM3OS | other | Activated | ATM,CHEK2,MRE11,NBN,PRKDC,RAD50,TP53,XRCC5 | | | | |  |  |  |  |
| DNMT1 | enzyme | Inhibited | ABCC5,BIRC5,BRD8,CCDC14,CCNB1,CDC25C,CDH2,CDK1,CDK11A,CEP152 | | | | | | |  |  |
| DOT1L | phosphatase |  | PABPC1,PPIA,RPL19,RPL41,RPLP0,RPS2,RPS23,TUBA1C,UBB | | | | | |  |  |  |
| DPH5 | enzyme |  | CREB1,CREBBP,EEF2,EP300,SMAD3,SP1,STAT1,STAT3,TP53 | | | | | |  |  |  |
| DUB (family) | group |  | ABCA1,LDLR,MYLIP | |  |  |  |  |  |  |  |
| E2F1 | transcription regulator | Activated | ALG5,APAF1,AR,ARID1A,ATG12,ATG14,ATG4B,ATG7,AURKB,BCL2 | | | | | |  |  |  |
| E2F3 | transcription regulator |  | ARPC1A,CCNB1,CDK1,CTNND1,DAG1,ECE1,EEIG1,FST,HMGB2,HSPD1 | | | | | |  |  |  |
| E2F4 | transcription regulator |  | ANLN,ATRX,AURKB,BARD1,BRCA1,BRD2,BUB3,CALM1 (includes others),CBX5,CDC25C | | | | | | | |  |
| E2F6 | transcription regulator | Inhibited | BRCA1,CAMTA2,CBX5,CDC45,CDC7,CHEK2,DCTN4,DDX11,FNTB,GINS2 | | | | | | |  |  |
| E2F (family) | group | Activated | ALG5,CBX5,CDC25C,CDC45,CDC7,CDK1,CENPO,CSDE1,DDX11,DLEU1 | | | | | | |  |  |
| EGLN2 | enzyme | Inhibited | ANKRD13C,AP2A1,ARHGEF10,CHD8,CLASP2,CLOCK,DYNC1LI2,GCC2,GNS,HIF1A | | | | | | |  |  |
| EGR2 | transcription regulator | Activated | ACAT2,FDPS,HMGCR,HMGCS1,IDI1,IGF2BP2,MVD | | | |  |  |  |  |  |
| EIF3A | translation regulator | Inhibited | RAD23B,RPA1,RPA2,RPA3,RPTOR,XPA,XPC | | | |  |  |  |  |  |
| EIF4E | translation regulator | Activated | BCL2L1,BIRC5,ESR1,FOXM1,MDM2,PRPF6,PRPF8,SNRNP200,TP53,TP53BP1 | | | | | | |  |  |
| EIF4G1 | translation regulator | Activated | ATM,ATRX,BCL2L1,BIRC5,BRCA1,BRCA2,CHEK1,CTNND1,HIF1A,MRE11 | | | | | |  |  |  |
| ELAVL1 | other | Activated | ABCA1,ANXA11,APPL2,ATG12,ATG5,AXL,BCL2L1,CALM1 (includes others),CASP2,CDH2 | | | | | | | |  |
| ELF5 | transcription regulator | Inhibited | CCNB1,ESR1,ILK,ITGB1,MEIS2,PGR,RUNX1 | | | |  |  |  |  |  |
| EN1 | transcription regulator |  | ACTA2,COL1A1,COL1A2 | |  |  |  |  |  |  |  |
| EOMES | transcription regulator | Inhibited | ACTA2,COL3A1,COL6A3,PMP22 | | |  |  |  |  |  |  |
| ERBB2 | kinase | Activated | ABL1,ANGPT2,ANXA2,APLP2,AR,ASPM,BARD1,BCL2,BCL2L1,BIRC5 | | | | | |  |  |  |
| ERG | transcription regulator | Activated | ABCA1,ACOT8,ADD1,AHCYL2,AKAP8L,AR,ARFGEF2,ARHGAP17,ARHGAP24,ARHGAP31 | | | | | | | |  |
| ESR1 | ligand-dependent nuclear receptor |  | AASS,ABCC5,ABHD2,ABLIM1,ACTA2,ADAM17,ANO10,ARHGAP1,ASPM,ATG13 | | | | | | |  |  |
| ESR2 | ligand-dependent nuclear receptor |  | ABCC4,ABHD2,ACSL3,ANKH,APC,APPBP2,AR,ARHGEF7,ARID5B,B4GALT1 | | | | | | |  |  |
| ESRP1 | other |  | CD44,CTNND1,EXOC7,RAC1 | |  |  |  |  |  |  |  |
| ETS1 | transcription regulator | Activated | ARPC2,B4GALT5,BACH1,BCL11A,BCL2L1,BTRC,CCDC90B,CD44,CDH2,CDK11A | | | | | | |  |  |
| ETV3 | transcription regulator | Inhibited | BST2,DDX60,DDX60L,EIF2AK2,GTPBP2,HERC5,IFI35,IFI44,IFI44L,IFI6 | | | | | |  |  |  |
| ETV6 | transcription regulator | Inhibited | ACTA2,CMTR1,DDX60,DDX60L,EIF2AK2,IFI35,IFI44,IFI44L,LGALS3BP,MX1 | | | | | | |  |  |
| F3 | transmembrane receptor | Inhibited | ADAR,BST2,CD47,DDX60,EIF2AK2,EPSTI1,GMPR,HERC6,IFI35,IFI44 | | | | | |  |  |  |
| FAM117B | other | Activated | ABCC1,ABCC5,CAT,GCLC,GSR,GSTA4,IDH1,ME1,PGD,PRDX1 | | | | | |  |  |  |
| FAM120A | other | Activated | ACLY,ACSS2,CDH2,FDFT1,HMGCS1,MVD,SCD,SP1 | | | | |  |  |  |  |
| FBN1 | other | Inhibited | COL1A2,COL3A1,COL4A2,COL6A3,FBN1,LTBP1,LTBP3 | | | | |  |  |  |  |
| FBP1 | phosphatase |  | BAX,BCL2,MT-CO2,TP53 | |  |  |  |  |  |  |  |
| FBXW7 | enzyme | Inhibited | ADAM17,ATP5F1A,CS,DDX11,DLST,DNAJA1,FOXRED1,HMGB1,HSPA8,HSPE1 | | | | | | |  |  |
| FGF7 | growth factor | Activated | ADSL,ADSS2,CAD,HPRT1,IL7,IRF2,SCD,STAT1 | | | |  |  |  |  |  |
| FGFR1 | kinase |  | BAX,BCLAF1,CDC5L,CPT1A,ERCC6L2,FLNA,G3BP1,KIAA0586,KIF14,LINC00472 | | | | | | |  |  |
| FGFR4 | kinase | Inhibited | ACO1,ANKRD52,ATP9A,CRK,CYRIB,DAP,EIF3B,ERCC5,ESYT2,FGFR1 | | | | | |  |  |  |
| FLCN | other | Inhibited | ATP5PF,ATP6V0E1,ATP6V1C1,ENO1,LDHA,PPARGC1B,VEGFA | | | | | |  |  |  |
| FOXM1 | transcription regulator | Activated | ASPM,ATF2,AURKB,BIRC5,BRCA2,BUB1B,CCNB1,CCND2,CD276,CDC25C | | | | | | |  |  |
| FSH (complex) | complex |  | ACTA2,ACTB,ACTG2,ACTR2,ADAMTS16,ADCY9,AKAP7,ALDH3A2,ALPL,AMH | | | | | | |  |  |
| FUS | transcription regulator | Activated | ATF5,CALM1 (includes others),CNOT1,EIF4G2,FLNA,GINS4,HSPA5,HSPA9,HSPD1,JPT1 | | | | | | | |  |
| FZD8 | G-protein coupled receptor |  | ACTA2,COL1A1,FN1,TP53,VCAN,VIM | | |  |  |  |  |  |  |
| GFI1B | transcription regulator |  | ARNT,BCL2L1,CSDE1,ELF4,GABPB2,HADH,ITSN1 | | | |  |  |  |  |  |
| GFM2 | translation regulator | Activated | MT-CO2,MT-ND2,MT-ND5,MT-ND6 | | |  |  |  |  |  |  |
| GLS | enzyme |  | ACTA2,COL1A1,FN1,HIF1A | |  |  |  |  |  |  |  |
| GNA12 | enzyme | Activated | ARHGEF1,ARHGEF12,ARHGEF7,ARPC5,DDR2,EGFR,EZR,GNA13,IQGAP1,IRS1 | | | | | | |  |  |
| GNL1 | other |  | BAX,BCL2,BCL2L1,BID,BIRC5,TP53 | | |  |  |  |  |  |  |
| GSK3B | kinase | Activated | BAX,BCL2,BCL2L1,BIRC5,CDC25C,CDH11,CFLAR,CTNNB1,GSK3B,HIF1A | | | | | | |  |  |
| GSTA4 | enzyme |  | CCNB1,CDC25C,CDK1 | |  |  |  |  |  |  |  |
| H2AZ1 | other | Activated | AURKB,BIRC5,CEP55,CKAP2L,DLGAP5,FOXM1,GALNTL6,GINS2,MCM7,MKI67 | | | | | | |  |  |
| HAS1 | enzyme |  | CCNB1,CD44,CDC25C,CDK1 | |  |  |  |  |  |  |  |
| HAS2 | enzyme |  | ACTA2,CD44,CDH2,PTK2,TIMP1,VEGFA,VIM | | | |  |  |  |  |  |
| HAX1 | other | Inhibited | ACO2,CS,DLST,NDUFA13,NDUFA9,NDUFB9,NDUFC2,NDUFS2,NDUFS4,NNT | | | | | | |  |  |
| HELLS | enzyme | Activated | CCNB1,HSPD1,KMT2A,KRAS,MCM4,PDS5A,RALGPS2,RBL1,RNF146,SLC44A1 | | | | | | |  |  |
| HGF | growth factor |  | AIMP2,ARPC5,ATM,BCL2L1,BIRC2,CD44,CENPF,CHD2,CNBP,COPB1 | | | | | |  |  |  |
| HIBCH | enzyme |  | ABAT,ACOX2,ACSS2,ATF4,BCAT1,CPT1A,CYB5R3,GALT,GMDS,HADH | | | | | |  |  |  |
| HINT1 | enzyme |  | BAX,BCL2,TP53 |  |  |  |  |  |  |  |  |
| HISTONE H3 (family) | group |  | ADD3,ARHGEF17,ATF7IP,B2M,BAX,BCL2,CCND2,CDK14,CEACAM1,CHFR | | | | | | |  |  |
| HK2 | kinase | Inhibited | BCL2L1,MT-CO2,NDUFB8,SDHB,UQCRC2 | | | |  |  |  |  |  |
| HNF1A-AS1 | other | Activated | BIRC5,CDH2,CEP55,CTNNB1,ENO1,HJURP,HMGB2,JADE1,KIF22,KIF2C | | | | | |  |  |  |
| HNRNPA1 | other |  | ANXA7,CD44,EIF2AK2,ILK,KRAS,PKM,ZC3HAV1 | | | |  |  |  |  |  |
| HNRNPA2B1 | other |  | ADGRG2,ANXA7,ATG12,ATRX,BCL2L1,CACNB2,CEMIP,CUX1,DCLK1,DSE | | | | | | |  |  |
| HSF1 | transcription regulator |  | AMZ1,ANXA11,ATAD2,ATG7,BAX,BCL2,BCL2L1,BST2,CBX3,CCT3 | | | | | |  |  |  |
| HSP90AA1 | enzyme |  | CFLAR,FN1,GJA1,GRK3,SP1 | |  |  |  |  |  |  |  |
| HSP90AB1 | enzyme |  | ABCC1,CFLAR,HIF1A,SP1,STAT3 | | |  |  |  |  |  |  |
| HSP90B1 | other | Activated | ARF1,ARF3,CDC42,ESR1,HSP90B1,RAB10,RAB2A,RAB8B,RPL12,RPL27A | | | | | | |  |  |
| HSPA5 | enzyme | Activated | ACADM,ACADVL,ACLY,APP,ATF6,BCL2L1,COL1A2,CPT1A,EGFR,EIF2A | | | | | |  |  |  |
| HULC | other |  | ATG7,HMGA2,ITGB1,LAMP1,SQSTM1,VIM,ZEB1 | | | |  |  |  |  |  |
| ICMT-DT | other |  | BAX,BCL2,FSCN1,TP53 | |  |  |  |  |  |  |  |
| ID3 | transcription regulator | Activated | BRCA1,BRCA2,EXO1,FANCL,FANCM,MDC1,POLQ,PPARG,RBBP8,RFC3 | | | | | |  |  |  |
| IDH2 | enzyme |  | CTPS1,FN1,HIF1A,LDHA,PKM,TIGAR,TKT,VIM,ZEB1,ZEB2 | | | | |  |  |  |  |
| IFNA2 | cytokine | Activated | ADAM17,BAX,BIRC5,BST2,DDX60,EIF2AK2,HERC5,HERC6,HIF1A,HMGCS1 | | | | | | |  |  |
| IFNAR1 | transmembrane receptor | Activated | ADAR,BST2,CD47,DDX60,EIF2AK2,EPSTI1,GMPR,HERC6,IFI35,IFI44 | | | | | |  |  |  |
| IFNL1 | cytokine | Activated | BST2,DDX60,DDX60L,EIF2AK2,HERC5,HERC6,HLA-B,HLA-C,IFI27,IFI35 | | | | | |  |  |  |
| IFT88 | other | Inhibited | ACAT1,ACAT2,ACLY,CYP51A1,FDPS,HMGCR,IDI1,MVD | | | | |  |  |  |  |
| IGFBP2 | other | Activated | BCL2L1,CHD9,DDAH1,EGFR,ELOVL6,EMP2,ESCO2,ESR1,MALAT1,NEDD9 | | | | | | |  |  |
| IKZF1 | transcription regulator | Inhibited | ANKFY1,ANTXR1,ARF3,ARHGEF11,B2M,BCL2,BRCA1,BRCA2,DIPK1A,DNM3 | | | | | | |  |  |
| IL15 | cytokine | Activated | AKT2,ARNT,ATM,BCL2,BCL2L1,CAMK2G,CCND2,CHRNA7,CREBBP,EIF4E | | | | | | |  |  |
| IL31 | other | Inhibited | CCNB1,CDK1,MCM4,RB1 | |  |  |  |  |  |  |  |
| ING1 | transcription regulator | Activated | DGCR8,EPS15,ITSN2,JAK2,TLE5,TP53 | | |  |  |  |  |  |  |
| INHBA | growth factor | Activated | ACTA2,AMMECR1,ARFGEF1,ARIH2,ATXN7L1,BAX,BCL2L1,CCND2,CHD7,CLASP2 | | | | | | |  |  |
| INPP5F | phosphatase |  | CCNB1IP1,CCND2,CCNT2,CDK17,INHBA,KLHDC3,MAX,MEIS2,PPME1,RCBTB1 | | | | | | |  |  |
| INSIG1 | other | Activated | ATF4,CYP51A1,FDFT1,FDPS,HMGCR,HMGCS1,HSPA5,LSS,MVD,SQLE | | | | | |  |  |  |
| ITGB5 | other | Activated | CSNK1A1,EGFR,PRKDC,XRCC5,XRCC6 | | |  |  |  |  |  |  |
| ITPR2 | ion channel | Activated | ITPR1,NCAPH,TPR,UBE2R2 | |  |  |  |  |  |  |  |
| JMY | transcription regulator | Activated | BAX,BCL2,DDB1,DDB2,MLH1,TIGAR,TP53I3,XPC,XRCC5 | | | | |  |  |  |  |
| KDM1A | enzyme |  | ACSM1,APC,ATAD2,BLM,BMAL2,BRCA1,BRCA2,BRIP1,CENPF,CENPK | | | | | |  |  |  |
| KDM3B | enzyme | Inhibited | ALDH3A2,ANKRD13A,ARL6IP1,CIT,CKAP2,CMBL,DAPK1,DGKH,DLGAP5,FADS2 | | | | | | |  |  |
| KDM5A | enzyme | Inhibited | ANKRD13C,AP2A1,ARHGEF10,CD55,CHD8,CLASP2,CLOCK,COL1A2,DYNC1LI2,GCC2 | | | | | | | |  |
| KDM5B | enzyme | Inhibited | ANKRD36B,AP4S1,ARL6IP5,BBS9,BRCA1,BUB1B,BUB3,CCNB1,CDK1,DLGAP5 | | | | | | |  |  |
| KIF26A | other |  | PAK3,PIK3R1,PTK2,RAC1 | |  |  |  |  |  |  |  |
| KIF3A | enzyme | Inhibited | ACAT1,ACAT2,ACLY,CYP51A1,FDPS,HMGCR,IDI1,MVD | | | | |  |  |  |  |
| KLHL42 | enzyme |  | FN1,KATNA1,PPP2R5E | |  |  |  |  |  |  |  |
| KRT19 | other |  | EIF2AK3,EIF2S1,ERP29,HSPA5,MAML1,NUMB,PTEN,RBPJ | | | | |  |  |  |  |
| LAPTM4B | other |  | APP,BAX,BCL2,RHOA | |  |  |  |  |  |  |  |
| LARP1 | translation regulator | Inhibited | CTNNB1,EEF1A1,EEF1B2,EEF1D,EEF1G,EEF2,EIF3A,EIF3E,EIF3F,EIF3H | | | | | | |  |  |
| LDHB | enzyme | Inhibited | ACO2,CS,ENO1,GPI,HK1,PFKFB3,PGK1,PKM,SDHB,SDHD | | | | |  |  |  |  |
| LH (complex) | complex |  | ACTA2,ACTB,ACTG2,ACTR2,ADCY9,ALPL,AR,ARHGAP1,ARHGAP35,ARL6IP5 | | | | | | |  |  |
| LIN9 | other | Activated | BIRC5,BUB1,CCNB1,CDK1,CENPE,CEP55,DLGAP5,TMPO | | | | |  |  |  |  |
| LIN28A | other |  | CDH2,CDK1,FUS,HNRNPF,LATS1,LATS2,MOB1A,SAV1,STK3,STK4 | | | | | |  |  |  |
| LINC00945 | other |  | CDH2,ZEB1,ZEB2 |  |  |  |  |  |  |  |  |
| LLGL2 | other | Activated | AXL,EMP2,FSCN1,FSTL1,HMMR,LHFPL6,NDRG1,SGK1,SH2D4A,SHCBP1 | | | | | | |  |  |
| LONP1 | peptidase |  | ACAT1,ANXA2,ATF4,ATP5F1A,ATP5F1D,ATP5IF1,ATP5PF,CALU,CHCHD3,CLPX | | | | | | |  |  |
| MAGI1 | enzyme | Inhibited | ABLIM1,ACP1,ACSL3,ADD3,ADIPOR2,AIMP2,AMD1,ARID4A,ARL3,ATAD2 | | | | | | |  |  |
| MALAT1 | other |  | BAX,BCL2,BIRC5,LTBP3,TP53 | |  |  |  |  |  |  |  |
| MALAT1 | other | Activated | ANKHD1/ANKHD1-EIF4EBP3,BCL11A,CDH2,DNMT1,GINS1,HIF1A,HMGA2,KRAS,MRE11,NBN | | | | | | | |  |
| MALSU1 | other | Activated | COX4I1,MT-CO1,MT-CO2,MT-ND1,MT-ND4,MT-ND6 | | | |  |  |  |  |  |
| MAP2K5 | kinase | Activated | ACACA,ACSS2,CYP51A1,FDFT1,FDPS,HMGCR,MSMO1,TM7SF2 | | | | | |  |  |  |
| MAP3K12 | kinase |  | ACTB,ACTG1,APP,RPL12,RPL27A,RPL37,RPL37A,RPS18,RPS2,RPS8 | | | | | |  |  |  |
| MAPK1 | kinase |  | ACO1,ADAM17,ADAR,AR,ARHGAP11A,BCL11A,BCL2,BICRAL,BIRC5,BLZF1 | | | | | | |  |  |
| MAPK9 | kinase |  | ACACA,ACLY,BDP1,BRF1,CHERP,DMD,EIF4E,GAPDH,GPAT4,HMGN2 | | | | | |  |  |  |
| MASTL | kinase | Activated | ACVR1,ADSS2,ANLN,ASAP1,ATP2A2,BMPR2,CCDC50,CUL5,DENR,DICER1 | | | | | | |  |  |
| MAX | transcription regulator |  | APEX1,BAX,BCR,CAD,CBX5,CCND2,CSDE1,DKC1,DLEU1,DLEU2 | | | | | |  |  |  |
| MBD2 | transcription regulator |  | APOE,ATP5ME,BCAT2,BRCA1,CDH2,COX6C,DDB2,ESR1,GSTP1,MEF2A | | | | | |  |  |  |
| MDGA2 | other | Activated | ATR,BID,MSH2,WT1 |  |  |  |  |  |  |  |  |
| MDM2 | enzyme |  | BCL2,CASP2,CDH2,DNMT1,E2F3,ELF4,ESR2,EZH2,FCGRT,FOXO3 | | | | | |  |  |  |
| MEG3 | other |  | ACTA2,COL1A1,FN1,HIF1A,MDM2,PHLPP1,TP53,VIM,ZEB1,ZEB2 | | | | | |  |  |  |
| MIEF2 | other | Activated | ACACA,CDH2,HMGCR,HMGCS1,SCD,SREBF2,VIM | | | |  |  |  |  |  |
| mir-2392 | microRNA |  | MT-CO1,MT-CO2,MT-CYB,MT-ND2,MT-ND4,MT-ND5,PKM | | | | |  |  |  |  |
| mir-122 (includes others) | microRNA |  | ABCA1,ADAM10,CUX1,FDFT1,FDPS,GALNT10,HMGCR,HMGCS1,IGF1R,MVD | | | | | | |  |  |
| mir-137 (includes others) | microRNA |  | BCL11A,DNMT1,FSTL1,KDM4A | | |  |  |  |  |  |  |
| mir-142 (includes others) | microRNA |  | ABAT,CAT,CLIC4,ERP44,EZR,HIF1A,HMGCS1,LIMA1,PAICS,PCMT1 | | | | | |  |  |  |
| mir-199 (includes others) | microRNA | Inhibited | CCDC88A,CDH2,CTNNB1,DNMT3A,HIF1A,MTOR,VIM | | | | |  |  |  |  |
| mir-203 (includes others) | microRNA |  | BIRC5,EZH2,RNF2,SUZ12 | |  |  |  |  |  |  |  |
| mir-21 (includes others) | microRNA | Inhibited | ACTA2,APAF1,ARF4,BMPR2,C8orf44-SGK3/SGK3,FAM3C,FBXO11,FN1,GAS5,GLCCI1 | | | | | | | |  |
| mir-34 (includes others) | microRNA | Inhibited | AHCYL1,BAX,BCL2,HIF1A,PFKFB3,PKM,SIRT1,STIM1,STMN1,TP53 | | | | | |  |  |  |
| mir-486 (includes others) | microRNA | Inhibited | AFF3,ANKRD12,ARID4B,DCBLD2,FOXO1,FOXP1,PTEN,RFFL,SLC10A7,SLC4A8 | | | | | | |  |  |
| mir-515 (includes others) | microRNA | Inhibited | CDH2,ERCC8,HIF1A,KHSRP,SMAD4,STAT3,UHRF1,VIM,ZEB2 | | | | |  |  |  |  |
| mir-542 (includes others) | microRNA |  | FOXM1,NRP1,VEGFA | |  |  |  |  |  |  |  |
| mir-8 (includes others) | microRNA |  | ATG5,EZH2,HDAC3,HMGB1,MOK,PGR,PPP2R1B,PTEN,RAC1,RNF2 | | | | | |  |  |  |
| MIR99A/LET7C/MIR125B2 (family) | group | Inhibited | BMPR1A,BMPR2,SMAD2,SMAD4,TGFBR1 | | | |  |  |  |  |  |
| MITF | transcription regulator | Activated | ACAN,ATRX,AURKB,BCL2,BRCA1,CCNB1,CCNF,CD151,CD44,CENPF | | | | | |  |  |  |
| MKI67 | other | Activated | MCM4,MCM5,RFC1,RFC4 | |  |  |  |  |  |  |  |
| MMP9 | peptidase | Activated | AXL,BCL2L1,CD44,CD99,EXOC2,FOXP1,MDK,PBX1,PRKDC,SERPINE2 | | | | | |  |  |  |
| MMP12 | peptidase |  | ACTR2,ACTR3,ADK,APP,ARPC1B,CAPZA1,CAPZA2,CCT5,DNM1L,EIF3F | | | | | |  |  |  |
| MRPL12 | other | Activated | MT-CO1,MT-CO2,MT-ND1,MT-ND2,MT-ND6 | | | |  |  |  |  |  |
| MRPL14 | other | Activated | COX4I1,MALSU1,MT-CO1,MT-CO2,MT-ND1,MT-ND4,MT-ND6 | | | | |  |  |  |  |
| MT-TE | other |  | MT-ATP6,MT-CO2,MT-CYB,MT-ND1,MT-ND4,MT-ND5,MT-ND6 | | | | |  |  |  |  |
| MTLN | other |  | BRCA1,BRCA2,FANCD2,FANCI,MDM2,RMI2,TP53 | | | |  |  |  |  |  |
| MYBL2 | transcription regulator | Activated | BCL2,BIRC5,BUB1,CCNB1,CDH2,CDK1,CENPE,CTNNB1,FOXM1,IGFBP5 | | | | | |  |  |  |
| MYC | transcription regulator | Activated | ABCC1,ABCC4,ACACA,ACADM,ACAN,ADAM9,AFF4,ALDH18A1,AOPEP,APEX1 | | | | | | |  |  |
| MYCBP | transcription regulator | Activated | CAD,CCND2,EIF2A,LDHA,NCL | |  |  |  |  |  |  |  |
| MYCN | transcription regulator | Activated | ABCC1,APEX1,BCL2L1,CCND2,EGFR,HDAC5,LDHA,MDM2,MRE11,NBN | | | | | |  |  |  |
| MYO1B | other | Activated | GAPDH,GPI,HIF1A,LDHA,PGK1,PKM | | |  |  |  |  |  |  |
| N6AMT1 | enzyme | Activated | ALDH7A1,CD320,CDCA7,FADS1,MPZL1,PAICS,PPAT,PRSS23,RB1,REST | | | | | | |  |  |
| NCL | other |  | DICER1,ILF3,LAMB1,MDM2,PDCD4,PTEN,TP53,UBE4A | | | | |  |  |  |  |
| NDRG3 | other |  | ANLN,BNIP2,CALU,CBFA2T2,CEP44,DCBLD2,DCP2,DDX10,DPYSL3,EEF1G | | | | | | |  |  |
| NDUFA13 | enzyme |  | BAX,BCL2,BCL2L1,CDH2,CTTN,HIF1A,TFDP1,VIM | | | |  |  |  |  |  |
| NEUROG1 | transcription regulator | Inhibited | ADD3,AMIGO2,C1S,CCND2,CEMIP,COL3A1,FN1,GASK1B,GREM1,INHBA | | | | | |  |  |  |
| NFE2L2 | transcription regulator | Activated | ACSS2,ATF4,ATF6,BRCA1,CAT,COX4I1,CS,EIF2AK3,FTH1,FTL | | | | | |  |  |  |
| NME1 | kinase |  | ACIN1,BCL2,COIL,EXOSC8,GEMIN5,GRSF1,HNRNPA2B1,NOP58,PA2G4,PABPC1 | | | | | | |  |  |
| NOTCH1 | transcription regulator | Activated | ACTA2,BCL2,CD44,CTNNB1,EGFR,HEY2,HIF1A,IGF1R,NR2F2,PTPRK | | | | | |  |  |  |
| NPPB | other | Inhibited | ACAT2,CYP19A1,FDFT1,FDXR,HMGCR,HMGCS1,IDI1,LDLR,LSS,MSMO1 | | | | | |  |  |  |
| NR1D1 | ligand-dependent nuclear receptor | Activated | ACACA,ACLY,FADS2,MDH1,ME1 | | |  |  |  |  |  |  |
| NR3C1 | ligand-dependent nuclear receptor |  | ABHD2,ABL1,ACAT1,ACTA2,AKT2,AMIGO2,APOE,APTX,ARID4B,ATG12 | | | | | |  |  |  |
| NR4A1 | ligand-dependent nuclear receptor | Activated | ACTA2,BABAM2,BCL2,BIRC5,COL1A1,COL1A2,DAPK1,EGFR,FN1,GREM1 | | | | | | |  |  |
| NRF1 | transcription regulator | Activated | CD47,CDH2,HPCAL1,IDE,NCALD,PSMB7,PSMC1,PSMC4,PSMC6,PSMD14 | | | | | | |  |  |
| NSD2 | transcription regulator |  | BACE2,BAX,BCL2,CCND2,ERCC1,HAT1,HDAC4,IGF2BP2,ITGB1,KMT2C | | | | | |  |  |  |
| NSRP1 | other |  | CDH2,FN1,TGFBR1,VIM | |  |  |  |  |  |  |  |
| NSUN2 | enzyme | Activated | MT-ND4,PCYT1A,PIK3R1,SHOC2,SREK1 | | |  |  |  |  |  |  |
| NSUN3 | enzyme | Activated | MT-ATP6,MT-CO1,MT-CO2,MT-CYB,MT-ND2,MT-ND4,MT-ND4L,MT-ND5 | | | | | |  |  |  |
| NSUN6 | enzyme | Activated | AXL,DAB2,DDAH1,EMP2,FLNA,FSCN1,FSTL1,HMMR,LHFPL6,MARCKS | | | | | |  |  |  |
| NTRK1 | kinase | Inhibited | AARS1,APOE,ARHGAP6,ARSB,ATAD5,ATF5,BAMBI,BRIP1,CALR,CANX | | | | | |  |  |  |
| NUDT21 | other |  | ACACA,ADPGK,COL1A1,FCHO2,LAPTM4B,MTO1,NBEA,SCAP,SPARC,TEX2 | | | | | | |  |  |
| NUP107 | other | Activated | NUP133,NUP153,NUP214,NUP98,RANBP2,TPR | | | |  |  |  |  |  |
| NUP133 | other | Activated | NUP107,NUP153,NUP98,TPR | |  |  |  |  |  |  |  |
| NUPR1 | transcription regulator | Inhibited | ABCC5,ABL2,ACAD10,ACTR3B,ADCY9,ALG8,ANGEL1,ANP32A,ANP32E,API5 | | | | | | |  |  |
| OGA | enzyme | Inhibited | ABLIM1,ACSS2,ACTG1,AIFM1,AIFM2,APOBEC3G,ARHGAP29,AVEN,BACH1,BAX | | | | | | |  |  |
| OSBPL8 | transporter | Activated | ATF4,EIF2AK3,EIF2S1,HSPA5 | |  |  |  |  |  |  |  |
| OVOL2 | transcription regulator | Inhibited | ARHGAP31,ARPC1B,ATM,BTRC,CEP135,CEP63,ENO1,FAM13A,FOXM1,GPI | | | | | | |  |  |
| PAPOLA | enzyme |  | MT-ATP6,MT-CO1,MT-CO2,MT-CO3 | | |  |  |  |  |  |  |
| PARG | enzyme |  | ITPR1,LGALS3BP,MTOR,MTR,NELL2,NFAT5,NVL,PTEN,RAPGEF4,TMOD3 | | | | | |  |  |  |
| PAWR | transcription regulator |  | APP,BCL2,CCAR1,CDC42,RAC1 | | |  |  |  |  |  |  |
| PAX5 | transcription regulator |  | BCL2,BCL2L1,CTNNA1,CTNNB1,DLEU2,FN1,TP53,VIM | | | | |  |  |  |  |
| PAX3-FOXO1 | fusion gene/product |  | ABAT,ANXA5,CAP2,CCT5,CD276,CD63,CHD7,CLIC1,CNBP,DAPK1 | | | | | |  |  |  |
| PCBP2 | other |  | BRCA1,BRCA2,FANCD2,FANCI,MDM2,RMI2,TP53 | | | |  |  |  |  |  |
| PCGEM1 | other | Activated | ACACA,ACLY,ACOX1,CS,ENO1,GAPDH,GLUD1,GPI,GSR,IDH1 | | | | | |  |  |  |
| PCGF2 | transcription regulator |  | CDH2,CTNNB1,FN1,NFKB1,SMAD3,TP53,VIM,ZEB1,ZEB2 | | | | |  |  |  |  |
| PDK4 | kinase | Activated | HIF1A,KRAS,PC,PKM,RHEB | |  |  |  |  |  |  |  |
| PIMREG | other | Activated | ACACA,ACAT1,ACLY,BCL2,CDH2,EGFR,IFI27,ING5,MTOR,STAT3 | | | | | |  |  |  |
| PKM | kinase |  | ACLY,ACOX1,BCL2L1,CDH2,ENO1,GAPDH,GSR,ITGB1,LDHA,LSS | | | | | |  |  |  |
| PLA2R1 | transmembrane receptor | Activated | ACSL3,ACSL4,AK3,ALDH2,ATP5F1B,BDH2,COX5B,CYB5R3,DPYSL2,HAGH | | | | | | |  |  |
| PLD3 | enzyme |  | MT-ATP6,MT-CO2,MT-ND1 | |  |  |  |  |  |  |  |
| PML | transcription regulator | Inhibited | BIRC5,EGFR,ELOVL6,HMGCR,IDI1,LDLR,ME1,SREBF2 | | | | |  |  |  |  |
| POGLUT3 | enzyme | Activated | ATF4,ATG4B,EIF2AK3,HIF1A,HSPA5,SQSTM1,VEGFA,ZEB2 | | | | |  |  |  |  |
| POLG | enzyme |  | AR,MT-CO2,MT-CO3 | |  |  |  |  |  |  |  |
| POLR1HASP | other |  | HLA-A,HSP90AA1,ZEB1 | |  |  |  |  |  |  |  |
| POLR3G | enzyme |  | CDH2,EZR,KLF6,LMNA,PFDN5,RAC1,RFC2,TP53,VIM | | | | |  |  |  |  |
| POLRMT | enzyme |  | MT-ATP6,MT-CO1,MT-CYB,MT-ND1,MT-ND6 | | | |  |  |  |  |  |
| PPOX | enzyme |  | ARL6IP6,CACNA2D1,COX6A1,COX6B1,COX6C,CYP51A1,MGMT,MT-CO3,NDUFA4,UQCRH | | | | | | | |  |
| PPP1R1B | phosphatase | Activated | BCL2,BCL2L1,CD44,CFLAR,CTNNB1,ERBB2,SRSF3 | | | |  |  |  |  |  |
| PRKAR1A | kinase |  | EGFR,ERBB2,PPARG,PRKAR2B,SMAD3,TUSC3 | | | |  |  |  |  |  |
| PRKG1 | kinase |  | ACTA2,BCL2,MRE11,RAD1,TAGLN | | |  |  |  |  |  |  |
| PRKN | enzyme | Inhibited | ACTA2,COX4I1,CTNNB1,EGFR,MAOB,SQSTM1,TOMM20,TP53 | | | | |  |  |  |  |
| PRL | cytokine | Activated | ADAR,ATP2A2,BCL2,BST2,CCNB1,CPD,CTSB,DDX60L,EGFR,EIF2AK2 | | | | | |  |  |  |
| PRRC2B | other |  | CCND2,CRKL,YWHAZ | |  |  |  |  |  |  |  |
| PSEN1 | peptidase | Inhibited | APP,FDFT1,HMGCR,LDLR,SCD | | |  |  |  |  |  |  |
| PSMB5 | peptidase |  | ATG5,ATG7,PSMB1 |  |  |  |  |  |  |  |  |
| PSMD10 | transcription regulator | Activated | GNAS,HIF1A,IDH2,IKBKB,ME2,MRAS,PGD,PIK3CA,TALDO1,TIGAR | | | | | |  |  |  |
| PSME3 | peptidase |  | ACTA2,ENO1,GAPDH,LDHA,PFKL,PGM1,PKM,TAGLN,TPI1 | | | | |  |  |  |  |
| PTBP1 | enzyme |  | CD44,CDC42,CIRBP,CTNNB1,LDHA,LDLR,PKM,TPM4,ZC3HAV1 | | | | | |  |  |  |
| PTBP3 | other | Activated | CDH2,FN1,VIM,ZEB1,ZEB2 | |  |  |  |  |  |  |  |
| PTCD1 | other | Activated | MRPL4,MT-ATP6,MT-CO2,NDUFB8,NDUFB9,SDHB,UQCRB,UQCRC2 | | | | | |  |  |  |
| PTP4A3 | phosphatase |  | CDH2,FN1,HDAC2,HSP90AA1,JUP,LEO1,PTEN,STMN1 | | | | |  |  |  |  |
| PTPN2 | phosphatase | Activated | EGFR,MT-CYB,MT-ND6,STAT1,STAT6 | | |  |  |  |  |  |  |
| PTPN6 | phosphatase | Inhibited | BCL2L1,CDH2,FN1,STAT3,VIM | |  |  |  |  |  |  |  |
| PTPRR | phosphatase | Inhibited | AAMP,AP1M1,BTBD2,CALCOCO1,CCDC92,CDC37,CLASRP,CLCN7,COL6A1,DNMT1 | | | | | | | |  |
| PUM1 | other |  | AGO2,ANK2,APP,CALM1 (includes others),CNOT1,FOXP1,GSK3B,KLHL24,LAPTM4A,REEP5 | | | | | | | |  |
| PURPL | other | Activated | CENPJ,CNTRL,KIF20B,KIF2C,NUP50,PIF1,RACGAP1,RFC1,SMC3,TMPO | | | | | |  |  |  |
| RAB1B | other | Activated | ARF4,COPG1,CYTH1,GOLGA1,GOLGA2,GOLGA3,GORASP2,RABAC1,RHOBTB3,SEC24D | | | | | | | |  |
| RABL6 | other | Activated | ABAT,ATXN1,AURKB,BUB1,BUB1B,CCNB1,CDC25C,CENPF,CHEK1,CHEK2 | | | | | | |  |  |
| RAC2 | enzyme | Activated | ARPC3,BCL2,BCL2L1,DIAPH1,DIAPH2,MACF1 | | | |  |  |  |  |  |
| RAD51 | enzyme |  | ATM,BAX,BRIP1,CD44,FANCD2,GPX4,HDAC6,MYO1E,NEDD9,NUSAP1 | | | | | |  |  |  |
| RAPGEF1 | other | Activated | DHX38,EFTUD2,KDM5A,SNRNP200,SNRNP70,SRSF2 | | | | |  |  |  |  |
| RARA | ligand-dependent nuclear receptor |  | AASS,ABHD2,ABLIM1,ADAM17,ANO10,APOA1,ARHGAP1,ASPM,ATG13,BAZ2A | | | | | | |  |  |
| RB1 | transcription regulator | Inhibited | ACTB,ALG5,ARHGAP11A,BAX,BCL2,BIRC5,BRCA1,BUB1,CASP6,CBX5 | | | | | |  |  |  |
| RBFOX2 | transcription regulator |  | ARNT,PLOD2,PTEN |  |  |  |  |  |  |  |  |
| RBL2 | other |  | AURKB,BRCA1,BUB1,CCNB1,CDK1,FOXM1,NPAT,PA2G4,RB1,RBL1 | | | | | |  |  |  |
| RBX1 | enzyme | Activated | BCL2,BIRC5,CCNB1,CDK1 | |  |  |  |  |  |  |  |
| RC3H1 | enzyme | Inhibited | APOBEC3C,BST2,CD44,ENO1,IFI27,IFI44,IFI44L,IFI6,IFITM3,IRF9 | | | | | |  |  |  |
| RCN3 | other | Activated | ATF4,CALR,ERBB2,FAU,HSPA5,RPL11,RPL12,RPL13,RPL13A,RPL23 | | | | | |  |  |  |
| RFX1 | transcription regulator |  | ALMS1,COL1A2,DCDC2,DNAAF4,KIF3A | | |  |  |  |  |  |  |
| RFX2 | transcription regulator |  | ALMS1,DCDC2,DNAAF4 | |  |  |  |  |  |  |  |
| RHOJ | enzyme | Inhibited | BOD1L1,CHAF1A,MCM3,MCM4,MCM5,MCM7,MCMBP,PRIM1,RFC3 | | | | | |  |  |  |
| RNA POLYMERASE II (complex) | complex |  | ACTB,B2M,BCL2,BRCA1,CAP2,CBX5,CCNB1,CDH11,CHEK2,COL1A2 | | | | | |  |  |  |
| RNF5 | enzyme |  | ACACA,FDFT1,HMGCR,SREBF2 | | |  |  |  |  |  |  |
| RNF34 | enzyme |  | BCL2,BCL2L1,MDM2,TP53 | |  |  |  |  |  |  |  |
| RNF181 | enzyme | Activated | ARL3,CDK1,ESR1,GREB1,HDDC2,MED13L,MED28,MTDH,PDCD4,SNX24 | | | | | |  |  |  |
| ROR1 | kinase | Activated | AURKB,AXL,EMP2,FSTL1,LHFPL6,MDFIC,NDRG1,SGK1,STMN1,TOP2A | | | | | |  |  |  |
| RP11_394O46 | other |  | BAX,BCL2,PDCD5,TP53 | |  |  |  |  |  |  |  |
| RPA1 | other | Inhibited | CNIH3,EEFSEC,FOCAD,NAV2,PCNX1,TANC2,TPD52L1 | | | | |  |  |  |  |
| RPS7 | other |  | BCL2L1,PIK3R1,TP53 | |  |  |  |  |  |  |  |
| RPS11 | other |  | APAF1,HMGA2,PDCD4,PTEN | |  |  |  |  |  |  |  |
| RSF1 | transcription regulator | Activated | BCL2,BCL2L1,CFLAR,XIAP | |  |  |  |  |  |  |  |
| RUNX1-RUNX1T1 | fusion gene/product |  | AFF2,AGO2,CALM1 (includes others),DCPS,DLG5,DYRK1A,ELF4,ETV6,FOXO1,HNRNPDL | | | | | | | |  |
| 26S PROTEASOME (complex) | complex |  | ATG5,ATG7,ATP6V0E1,ATP6V1D,ATP6V1H,BIRC2,BRCA2,CDK1,CENPE,CFLAR | | | | | | |  |  |
| S100A4 | other |  | ACTA2,COL1A1,CTNNB1,NOTCH2,PTEN,TIMP1,TIMP2,VIM | | | | |  |  |  |  |
| SAFB2 | other |  | B2M,BCCIP,CHD3,EIF4B,HEXA,HLA-C,PPP1R12A,RPL35A,SAFB2,UBE2B | | | | | | |  |  |
| SAMMSON | other | Inhibited | ATP5F1B,MRPL3,MRPS18C,MRPS27,MRPS28,MRPS5,MRPS6,MRPS9,MT-ND1,MT-ND6 | | | | | | | |  |
| SCAP | other | Activated | ACACA,CDH2,FDFT1,HMGCR,LDLR,SCD,SF3A3,SMAD2,SQSTM1,SREBF2 | | | | | | |  |  |
| SETBP1 | transcription regulator |  | AKT2,AR,BARD1,BCL11A,BRCA2,BRD3,BRD4,BUB1B,CARD11,CCNC | | | | | |  |  |  |
| SETDB1 | enzyme | Inhibited | ACTA2,CDH2,FN1,GPX4,IFI27,IFI44,IFI44L,RIGI,ZNF420 | | | | |  |  |  |  |
| SF3B1 | other |  | ATP5F1A,MDM4,MT-CO2,NDUFB8,PHGDH,UQCRC2 | | | |  |  |  |  |  |
| SFPQ | other | Activated | ACIN1,AR,CHERP,HNRNPU,PRPF3,SF1,SF3A2,SF3B2,SF3B3,U2SURP | | | | | |  |  |  |
| SIRT1 | transcription regulator |  | ATXN10,BAX,BCL2,BIRC5,CCND2,CD63,CD81,CDH2,CYP19A1,ENO1 | | | | | |  |  |  |
| SLC29A1 | transporter |  | BAX,CCNG1,FDXR,MDM2,PPM1D,RPL10A,RPL3,RPS27L,SESN1,SNRPG | | | | | |  |  |  |
| SLC2A3 | transporter | Activated | GAPDH,GPI,LDHA,PFKFB3,PFKL,PGAM1,PHGDH,PKM,SLC16A4,TPI1 | | | | | |  |  |  |
| SLC39A8 | transporter | Inhibited | DAB2,FTH1,FTL,GUSB,SYNGR1 | | |  |  |  |  |  |  |
| SLIT2 | other | Activated | ARID1A,BTRC,CTNNB1,EZH2,PAPPA,SMARCA2,SMARCA4,SMARCB1 | | | | | |  |  |  |
| SMOC2 | other |  | ACTA2,FN1,VIM |  |  |  |  |  |  |  |  |
| SNHG6 | other |  | EZH2,HIF1A,STAT3 |  |  |  |  |  |  |  |  |
| SNHG11 | other |  | AK4,BSG,CCNB1,CCND2,CDH2,CTNNB1,ENO1,GSK3B,IGF1R,ROCK1 | | | | | |  |  |  |
| SOX4 | transcription regulator | Activated | AZI2,BAX,BIRC5,CDH2,CTNNB1,DICER1,EZH2,HDAC8,MAP4K5,NRP1 | | | | | |  |  |  |
| SP4 | transcription regulator | Activated | BIRC5,EGFR,HSPA5,IGF1R,MAT2A,NFKB1,PDE6B,SOD2,STAT3,VEGFA | | | | | |  |  |  |
| SPDEF | transcription regulator | Inhibited | APC,CDH11,CDH2,COL1A1,COL4A1,COL4A2,COL6A1,COL6A2,COL6A3,CYFIP1 | | | | | | |  |  |
| SPOP | other |  | BRD2,BRD3,BRD4,FDFT1,MRE11,MVD,RAC1,TDP1 | | | |  |  |  |  |  |
| SPP1 | cytokine | Activated | BAX,BCL2,CCS,CD44,CDH2,COX7A2L,CTNNB1,FN1,FOXRED1,GATA4 | | | | | |  |  |  |
| SREBF1 | transcription regulator | Activated | AARS1,ACACA,ACLY,AK4,APOBEC3F,ARF4,ATP5ME,CAP2,CAPZB,CFI | | | | | |  |  |  |
| SRF | transcription regulator |  | ACTA2,ACTG2,APOLD1,CAP1,CAPRIN1,DSTN,EPG5,EXTL2,EZR,FHL2 | | | | | |  |  |  |
| SRPK1 | kinase |  | ATF4,BCL2L1,INSR,RAC1,VEGFA | | |  |  |  |  |  |  |
| SRSF1 | other |  | ACLY,ACSS2,BIRC5,FDFT1,FN1,HMGCS1,KLF6,LMNA,RAC1,SCD | | | | | |  |  |  |
| SRSF3 | other |  | BCL2,CD44,E2F7,FOXM1,HIPK2,PDCD4,SIAH1,TP53,VEGFA | | | | |  |  |  |  |
| SSBP1 | other | Inhibited | COX4I1,NDUFS1,SDHC,UQCRC1 | | |  |  |  |  |  |  |
| STOX1 | other |  | ACTG1,CALM1 (includes others),CLASP1,PSMF1,RAB10,RPL17,SRSF7 | | | | | |  |  |  |
| STX3 | transporter |  | ANAPC5,CAMKK2,FNTA,KDM2B,RPL30,SART3,SULT1C4,VCAN | | | | | |  |  |  |
| STX18 | transporter | Activated | ANLN,ARHGAP29,BUB1,CDC7,CEP250,CNTRL,COL6A2,CSNK1D,DDB2,DGCR8 | | | | | | |  |  |
| SUZ12 | enzyme |  | ABL2,AKR7A2,ATP2A2,CALU,CCPG1,CTNNA1,DAB2IP,DAZAP2,DNAJB6,EIF3A | | | | | | |  |  |
| SYVN1 | transporter | Activated | ABCC4,ACSL3,ADAM9,ADCY9,ANO10,APLP2,APP,ATP1A1,ATP1B3,AXL | | | | | |  |  |  |
| TAF1D | other | Activated | ANAPC1,BUB1,BUB1B,CDC14A,CDC23,CDK1,TTK,YWHAG,YWHAQ,YWHAZ | | | | | | |  |  |
| TAL1 | transcription regulator |  | AATF,ACOT8,ADCY3,AFDN,ASPM,ATP6AP1L,BCOR,BRF1,BTBD3,BUB1 | | | | | |  |  |  |
| TCF4 | transcription regulator |  | ADAM17,ADAMTS9,APOBEC3G,ARHGEF6,B4GALT1,BAG2,BARD1,BCL2L13,BEX3,BMPR1A | | | | | | | |  |
| TCR (complex) | complex | Activated | ABCD3,ABLIM1,AK2,APOE,ARHGEF1,ATP5F1B,BCL2L1,BSG,CARD8,CDK13 | | | | | | |  |  |
| TFAP2A | transcription regulator |  | ABCA1,BCL2,BCL2L1,BIRC5,BLOC1S6,CCN5,DCBLD2,DCLK1,ERBB2,EYA4 | | | | | | |  |  |
| TFE3 | transcription regulator | Activated | ATP5PF,ATP6V0E1,ATP6V1C1,ENO1,IRS1,LAMP1,LDHA,PPARGC1B,VEGFA,VPS26A | | | | | | | |  |
| TFEB | transcription regulator | Activated | ACACA,ACADM,ANAPC1,ARSB,ATG5,ATP2B1,ATP6V0E1,ATP6V1H,BUB1B,CCNB1 | | | | | | |  |  |
| TGFBR2 | kinase |  | ACSL3,ACTA2,AP3S1,ATF4,CCT5,CFL1,CHRNA7,EXT2,GGA3,GSTP1 | | | | | |  |  |  |
| THEM6 | other | Activated | ACAA1,ATF4,CYP51A1,ERG28,ERP44,FDFT1,FDPS,HMGCS1,HSP90B1,HSPA5 | | | | | | |  |  |
| THOC5 | other | Activated | COX20,DTD2,METTL21A,TMBIM4,TMCC1,TMED10 | | | |  |  |  |  |  |
| TIA1 | other |  | ADAM9,ARID2,ARL6IP5,ASPM,AXL,C1GALT1,CALM1 (includes others),CAMKK2,CCNB1,CD276 | | | | | | | | |
| TMTC3 | enzyme |  | CDH2,EIF2AK3,FAM3C,VIM | |  |  |  |  |  |  |  |
| TNFRSF9 | transmembrane receptor | Activated | BCL2L1,BLM,BRCA1,CCND2,CDC25C,CDC45,CDC7,CHEK1,CLSPN,DNA2 | | | | | | |  |  |
| TNFSF10 | cytokine | Inhibited | BCL2,BCL2L1,BIRC2,BIRC5,CFLAR,CSE1L,PRKCE,XIAP | | | | |  |  |  |  |
| TNFSF13B | cytokine |  | BAX,BCL2,BCL2L1,TP53,XIAP | |  |  |  |  |  |  |  |
| TOP1MT | enzyme | Activated | ATP5F1A,MT-CO2,MT-ND6,NDUFB8,SDHB,UQCRC2 | | | |  |  |  |  |  |
| TOR1AIP1 | other | Activated | BUB1B,CDK1,CENPF,DAG1,DLGAP5,GINS2,GRB2,HADH,HES6,HJURP | | | | | |  |  |  |
| TP53 | transcription regulator |  | ABCC1,ABCD4,ACAA1,ACLY,ACO1,ACOX1,ACOX3,ACSL3,ACTA2,ACTB | | | | | |  |  |  |
| TP73 | transcription regulator | Activated | ABCC1,ACTA2,ADAM17,AUH,BAX,BID,BIRC5,BLZF1,CASP2,CDC25C | | | | | |  |  |  |
| TRAP1 | enzyme | Inhibited | AK3,ATP5F1D,ATP5ME,BCL2L13,CHCHD3,COX5A,COX5B,ECSIT,HIBADH,HSPD1 | | | | | | |  |  |
| TREX1 | enzyme | Inhibited | ACAT1,APP,ATP2C1,B2M,BST2,CAST,CD44,CD47,DDX60,EIF2AK2 | | | | | |  |  |  |
| TRIB3 | kinase |  | HIF1A,JAK1,LDHA,LPL,PSPH,WWTR1 | | |  |  |  |  |  |  |
| TRIM2 | enzyme | Activated | FTL,GCLC,GSR,ITGB1,MSRA,PRDX2,PTK2,PTPA,SOD1,TXN | | | | |  |  |  |  |
| TRIM65 | enzyme |  | IMMP1L,PDCD4,PTEN | |  |  |  |  |  |  |  |
| TRPS1 | transcription regulator | Inhibited | AURKB,BUB1,CDC25C,CDK1,CENPF,FN1,FOXM1,GREB1,KIF11,NOTCH2 | | | | | | |  |  |
| TSPYL5 | other | Activated | ASB3/GPR75-ASB3,AZIN1,CAST,CYP19A1,DTD1,FBXW7,FUT8,GLDN,NR3C2,PTBP2 | | | | | | | |  |
| TWIST1 | transcription regulator | Activated | ACOX1,ACTA2,AKT2,AR,AXL,BAX,BCL2,BST2,CD44,CDH2 | | | | |  |  |  |  |
| TWIST2 | transcription regulator | Activated | CD44,CDH2,CTNNB1,FN1,NRF1,PRKAA1,PRKAA2,SIRT1,STK11,TGFB1 | | | | | |  |  |  |
| TXNIP | other |  | CS,ENO1,GAPDH,GSDMD,HIF1A,IDH1,LDHA,MDH2,PGK1,TPI1 | | | | | |  |  |  |
| UBA1 | enzyme | Inhibited | ANXA2,ANXA5,APOE,BIRC2,HSP90B1,HSPA5,HSPA8,TAGLN,TUBA1C,VIM | | | | | | |  |  |
| UBA2 | enzyme | Activated | EZH2,PIAS1,SENP1,TP53,UBE2I | | |  |  |  |  |  |  |
| UPF1 | enzyme | Inhibited | ADAM9,CD164,ERG,HMGA2,IQGAP1,MCM10,SGK1,SMG1,SMG5,SMG6 | | | | | |  |  |  |
| UQCC3 | other |  | ATP11A,ATP11B,ATP11C,ATP13A3,ATP1A1,ATP1B1,ATP1B3,ATP2A2,ATP2B1,ATP2C1 | | | | | | | |  |
| USP7 | peptidase | Activated | CAD,CCND2,CUL4A,DHX40,FKBP5,FLNA,GMPS,GPALPP1,GTF3C1,HLTF | | | | | | |  |  |
| VCP | enzyme |  | BCL2,CLNS1A,DIAPH2,FTH1,FYCO1,HSPA5,IFT25,KRAS,MAN1A1,MAN2A1 | | | | | | |  |  |
| WAKMAR1 | other | Activated | DOCK5,DYNLL1,EIF2AK3,GINS2,HMMR,ING4,KAT5,KIF11,LAMB2,NUF2 | | | | | |  |  |  |
| XBP1 | transcription regulator | Activated | ADAM10,APBB2,BCL2,CAT,COL4A1,CRK,DNAJC3,ERLEC1,ERP29,ESR1 | | | | | |  |  |  |
| YAP1 | transcription regulator | Activated | ACSF2,ACSL4,ACTA2,ADAM9,ANKRD46,ANLN,APLP2,ARHGEF6,ARID4B,ATAD2 | | | | | | |  |  |
| YARS2 | enzyme | Activated | COX16,COX5A,LONP1,MT-CO2,MT-CYB,MT-ND6,NDUFA9,NDUFB8,NDUFS1,SDHB | | | | | | |  |  |
| YY1 | transcription regulator | Activated | BCL2L1,BRCA1,CALR,DICER1,ERBB2,HSPA5,LMAN1,POLB,QKI,VEGFA | | | | | |  |  |  |
| ZBED1 | enzyme |  | RPL10A,RPL12,RPS6 | |  |  |  |  |  |  |  |
| ZBED6 | transcription regulator | Inhibited | ARHGEF1,DLGAP5,KCTD1,NASP,PMEPA1,PPAT,ROCK2,SGK1,SPTBN1,TCF7 | | | | | | |  |  |
| ZEB1 | transcription regulator | Activated | CDH2,COL1A1,COL1A2,CTNNB1,DNMT1,FGFR1,FN1,NRP1,POLQ,RAB6A | | | | | |  |  |  |
| ZHX2 | transcription regulator |  | AP2B1,COX20,COX7C,HIF1A,NDUFB9,OXSR1,SDHA,UQCRC1,WSB1 | | | | | |  |  |  |
| ZNF281 | transcription regulator | Activated | ACTA2,BLM,COL1A1,COL4A1,FANCA,FANCD2,FN1,MDC1,RAD9A,XRCC2 | | | | | | |  |  |
| ZNF598 | enzyme | Inhibited | DDX60,HERC5,IFI44,RSAD2 | |  |  |  |  |  |  |  |
