# Supplementary material for: Single‐nucleus and spatial transcriptomics of paediatric ovary: Molecular insights into the dysregulated signalling pathways underlying premature ovarian insufficiency in classic galactosemia
Source: Clin Transl Med. 2024 Oct 23;14(10):e70043. doi: 10.1002/ctm2.70043 (PMC11812122; doi:10.1002/ctm2.70043)
Supplement: Supplementary file 5 — Supporting information [file CTM2-14-e70043-s002.docx]

**Supplementary Table S4**

IPA Causal Network Analysis : Upstream regulator genes identified in the DEGs from oocyte cluster [SnRNAseq dataset: CG vs control]

| **Upstream Regulator** | **Molecule Type** | **Predicted Activation State** | **Target Genes in Dataset** |
| --- | --- | --- | --- |
| ESTROGEN RECEPTOR (family) | group |  | ANXA1,C3,CALD1,CCND2,CD59,CD99,COL4A5,COL6A1,COL6A2,CXCL12 |
| ACTG1 | other | Inhibited | ACTA2,CALD1,CNN1,MRTFA,TAGLN |
| EWSR1-FLI1 | fusion gene/product | Inhibited | CALD1,CD99,COL3A1,IGFBP5,MYH9,PDGFRA,SAMD5,SELENOP,SH3D19,TAGLN |
| FBXW7 | enzyme | Activated | HLA-DRA,MT-ATP6,MT-CO1,MT-CO2,MT-CO3,MT-CYB,MT-ND1,MT-ND4,MT-ND4L |
| GLIS1 | transcription regulator | Activated | APOD,COL1A2,COL6A2,FBLN1,MMP2,SPARC,TIMP3 |
| CST5 | other | Inhibited | AHNAK,ANXA2,CNBP,CYBRD1,DDX18,EEF1G,FHL1,FRAS1,GRSF1,HNRNPA2B1 |
| SMARCA4 | transcription regulator | Activated | A2M,AHNAK,CD74,CLK1,FHL2,GSTO1,HLA-B,HLA-C,HLA-DRA,HLA-E |
| TGFB2 | growth factor |  | ACTA2,CD59,COL1A1,COL6A2,HSPG2,MIF,MMP2,PLCH1,PTPRJ,TAGLN |
| BMP7 | growth factor |  | ACTA2,CALD1,IGF1,IGFBP3,IGFBP4,IGFBP5,MMP2,MSN,STS,VIM |
| NLRC5 | transcription regulator | Activated | B2M,HLA-A,HLA-B,HLA-C,HLA-E |
| SF1 | transcription regulator |  | ACTA2,CNN1,COL1A1,MYL6 |
| TGFB1 | growth factor | Activated | ACTA2,AOPEP,CALR,CCND2,CD59,COL1A1,COL1A2,COL3A1,HSPA5,IGF1 |
| FBN1 | other | Inhibited | COL1A2,COL3A1,LTBP3,TGFBR2,TIMP3 |
| CHI3L1 | enzyme |  | CALD1,COL6A1,COL6A2,EMP1,IGFBP5,TGFBR2 |
| MRPL14 | other | Activated | MT-CO1,MT-CO2,MT-ND1,MT-ND4 |
| MRPL12 | other |  | MT-CO1,MT-CO2,MT-ND1,MT-ND2 |
| MRTFB | transcription regulator | Activated | CALD1,COL3A1,IGFBP5,MYH9,PDGFRA,SAMD5,SELENOP,SH3D19,TAGLN |
| AHR | ligand-dependent nuclear receptor | Inhibited | ABTB3,ACTA2,COL1A1,COL3A1,COL6A1,COL6A2,CTSD,FBLN1,MGP,MT-ND2 |
| EN1 | transcription regulator |  | ACTA2,COL1A1,COL1A2 |
| SMAD2 | transcription regulator | Activated | ACTA2,CCNG2,COL3A1,MMP2,TGFBR2,TIMP3,VIM |
| TEAD4 | transcription regulator | Activated | CALD1,COL3A1,IGFBP5,MMP2,SAMD5,SH3D19,TAGLN,VIM |
| CEBPB | transcription regulator | Activated | ACTA2,AEBP1,C3,COL1A1,COL1A2,COL3A1,FSTL1,IGF1,IGFBP3,MMP2 |
| SMAD3 | transcription regulator | Activated | ACTA2,C3,CCNG2,CD59,COL1A1,COL1A2,COL3A1,SPARC,TIMP3,VIM |
| SORL1 | transporter | Activated | B2M,C3,COL1A1,COL1A2,COL3A1,COL6A1,CXCL12,FBLN1,HLA-E,LAMA4 |
| EBI3 | cytokine | Activated | B2M,HLA-A,HLA-B,HLA-C,HLA-DRA |
| OGA | enzyme | Inhibited | ACTG1,CCNG2,CPE,FBLN1,FLNA,GLUL,IGFBP4,IGFBP5,JAK1,PLPP3 |
| GFM2 | translation regulator |  | MT-CO2,MT-ND2,MT-ND5 |
| MED16 | transcription regulator |  | MMP2,TAGLN,TGFBR2,VIM |
| MYOCD | transcription regulator |  | CNN1,COL1A1,COL1A2,COL3A1,DES,MYH11,TAGLN |
| APLN | other |  | ACTA2,TAGLN,TGFBR2,VIM |
| SMC3 | enzyme |  | CCNG2,CXCL12,HLA-DRA,SOX4 |
| TEAD2 | transcription regulator | Activated | CALD1,COL3A1,IGFBP5,SAMD5,SH3D19,TAGLN |
| NODAL | growth factor |  | ACTA2,CCNG2,DES |
| SLC25A5 | transporter |  | MT-CO2,MT-ND2,MT-ND5 |
| TIA1 | other |  | ARL6IP5,PDCD5,S100A6,SOX6,TAGLN,TGFBR2,TIMP3,TXNIP |
| S100A4 | other |  | ACTA2,COL1A1,MMP2,VIM |
| NORAD | other | Activated | COL6A2,HSPA5,IGFBP3,IGFBP4,PHLDB1,TAGLN |
| ASPSCR1-TFE3 | fusion gene/product | Activated | ATP6V0C,CD59,CTSD,EPAS1,GRN,HLA-E,PACSIN2,PMP22,SOD2 |
| TEAD3 | transcription regulator | Activated | CALD1,COL3A1,IGFBP5,SAMD5,SH3D19,TAGLN |
| PDGFRB | kinase |  | ACTA2,COL1A2,MYH11 |
| PI3K (family) | group |  | ACTA2,CCNG2,CTSD,CXCL12,FTH1,HSPA5,MMP2,SOX4,TXNIP |
| RNASEL | enzyme |  | CTNND1,MT-ATP6,MT-CO2,PCDH9 |
| LARP1 | translation regulator | Inhibited | EEF1A1,EEF1G,EEF2,RPL13A,RPL17,RPL35,RPL36A,VIM |
| RETREG3 | other |  | COL1A1,COL1A2 |
| TSIX | other |  | COL1A1,COL1A2 |
| FGF12 | other |  | ACTA2,TAGLN |
| PNKP | kinase |  | MT-CO2,MT-CO3 |
| IGFBP4 | other |  | IGFBP3,IGFBP6 |
| PKM | kinase |  | GSR,MMP2,MT-ATP6,MT-CO1,MT-CO2,MT-ND3,NFE2L1 |
| UBA1 | enzyme |  | ANXA2,HSPA5,TAGLN,VIM |
| ITGA11 | other |  | ACTA2,COL1A1,DES,SPARC |
| AIF1 | other |  | ACTA2,COL1A1,COL3A1 |
| PPARG | ligand-dependent nuclear receptor |  | ACTA2,COL1A1,COL1A2,EPAS1,IGFBP3,IGFBP5,IGFBP6,TGFBR2 |
| VHL | transcription regulator |  | EPAS1,FTH1,LMNA,SOD2,VIM |
| EOMES | transcription regulator | Inhibited | ACTA2,ANXA1,COL3A1,H19,IGFBP3,PMP22 |
| HBEGF | growth factor | Activated | CCND2,GLUL,MMP2,MYH9,PIEZO1,VIM |
| HIF1A | transcription regulator | Activated | COL1A2,CXCL12,EPAS1,FHL1,FHL2,IGF1,IGFBP3,MIF,MMP2,MYH9 |
| ZC4H2 | other |  | MT-CO1,MT-CO3,MT-CYB |
| FZD8 | G-protein coupled receptor |  | ACTA2,COL1A1,VIM |
| PDGFRA | kinase |  | ACTA2,COL1A1,PDGFRA |
| ZAP70 | kinase |  | ANXA1,PLCG2,TSC22D3 |
| LGALS1 | other |  | ACTA2,CCND2,COL1A1,DISP1 |
| CEBPA | transcription regulator |  | A2M,ACTA2,ANXA1,C3,CCND2,H1-10,HLA-B,SOD2,TAGLN,VIM |
| NF-Y (complex) | complex |  | FTH1,HLA-DRA,HSPA5,TGFBR2 |
| TEAD1 | transcription regulator | Activated | CALD1,COL3A1,IGFBP5,SAMD5,SH3D19,TAGLN |
| VGLL3 | other |  | COL1A1,COL1A2,SOX4 |
| EGFR | kinase | Activated | ACTA2,HNRNPA2B1,HSPA5,IGF1,IGFBP3,MARCKS,MT-CO2,PPIA,VIM |
| MAX | transcription regulator |  | CCND2,CCNG2,DLEU1,RARB,TXNIP |
| RAD21 | transcription regulator |  | CCNG2,CXCL12,HLA-DRA,SOX4,VIM |
| IL13 | cytokine |  | ADAM28,C3,COL1A1,COL1A2,EPAS1,GSN,HOMER2,LIPA,MAF,PIEZO1 |
| SPDEF | transcription regulator | Inhibited | COL1A1,COL4A5,COL6A1,COL6A2,LAMB2,VIM |
| GLI1 | transcription regulator |  | AHNAK,ANXA1,CCNG2,CD59,COL1A1,EMP1,FHL1,IGFBP6,LMNA,MMP2 |
| TWIST1 | transcription regulator |  | ACTA2,C3,COL1A1,CXCL12,LMNA,MMP2,VIM |
| SMAD1 | transcription regulator |  | ACTA2,COL1A1,COL1A2 |
| MAOA | enzyme |  | ACTA2,IGFBP3,VIM |
| mir-21 (includes others) | microRNA |  | ACTA2,FAM3C,GAS5,RECK,TIMP3 |
| DLX1 | transcription regulator | Activated | FBLN1,FSTL1,LTBP3,PMP22,TPM2,VIM |
| MACROH2A1 | other |  | ANXA2,CCND2,FANK1,MAP2K6,SPARC,TJP2 |
| IFN BETA (family) | group |  | CD74,EEF1A1,HLA-B,MT-CO2,MT-ND2 |
| ADAR | enzyme |  | B2M,HLA-A,HLA-B |
| RAB4A | enzyme |  | MMP2,SPARC,VIM |
| INHBA | growth factor |  | ACTA2,CCND2,GRSF1,IGF1,MAF,PCDH9,SOX4,TAGLN |
| HISTONE H3 (family) | group |  | B2M,CCND2,CD9,HSPA5,IGFBP3,MAF,NDUFV1,NEAT1,RAB27B,RARB |
| USF1 | transcription regulator |  | B2M,CCNG2,IGFBP3,PFKL,TXNIP |
| BTG2 | transcription regulator |  | IGFBP4,IGFBP6,RARB,SOD2 |
| MYC | transcription regulator | Activated | AOPEP,CCND2,CCNG2,CLU,COL1A1,DLEU1,EEF1A1,GSR,H19,MGP |
| YAP1 | transcription regulator | Activated | ACTA2,COL1A1,CTSD,HLA-B,HLA-E,JAK1,MMP2,RAD50,ST7,TAGLN |
| E2F3 | transcription regulator |  | CD9,CTNND1,L3MBTL4,LAMA4,MAL2,NEAT1,TIMP3,TM9SF2 |
| RBM5 | other |  | ANXA1,ATP6V0C,IFITM3,RAB1A,UBA1 |
| MECP2 | transcription regulator |  | ANXA1,BSG,IGFBP3,RARB,VIM |
| FOXC1 | transcription regulator | Activated | B2M,CLU,HLA-B,HLA-C,IFITM3,IGFBP3,MID1,SAMHD1 |
| MRTFA | transcription regulator | Activated | ACTB,CXCL12,MYH9,MYL9,PDGFRA,SELENOP |
| PPARGC1A | transcription regulator | Activated | ATP5F1B,IGF1,MT-CO1,MT-CO2,MT-ND1,SOD2 |
| NFIC | transcription regulator | Activated | COL6A1,COL6A2,FAM3C,MMP2,NEGR1,SELENOP |
| SND1 | transcription regulator |  | ADIRF,EMP1,FLOT2,NR2F1,SOX4,TMEM109,VIM |
| MEG3 | other |  | ACTA2,COL1A1,VIM |
| PADI2 | enzyme |  | ACTA2,COL1A1,VIM |
| mir-148 (includes others) | microRNA |  | MMP2,TGFBR2,VIM |
| HFE | transmembrane receptor |  | B2M,CYBRD1,HSPA5 |
| RARA | ligand-dependent nuclear receptor |  | CD9,CTNND1,FGF12,HLA-B,HLA-C,PHLDB1,RARB,SAMHD1,SIPA1L1,TMT1A |
| CDK4/6 (family) | group |  | AHNAK,CALU,FLNA,LAMB2,MARCKS,TUBA1B |
| HSPA5 | enzyme |  | C3,CLU,COL1A2,FSTL1,MT-ATP6,MT-CO2 |
| SRF | transcription regulator |  | ACTA2,CALD1,FHL2,MYH11,MYH9,MYL9,TAGLN |
| STK26 | kinase |  | COL3A1,MMP2,MSN,VIM |
| NPM1 | transcription regulator | Activated | CALR,COL6A2,EGFL7,FTH1,HSPA5,IGFBP3,IGFBP5,MMP2,SOD2 |
| HDAC3 | transcription regulator |  | COL1A2,HLA-A,PFKL,RNASE4,VIM |
| AP2 (family) | group |  | MMP2,MT-CO2,PTGDS |
| YARS2 | enzyme |  | COX16,MT-CO2,MT-CYB |
| DAB2IP | other |  | CLU,EPAS1,VIM |
| SMARCC1 | transcription regulator |  | AEBP1,SMARCE1,SOD2 |
| TP73 | transcription regulator | Activated | ACTA2,CCNG2,COL1A1,FLOT2,IGFBP3,IGFBP4,IGFBP5,PMP22,UBE2V1,UBL3 |
| IGG (complex) | complex | Inhibited | B2M,CALR,CCND2,CD9,EMP1,HLA-E,HSPA5,IFITM3,MAF |
| FGFR4 | kinase |  | AHNAK,MTAP,SEPTIN11,TOMM20,VIM |
| MAFB | transcription regulator | Activated | CXCL12,IGF1,MARCKS,RNASE1,SELENOP |
| PRMT1 | enzyme | Activated | ACTA2,COL1A1,COL3A1,FTH1,RARB,VIM |
| NDRG3 | other |  | CALU,EEF1G,IGFBP3,IGFBP4,SOX4,TOMM20 |
| AGAP2-AS1 | other |  | MMP2,SOX4,VIM |
| TRPM2 | ion channel |  | ATG7,HSPA5,SOD2 |
| ITGAV | transmembrane receptor |  | COL1A1,COL1A2,MMP2 |
| TBXT | transcription regulator |  | COL1A1,MGP,SOX6 |
| TRIM21 | enzyme |  | B2M,HLA-A,TGFBR2 |
| INS | other |  | ACTA2,CCDC80,COL3A1,MT-ND4,PLCG2,RARB |
| YTHDF3 | other |  | PFKL,TGFBR2 |
| MILIP | other |  | MMP2,VIM |
| TMTC3 | enzyme |  | FAM3C,VIM |
| ZDHHC2 | enzyme |  | CD9,VIM |
| GLS | enzyme |  | ACTA2,COL1A1 |
| GLI3 | transcription regulator |  | ACTA2,COL1A1 |
| PLAU | peptidase |  | ACTA2,VIM |
| DLGAP1 | other |  | MMP2,VIM |
| RBM3 | other |  | ACTA2,TAGLN |
| AKT (family) | group |  | ACTA2,COL3A1,GSR,MMP2,MT-CO2,SOX4,VIM |
| SP3 | transcription regulator |  | COL1A1,COL1A2,HSPA5,IGFBP3,MMP2,SOD2,TGFBR2 |
| P38 MAPK (family) | group | Activated | CCNG2,COL3A1,CXCL12,FLNA,HSPA5,MIF,MMP2,MYH11,TJP2 |
| HYAL1 | enzyme |  | IGFBP4,PAPPA,PHLDB1,TAGLN,TGFBR2 |
| SAFB | other | Inhibited | B2M,CD74,HLA-C,HLA-DRA,HLA-E |
| USP8 | peptidase | Inhibited | B2M,HLA-A,HLA-B,HLA-C |
| ZEB1 | transcription regulator | Activated | COL1A1,COL1A2,MAL2,S100A10,VIM |
| HGF | growth factor |  | CCNG2,CNBP,FAM3C,IGF1,MMP2,SNRNP70,TRDN |
| ATG5 | other |  | GABARAP,MAP2K6,PDCD5,TOMM20,VIM |
| POLR3G | enzyme |  | LMNA,MMP2,VIM |
| SMARCD3 | transcription regulator |  | COL1A1,MGP,SOX6 |
| SIN3B | transcription regulator |  | CCNG2,COL1A2,TXNIP |
| BLM | enzyme |  | CHRDL1,COL1A2,COL3A1,PLPP3 |
| TGFBR2 | kinase |  | ACTA2,MMP2,PPIA,RBMS3,TGFBR2,TXNIP |
| GATA6 | transcription regulator |  | MYH9,PDGFRA,RARB,SOD2,SPARC |
| NR2F1-AS1 | other |  | NR2F1,VIM |
| S100A10 | other |  | ANXA2,S100A10 |
| ALDH2 | enzyme |  | ACTA2,COL1A1 |
| PIWIL1 | enzyme |  | FLNA,TPM2 |
| mir-455 (includes others) | microRNA |  | ACTA2,COL1A1 |
| RAI14 | transcription regulator |  | MMP2,VIM |
| ZBTB7B | transcription regulator |  | COL1A1,COL1A2 |
| ZKSCAN3 | transcription regulator |  | CCND2,PSAP |
| CYBA | enzyme |  | EPAS1,SOD2 |
| FOXA1 | transcription regulator | Inhibited | ANXA1,CCNG2,DSCAM,FSTL1,IGFBP3,MGP,MYH9,PALM2AKAP2,PFKL |
| ESR2 | ligand-dependent nuclear receptor |  | ANXA1,C3,CXCL12,DOCK10,FBLN1,HOMER2,IGFBP5,MAF,VIM |
| BMPR2 | kinase |  | CLIC4,MSN,RAB11B,YWHAH |
| HAND2 | transcription regulator |  | COL1A1,COL1A2,COL3A1,DES |
| ADGRE2 | other |  | COL6A1,FSTL1,GLUL,TGFBR2 |
| SNHG11 | other |  | BSG,CCND2,VIM |
| MTA1 | transcription regulator |  | CCND2,MT-CO2,SOX4 |
| HDAC (family) | group |  | CCNG2,IGFBP3,RARB,TGFBR2,TXNIP |
| PPARA | ligand-dependent nuclear receptor |  | C3,IGFBP3,IGFBP5,IGFBP6,MT-CO2 |
| NSUN6 | enzyme | Activated | FLNA,FSTL1,MARCKS,PMP22 |
| METTL3 | enzyme |  | ACTA2,CD9,DES,MICOS10-NBL1/NBL1,PDGFRA,VIM |
| SYVN1 | transporter | Activated | HLA-A,HLA-C,PCBP1,PLPP3,PODXL,PTPRJ,TLE5,VIM |
| TBX5 | transcription regulator |  | COL1A1,COL1A2,COL3A1,DES |
| let-7 (includes others) | microRNA |  | ACTA2,DDX18,VIM |
| KRAS | enzyme |  | COL1A2,CXCL12,EEF1A1,FTH1,GSN,JAK1,MT-CO1,MT-ND4,TNIK |
| DICER1 | enzyme |  | CANX,MAF,MT-ND5,SLC4A4,SPARC,VIM |
| NEUROG1 | transcription regulator |  | C3,CCND2,COL3A1,EPAS1,PAPPA |
| ERK1/2 (family) | group |  | B2M,C3,CALR,COL3A1,H19,HLA-A,HSPA5,MMP2 |
| MTO1 | enzyme |  | MMP2,VIM |
| ST6GALNAC1 | enzyme |  | B2M,VIM |
| NELFCD | other |  | BSG,DLGAP5 |
| C3AR1 | G-protein coupled receptor |  | C3,HSPA5 |
| PRKG1 | kinase |  | ACTA2,TAGLN |
| DUSP4 | phosphatase |  | MMP2,VIM |
| mir-541 | microRNA |  | MMP2,VIM |
| mir-665 | microRNA |  | TGFBR2,VIM |
| FHIT | enzyme |  | MMP2,VIM |
| TMPO | other |  | COL1A1,COL3A1 |
| RASAL2 | other |  | MMP2,VIM |
| THY1 | other |  | MMP2,VIM |
| IMMT | other | Inhibited | IGFBP3,IGFBP6,LAMB2,MIF |
| CTR9 | other |  | CXCL12,EPAS1,IGFBP4 |
| CARM1 | enzyme |  | CKB,FTH1,IGFBP4 |
| NGEF | other |  | CCND2,MYL9,PEG3,SERPING1,TAGLN,TMT1A |
| LDL (complex) | complex |  | ACTA2,C3,HSPA5,MT-CO1,MT-ND1 |
| NR4A1 | ligand-dependent nuclear receptor |  | ACTA2,COL1A1,COL1A2,TXNDC5 |
| CLOCK | transcription regulator |  | ARHGEF10,CADM2,CALD1,IGFBP5,RCN2,THUMPD1,UBL3 |
| DCLK1 | kinase |  | CTSD,JAK1,SOX4,VIM |
| SAMMSON | other | Inhibited | ATP5F1B,MRPS28,MT-ND1,TOMM20 |
| HNRNPA2B1 | other |  | FHL1,GSN,H19,MFHAS1,NR2F1,PAPPA,PITPNC1,WWOX |
| POU2F1 | transcription regulator |  | ATP6V0C,HLA-DRA,PTGDS |
| SP1 | transcription regulator |  | ATP6V0C,BSG,CD59,COL1A1,HSPA5,IGFBP3,LIPA,LTBP3,MMP2,SOD2 |
| UQCC3 | other |  | ATP5F1B,ATP5ME,ATP6V0C,COX10-DT,COX16,NDUFV1,PFKL,UQCR10 |
| FOXO1 | transcription regulator |  | CCNG2,COL1A1,DLGAP5,PRC1,SOD2,SOX4,TXNIP |
| AP1 (complex) | complex |  | IGFBP3,MMP2,MT-CO2,SPARC |
| IGFBP2 | other |  | COL21A1,MMP2,RBM4,RBMS3 |
| JNK (family) | group |  | ACTA2,ATG7,CXCL12,HSPA5,MMP2,TGFBR2 |
| GSTO1 | enzyme |  | CD9,CTSD,FUT8,MYH9,SOX4 |
| TP53 | transcription regulator |  | ACTA2,ACTB,ADGRB3,ANXA1,ATG7,CD59,CLU,COL1A1,COL3A1,CTSD |
| TSPYL5 | other |  | ASB3/GPR75-ASB3,AZIN1,FUT8 |
| NCOR2 | transcription regulator |  | C3,IGF1,RARB |
| KL | enzyme |  | HSPA5,IGF1,SOD2 |
| ID1 | transcription regulator |  | IGFBP3,MMP2,VIM |
| SDCBP | enzyme |  | MMP2,SOX4,VIM |
| EP300 | transcription regulator |  | ACTA2,CCNG2,HULC,IGF1,IGFBP6,PFKL,SOD2 |
| PDGF-BB (complex) | complex |  | CERT1,CNN1,COL1A1,COL1A2,COL3A1,LMNA |
| CG (complex) | complex | Activated | C1R,CNN1,CXCL12,EMP1,FGF12,HLA-E,IGFBP3,MAF,MMP2,PLPP3 |
| MYOC | other |  | ANXA1,CHRDL1,HSPA5,IGFBP5,RAB27B |
| PIK3CA | kinase |  | CCDC80,COL1A2,COL3A1,CXCL12,GSR,PLCG2,RCN2,VIM |
| TGF BETA (family) | group |  | BSCL2,COL1A1,COL1A2,EPAS1,JAK1,NPAS3 |
| UHRF2 | enzyme |  | ADIPOR2,CKB,PDCD5 |
| SAFB2 | other |  | B2M,HLA-C,HLA-E |
| STAT3 | transcription regulator | Activated | ACTA2,CCND2,COL1A1,COL1A2,EPAS1,H19,MMP2,MT-ATP6,MT-ND1,TAGLN |
| IL6 | cytokine |  | ACTA2,C3,CLU,H19,HSPA5,MMP2,SOD2,TGFBR2 |
| PTPRR | phosphatase | Inhibited | COL6A1,FLNA,MYH9,PFKL,PIEZO1,SLC2A4RG |
| SSTR2 | G-protein coupled receptor |  | CALD1,CCND2,PMP22,TMEM176B,TXNIP,VIM |
| BANF1 | other |  | HLA-A,HLA-E,IFITM3,JAK1,SCML1 |
| FOXO3 | transcription regulator |  | CCND2,CCNG2,SOD2,TXNIP,VIM |
| BSG | transporter |  | BSG,CXCL12,MMP2 |
| GLI2 | transcription regulator |  | ACTA2,CLU,COL1A1 |
| KDM3B | enzyme |  | APOD,C1R,DGKH,DLGAP5,PAPPA |
| SIX1 | transcription regulator | Inhibited | CFD,CLU,DES,SORBS1,TAGLN,TPM2 |
| SIRT6 | enzyme |  | COL1A1,COL3A1 |
| DEF6 | other |  | MMP2,VIM |
| HTR1A | G-protein coupled receptor |  | TGFBR2,VIM |
| OGG1 | enzyme |  | ACTA2,VIM |
| N4BP1 | enzyme |  | HLA-A,HLA-B |
| PTCD1 | other |  | MT-ATP6,MT-CO2 |
| TNFAIP6 | other |  | ACTA2,VIM |
| ELANE | peptidase |  | HSPA5,MMP2 |
| MMP12 | peptidase |  | MYH9,PSAP,PSMA6,YWHAH |
| GNA12 | enzyme | Activated | JAK1,MYH11,PDGFRA,VIM |
| SNAI1 | transcription regulator |  | COL1A1,COL1A2,SPARC,VIM |
| RNY3 | other |  | IFITM3,LY6E,SERPING1 |
| TNFSF12 | cytokine |  | ACTB,CTNND1,CXCL12 |
| PLA2R1 | transmembrane receptor |  | ATP5F1B,CTSD,SOD2 |
| JUN | transcription regulator |  | ACTA2,EPAS1,FTH1,HLA-B,IGFBP6,MMP2,MYH9,SOD2,VIM |
| SIRT1 | transcription regulator |  | CCND2,CCNG2,CD9,IGF1,MMP2 |
| RCN3 | other |  | CALR,CLU,HSPA5,RPL13A |
| XBP1 | transcription regulator |  | HLA-DRA,HSPA5,S100A6,SPARC |
| CEBPD | transcription regulator | Activated | COL1A1,COL1A2,COL6A1,COL6A2 |
| CUX1 | transcription regulator |  | FLNA,HLA-B,MARCKS,VIM |
| TGFB2-AS1 | other |  | FHL2,MGP,PLPP3,SELENOP,TAGLN |
| NFE2L2 | transcription regulator |  | FTH1,MMP2,MT-CO2,PRDX1,SOD2 |
| LIN28A | other |  | FUS,TAGLN,VIM |
| FAM117B | other |  | GSR,PRDX1,SOD2 |
| ZBED6 | transcription regulator |  | DLGAP5,SPTBN1,WWC1 |
| JAK1 | kinase |  | HLA-A,HLA-C,MMP2 |
| MAP2K1/2 (family) | group |  | C3,HSPA5,JAK1,VIM |
| EPAS1 | transcription regulator | Activated | CKB,CXCL12,FHL1,IGFBP3,NEAT1,PELP1 |
| RUNX2 | transcription regulator |  | ACTA2,MMP2,MYH11,TAGLN |
| NME1 | kinase |  | CTSD,GRSF1,HNRNPA2B1 |
| ATG7 | enzyme |  | ACTA2,COL1A1,COL1A2 |
| KDM1A | enzyme | Inhibited | COL1A1,COL1A2,RARB,SELENOP,SFRP2,SLC22A3,VIM |
| TREX1 | enzyme | Inhibited | B2M,GSN,PLPP3,S100A6,TXNIP |
