# Supplementary material for: Single‐nucleus and spatial transcriptomics of paediatric ovary: Molecular insights into the dysregulated signalling pathways underlying premature ovarian insufficiency in classic galactosemia
Source: Clin Transl Med. 2024 Oct 23;14(10):e70043. doi: 10.1002/ctm2.70043 (PMC11812122; doi:10.1002/ctm2.70043)
Supplement: Supplementary file 7 — Supporting information [file CTM2-14-e70043-s006.docx]

Supplementary Table S6

IPA Causal Network Analysis: Upstream regulator genes identified in the DEGs from ovarian follicle cluster [ST dataset: CG vs control]

| **Upstream Regulator** | **Molecule Type** | **Predicted Activation State** | **Target Genes in Dataset** |
| --- | --- | --- | --- |
| NTRK1 | kinase | Activated | AARS1,ADM2,AHNAK,ALDH1L2,ATF5,BEST1,C1R,CALR,CANX,CBLN4 |
| ERBB2 | kinase |  | AKT1,BCAM,BHLHE40,BMP7,CCN2,CCNB2,CD164,CDC14B,CDC25A,CDCA4 |
| ARID1A | transcription regulator | Inhibited | ACTA2,ANXA1,ARID5B,C3,C4A/C4B,C8orf44-SGK3/SGK3,CAV2,CAVIN2,CCNG2,CDH1 |
| ESR2 | ligand-dependent nuclear receptor |  | ACSL3,AKT1,ANXA1,ARID5B,BBC3,BID,BMP4,BMP5,C3,C8orf44-SGK3/SGK3 |
| NORAD | other |  | BHLHE40,CCN1,CCN2,COL5A1,DDIT3,GADD45B,HSPA5,ID3,IGFBP3,IL32 |
| TGFB1 | growth factor | Inhibited | ACAN,ACTA2,AKR1C1/AKR1C2,ARHGEF2,BGN,BHLHE40,BMI1,BMP7,CALR,CAV1 |
| SSTR2 | G-protein coupled receptor |  | ARID5B,BEST1,CCN1,CCN2,CDH1,CEBPB,COL6A3,DPYSL3,EIF1,FGF9 |
| NEUROG1 | transcription regulator |  | C1S,C3,CAVIN2,CD82,CEMIP,COL3A1,CYSLTR2,DSP,FGF9,FN1 |
| SBDS | other |  | AKNA,AKR1C1/AKR1C2,AKR1C3,CCN2,CRABP1,CRMP1,CRYM,DDIT3,DERL3,DNAJB1 |
| CTNNB1 | transcription regulator |  | ADGRG2,APOD,AURKA,BMP4,CDH1,CDKN1A,CNN2,COL3A1,CTNNA2,CTNNB1 |
| TP53 | transcription regulator |  | ABCD4,ACBD4,ACSL3,ACTA2,ACTB,ACTL6A,ADA,AKT1,ANXA1,APOBEC3C |
| AKT (family) | group |  | ACTA2,AMOT,BMF,BMI1,BTG2,CCN2,CDH1,CEBPB,COL3A1,CTNNB1 |
| FOXO1 | transcription regulator |  | CCN2,CCNB2,CCNG2,CDKN1A,CDKN1B,CDKN2C,COL1A1,COL2A1,CXCL10,DEPDC1 |
| ROR1 | kinase | Inhibited | CDH1,CDKN2C,EMP2,ETV5,FSTL1,GAS6,MYC,SGK1,SNAI2,STMN1 |
| COPS5 | transcription regulator |  | ADA,AMD1,BCAM,CD82,CDKN1B,CDKN2C,CENPH,CSE1L,DDIT4,DEPDC1 |
| FZD8 | G-protein coupled receptor |  | ACTA2,CCN2,CDH1,COL1A1,FN1,TJP1,VCAN,VIM |
| AKT1 | kinase | Inhibited | AKR1B10,AKR1C1/AKR1C2,AKT1,CDKN1B,CTNNB1,DIAPH2,FOSB,FOXO1,GSR,GSS |
| TCF (family) | group |  | ADGRG2,APOD,BMP4,CNN2,CTNNA2,DDX17,DPEP1,ECM1,EPHB3,F13A1 |
| MYC | transcription regulator |  | ACAN,AKT1,ALCAM,BBC3,BMI1,CABLES1,CAV1,CCNA1,CCNG2,CDC25A |
| NPM1 | transcription regulator |  | AKR1C1/AKR1C2,AKR1C3,CALR,CAMK1D,CCN1,CCN2,CEBPB,COL5A1,COL7A1,CTNNB1 |
| PDGF-BB (complex) | complex | Inhibited | BHLHE40,BMP6,CCL8,CCN1,CCN2,CEBPB,COL1A1,COL1A2,COL3A1,COL5A1 |
| EML4-ALK | fusion gene/product |  | CCNA1,CDC14B,CDH1,CDKN1A,CDKN1B,CDKN2C,COL3A1,COL5A2,CTNNB1,EGR1 |
| CDKN2AIP | transcription regulator |  | DDIT3,ERN1,GPX3,HMOX1,HSPA1A/HSPA1B,HSPA5,HSPA6,NUPR1 |
| TP63 | transcription regulator |  | ACTL6A,ADA,ADA2,AGO2,AKT1,BBC3,BHLHE40,BMP4,CCN1,CD82 |
| SMAD4 | transcription regulator |  | ARHGEF2,AURKA,BBC3,BMI1,CCN2,CCNG2,CDH1,CDK17,CDKN1A,CELF2 |
| mir-3622 (includes others) | microRNA |  | AIFM2,BBC3,CCNB2,CDKN1A,GADD45A,GADD45B,MYC,SESN1,TP53I3,ZMAT3 |
| GLI1 | transcription regulator |  | AHNAK,AKR1C1/AKR1C2,AKR1C3,AKT1,ANO1,ANXA1,ARHGAP29,ATP6V1C2,BBC3,BHLHE41 |
| HSPA5 | enzyme | Inhibited | C3,CDH1,COL1A2,CXCL10,CYCS,DDIT3,DNAJB9,EIF2A,ERN1,FHOD1 |
| ERBB3 | kinase |  | CDH1,CDKN1B,CDKN2C,EMP2,ETV5,FN1,FSTL1,GAS6,PHGDH,SGK1 |
| LACTB | peptidase | Activated | CCN2,CDH1,COL7A1,DPYSL3,FN1,GLIPR1,GREM1,LAMC2,PMEPA1,SERPINE2 |
| SMARCA4 | transcription regulator |  | ABLIM3,AHNAK,ALDOC,ANO1,ANTXR2,AP1S2,ATP2B4,CALB2,CCDC9B,CCN2 |
| GPER1 | G-protein coupled receptor |  | BHLHE40,CACNA1D,CCN1,CCN2,CYP19A1,DDIT3,DDIT4,EGR1,ERRFI1,FOS |
| ESR1 | ligand-dependent nuclear receptor |  | ABLIM1,ACTA2,AHNAK,BCAS3,BMP4,C3,CAV1,CAV2,CCNG2,CCPG1 |
| NSUN6 | enzyme |  | CAVIN2,CDKN2C,DAB2,EMP2,ETV5,FSCN1,FSTL1,GAS6,PHGDH,SGK1 |
| ESTROGEN RECEPTOR (family) | group |  | AGO2,ANXA1,ANXA9,BMP7,C3,CALB2,CAV1,CAV2,CCN2,CCNA1 |
| TMPRSS2-ERG | fusion gene/product |  | BBC3,BEX2,CALB2,CDH1,CEBPB,COL5A1,CREB3L1,DDIT3,DDIT4,DGKB |
| LRRFIP2 | other |  | ALCAM,C1R,C1S,C4BPB,CD74,CEMIP,COL4A5,COL5A2,FDCSP,FSTL1 |
| SREBF1 | transcription regulator | Activated | AARS1,ACADS,ARF4,BEST1,BHLHE40,BHLHE41,CDKN1A,CYCS,CYP51A1,ETHE1 |
| CEBPD | transcription regulator |  | AKR1C1/AKR1C2,CDKN1A,CDKN1B,COL1A1,COL1A2,COL5A1,COL6A3,CSF3R,CYP19A1,DDIT3 |
| PIK3CA | kinase |  | ARG2,BMP6,CDH1,CDKN1B,COL1A2,COL3A1,COL5A1,CXCL13,EPN3,GASK1B |
| PKNOX2 | transcription regulator |  | BBC3,BTG2,CDH1,CDKN1A,EGR1,GADD45A,MDM4,RPRM,SNAI2,VIM |
| SIN3B | transcription regulator |  | CCNG2,COL1A2,DDIT3,GADD45B,KLF6,MYC,SRD5A1,TXNIP,TYMS,YAP1 |
| INS | other |  | ACTA2,ANO1,CCN2,CDH1,CEMIP,COL3A1,CYP17A1,DDIT4,EGR1,GPAT4 |
| ATF6 | transcription regulator | Activated | AURKA,C8orf44-SGK3/SGK3,CRELD1,CRELD2,DDIT3,FOS,HERPUD1,HSP90B1,HSPA5,HYOU1 |
| RNF20 | enzyme |  | CASP9,FOS,FOSL2,HBA1/HBA2,MYC,NR4A2,RHOB |
| L2HGDH | enzyme | Inhibited | ADM2,DDIT4,MTHFD2,PHGDH,PSAT1,SLC7A11,SLC7A5,TRIB3 |
| OGDH | enzyme | Inhibited | ADM2,DDIT4,MTHFD2,PHGDH,PSAT1,SLC7A11,SLC7A5,TRIB3 |
| PI3K (family) | group |  | ACTA2,AKR1B10,AKR1C1/AKR1C2,CCNG2,CDH1,CDKN1B,CXCL10,DDIT3,DDIT4,FOS |
| ATF4 | transcription regulator | Activated | ATP1A2,CEBPB,CXCL2,DDIT3,DDIT4,FN1,HSPA5,IARS1,JUN,LAMP3 |
| SMAD3 | transcription regulator | Inhibited | ACTA2,ALPL,C3,CCN2,CCNG2,CDH1,CDKN1A,COL1A1,COL1A2,COL3A1 |
| FOXA1 | transcription regulator |  | ALDOC,ANXA1,ATP7A,BHLHE40,CCNG2,CD58,CDH1,CDKN1A,CXCL10,DDIT4 |
| BRD4 | kinase |  | ABLIM1,ACTA2,BBC3,BHLHE40,BTG2,BTN3A2,CDC25A,CDKN1A,CDKN1B,COL1A1 |
| RELA | transcription regulator | Activated | ATP6AP2,BACH2,BBC3,BEX2,BHLHE40,BTG2,CAV1,CCL19,CDKN1A,CEBPB |
| FOXA2 | transcription regulator |  | ACADS,ACSL6,ACTA2,ADA,AKR1C1/AKR1C2,ALDOC,APOA1,APOC4,ARID5B,BBC3 |
| BMP7 | growth factor |  | ACTA2,AKT1,CAMK2N1,CAV2,CDH1,COL5A2,CTNNB1,DSP,FN1,FZD7 |
| CDK19 | kinase |  | ACVR1B,ALDOC,BTG2,CD82,CDKN1A,CXCL2,DDIT4,FST,GADD45A,HMOX1 |
| SORL1 | transporter |  | BGN,C3,CAV1,CMKLR1,COL11A1,COL1A1,COL1A2,COL2A1,COL3A1,COL5A1 |
| SALL4 | transcription regulator |  | ACTB,BTG2,CAV2,CCN1,CCNB2,CCNB3,CD74,CDKN1B,CGN,CTNNB1 |
| SCD | enzyme |  | CEBPB,CTNNB1,CYP19A1,DDIT3,GADD45A,HERPUD1,HSPA5,XBP1 |
| AZGP1 | transporter |  | BMP4,CDH1,CDKN1B,CEBPB,DSP,EGR1,MYC,VIM |
| TGFB2 | growth factor | Inhibited | ACTA2,COL1A1,CXCL16,DDIT4,EEF2K,EGR1,FN1,FOS,ID3,ID4 |
| SMAD1 | transcription regulator |  | ACTA2,BTG2,CCN2,COL1A1,COL1A2,CXCL2,ID4 |
| RPS6KA3 | kinase |  | DDIT3,FOS,FSCN1,IL2,IRF4,MYC,TNFRSF10B |
| RNA POLYMERASE II (complex) | complex |  | ACTB,AURKA,CCNB2,CDH1,CDKN1A,COL1A2,CXCL2,ENO1,EXOSC8,FOS |
| PPARG | ligand-dependent nuclear receptor |  | ACTA2,APH1B,CAV1,CAV2,CDH1,CDKN1A,CERS3,COL1A1,COL1A2,CYP19A1 |
| YAP1 | transcription regulator | Inhibited | ACTA2,ARHGEF6,CAVIN2,CBL,CCN1,CCN2,CDH1,CDKN1A,CDKN1B,CDRT4 |
| FOXO3 | transcription regulator |  | BBC3,CCN2,CCNG2,CDH1,CDKN1A,CDKN1B,CXCL10,EGR1,FOXO1,FOXO4 |
| SP1 | transcription regulator | Activated | ATM,ATP2A3,BMP4,CAV1,CDH1,CDKN1A,CDKN1B,CEBPB,CHRNA5,COL1A1 |
| CCN5 | growth factor |  | CCN2,CDH1,DSP,FN1,GATA3,HIF1A,JUN,KLF4,LAMC2,SNAI2 |
| PGR | ligand-dependent nuclear receptor |  | AKR1C3,AMD1,BIRC2,CCL8,CCNB2,CDKN1A,CEBPB,CYP19A1,EDNRA,FN1 |
| TP73 | transcription regulator |  | ACTA2,ADA,BBC3,BHLHE40,BID,CCNG2,CDH1,CDKN1A,CDKN1B,COL1A1 |
| N-COR (family) | group |  | CDKN1A,CR2,CXCL10,ENO1,IGF1,IGFBP3,INPP4B,PGAM1,SNAI2,TNFSF11 |
| FIGLA | transcription regulator | Activated | ZP1,ZP2,ZP3,ZP4 |
| FA2H | enzyme |  | CCN1,CCN2,CDH1,VIM |
| NNMT | enzyme |  | ACTA2,COL1A1,PPARG,TCF21 |
| MYL9 | other |  | CCN1,CCN2,HIF1A,YAP1 |
| FOXF2 | transcription regulator |  | CDH1,FN1,FOXQ1,VIM |
| XBP1 | transcription regulator | Activated | ABCA12,APBB2,DDIT3,DNAJB9,DNAJC3,EDEM1,HSP90B1,HSPA5,KLF4,MYC |
| ERN1 | kinase |  | CCNA1,CDH1,DGAT2,DNAJB9,DNAJC10,EDEM1,LAD1,LGR5,LPCAT2,MCL1 |
| IL13 | cytokine |  | ADA,C3,CD1E,CLEC4A,COL1A1,COL1A2,DNASE2,DUSP10,F13A1,FLOT1 |
| HIF1A | transcription regulator |  | ACTL6A,ALDOC,AURKA,BACH2,BIRC2,CAV1,CCN1,CCN2,CDH1,CDKN1A |
| E2F3 | transcription regulator |  | AKNA,AKR1C1/AKR1C2,AKR1C3,BMI1,CAV2,CCNA1,CD9,CDC25A,DAG1,FST |
| RMRP | other |  | BBC3,BTG2,CDH1,CDKN1A,CTNNB1,KLF4,TGFBR1,VIM |
| TERC | other |  | CDH1,CDKN1A,CDKN1B,COL5A1,CTNNB1,PDPK1,PRKCA,TNXB |
| RNF2 | transcription regulator |  | AKR1B10,CDH1,CDKN1A,CDKN1B,CTNNB1,GREB1,TJP1,VIM |
| TRIB3 | kinase | Inhibited | DDIT3,DDIT4,GARS1,HERPUD1,HIF1A,LPL,MTHFD2,PCK2,PSAT1,TRIB3 |
| TNF | cytokine |  | ACTA2,ATP12A,BBC3,BHLHE40,BID,BIRC2,BTG2,BTG3,C1S,C3 |
| AR | ligand-dependent nuclear receptor |  | AKR1C3,AKT1,ARG2,CAV1,CAV2,CDH1,CDKN1A,CDT1,CEBPB,CTNNB1 |
| STIM1 | ion channel |  | ATF5,CAMK1D,CDKN1A,CDKN1B,CREB3L1,FGF9,FOXO1,KLF2,KLF4,MYC |
| HAVCR1 | other |  | BHLHE41,CALR,CCN1,CDKN1B,CEBPB,CEMIP,FSCN1,GLI2,HIF1A,HLF |
| TEAD4 | transcription regulator | Inhibited | ARID5B,CCN1,CCN2,CDH1,CDKN1A,CDKN1B,COL3A1,COL5A2,EDNRA,FJX1 |
| HAND2 | transcription regulator |  | ACE2,CCBE1,COL1A1,COL1A2,COL3A1,GJA1,GJA5,HSPB7,MYL7,NFATC2 |
| ELK1 | transcription regulator |  | CDKN1A,ITGB1,MCL1,MTHFD2,NUPR1,PRKCA,SAR1A,SLC7A11,SOCS3,THBS1 |
| FSH (complex) | complex | Activated | ACP5,ACTA2,ACTB,ACTR2,ADAMTS16,ALPL,BMPR1A,BTG2,CAPZA1,CASP9 |
| TEAD1 | transcription regulator |  | ARID5B,CCN1,CCN2,CDKN1A,CDKN1B,COL3A1,COL5A2,EDNRA,FJX1,MSLN |
| RUNX2 | transcription regulator |  | ACTA2,CDH1,CDHR5,CDKN1A,COL11A1,CYP19A1,FLRT2,FRA10AC1,LUM,MCL1 |
| OVOL2 | transcription regulator |  | ARHGAP31,ARHGEF2,ATM,CDKN1A,CDKN2C,EFNB2,ENO1,EPHA5,HAUS1,PGAM1 |
| CEBPB | transcription regulator |  | ACTA2,C3,CDKN1B,CEBPB,COL1A1,COL1A2,COL3A1,COL5A1,CSF3R,DDIT3 |
| OGA | enzyme |  | ABLIM1,ABLIM3,AIFM2,ALDOC,ARHGAP29,BRAF,CAV1,CCNG2,CD302,CDCA4 |
| CAB39L | kinase |  | ATP5MF,CDKN1A,CDKN1B,COX5B,COX8A,NDUFA1,NDUFA3,NDUFA4,NDUFB2,NDUFB3 |
| ATM | kinase |  | ACTB,ALKBH8,ATM,BIRC2,CCN2,CDKN1A,CDKN1B,FANCD2,FN1,GADD45A |
| WNT3A | cytokine |  | BMP4,CEMIP2,COLEC12,CTNNB1,DCLK1,FCMR,FOXQ1,HHIP,LGR5,SLC1A3 |
| ETV6-RUNX1 | fusion gene/product | Inhibited | ABLIM1,ACVR1B,AHI1,ANO1,ANTXR2,ARHGAP25,AURKA,BID,BLK,CD164 |
| COL18A1 | other |  | CCNDBP1,CDKN2C,DDIT4,EFNA1,EFNB2,FN1,FOS,HIF1A,ID1,ID3 |
| GATA4 | transcription regulator |  | ACE2,CCBE1,CEBPG,COL1A1,COL1A2,COL3A1,DSCC1,GJA1,GJA5,HSPB7 |
| KLF5 | transcription regulator |  | ANO1,BMI1,CDH1,CDKN1A,CDKN1B,CDT1,HHEX,HIF1A,HLF,ID3 |
| ERK (family) | group | Inhibited | ACTA2,AGER,BMF,CCN2,CDKN1A,CDKN1B,COL1A1,CXCL10,CXCL2,EGR1 |
| MAGI1 | enzyme |  | ABLIM1,ACP1,ACSL3,AMD1,ASF1B,BTG2,CDC25A,CDKN1A,CDKN1B,CSE1L |
| IL6 | cytokine |  | ACTA2,BBC3,C3,CD82,CDH1,CDKN1A,CDKN1B,CPS1,CXCL10,F13A1 |
| ECM1 | transporter |  | AKT1,BMI1,CDH1,ECM1,MYC,PTK2,SNAI2,VIM |
| SNHG20 | other |  | CCNA1,CDH1,CDKN1A,CTNNB1,MYC,VIM |
| MAOA | enzyme |  | ACTA2,CCN1,CCN2,IGFBP3,VIM,YAP1 |
| SHH | peptidase |  | BMP7,CDH1,CTNNB1,GLI2,HSD11B2,IHH,IL2 |
| S100A4 | other | Inhibited | ACTA2,CDH1,COL1A1,CTNNB1,NOTCH2,PTEN,VIM |
| PBRM1 | other |  | ABLIM3,ADAMTS9,ALDOC,CCNB2,CDKN1A,CXCL10,CXCL2,GCH1,JUN,LAMC2 |
| TNFSF10 | cytokine |  | AKT1,BIRC2,CSE1L,FOS,JUN,MCL1,NFKBIA,TNFRSF10B,XIAP |
| FOXP1 | transcription regulator | Inhibited | CDKN1A,CDKN1B,GINS1,IRF4,PRDM1,SFRP4,WNT1,XBP1 |
| CEBPA | transcription regulator | Activated | ACTA2,ANXA1,BTG2,C3,CDKN1A,CSF3R,DNMT1,FOXO1,FXR2,GCH1 |
| SIRT1 | transcription regulator |  | CCNG2,CD9,CDH1,CYP19A1,ENO1,FN1,HIF1A,HMOX1,IDH2,IGF1 |
| WBP2 | transcription regulator |  | BMF,BMP4,COL4A6,COL6A3,COLEC12,CTNNB1,CXCL2,DCLK1,EGR1,FCMR |
| ANXA2 | other |  | CDKN1A,GADD45A,IGFBP3,JUN,PTEN,SESN1,TNFRSF10B,TP53I3,ZMAT3 |
| ZEB2 | transcription regulator |  | CASP9,CDH1,CDKN1A,CTNNB1,HERC5,IL2,MYC,PTEN,VIM |
| EPCAM | other |  | CDH1,EGR1,FHL2,FOS,FOSL2,ID1,JUN,SNAI2,VIM |
| MALAT1 | other |  | CASP9,CCN2,CDH1,DNMT1,GINS1,HIF1A,MRE11,VIM,YAP1 |
| NR3C1 | ligand-dependent nuclear receptor |  | ACTA2,AKT1,ANXA1,BHLHE40,BIRC2,BMF,BRAF,CASP9,CAVIN2,CCN2 |
| BAG1 | other |  | ACTB,CDH1,CDKN1A,FOS,JUN,PTEN,PTK2,SNAI2,TJP1,VIM |
| MEK (family) | group |  | ABCE1,BMF,CDH1,CDKN1A,CDKN1B,CTNNB1,CXCL10,DNMT1,DSCC1,EGR1 |
| LRP6 | transmembrane receptor |  | ACTA2,CAV1,CCN2,CTNNB1,GATA3,TTYH1,VIM |
| SPP1 | cytokine |  | AURKA,CDH1,CTNNB1,DSP,FN1,FOXRED1,GATA6,GCDH,GSTZ1,JUN |
| CUX1 | transcription regulator |  | CDH1,CDKN1B,CXCL10,CXCL2,MYC,NDUFA1,PTK2,RHOBTB3,SATB2,SNAI2 |
| SERPINE1 | other |  | CCN2,CDH1,CDKN1A,FN1,TGFBR1,VIM |
| CLCA2 | ion channel | Activated | CASP9,CDH1,CDKN1A,CDKN1B,FN1,VIM |
| SPDEF | transcription regulator | Activated | CCN2,CDKN1A,COL1A1,COL4A5,COL4A6,COL5A1,COL5A2,COL6A3,HIF1A,LAMC1 |
| EIF5A2 | translation regulator |  | BHLHE40,CDH1,CEBPB,DDIT3,HERPUD1,HSPA5,HSPA6,RHOXF1,TXNIP,VIM |
| PDX1 | transcription regulator |  | ACE2,BBC3,CRELD2,DLK1,ERO1B,PHGDH,PPP1R1A,PRSS23,RHOBTB1,SLIT3 |
| HISTONE H3 (family) | group |  | BBC3,BTG3,CAV1,CD9,CDH1,CDKN1A,CDKN1B,COL4A6,CRIP2,CRISP1 |
| BRAF | kinase |  | BIRC2,BMF,CDKN1B,DNMT1,HIF1A,ITGB1,MCL1,PSME3,SLC5A5,XBP1 |
| TCR (complex) | complex |  | ABCD3,ABLIM1,APOD,ATP5PB,CTLA4,CXCL10,CXCL13,ENO1,FOS,GRIA3 |
| DNMT3A | enzyme |  | ADA2,CD9,CDH1,CXCL2,ECEL1,FABP5,GATA6,IRF5,JUN,MANF |
| IL17A | cytokine |  | ACAN,ACTA2,ATP12A,CCN2,CD83,CDH1,COL1A1,COL2A1,COL3A1,CTNNB1 |
| SRF | transcription regulator |  | ACTA2,ANO1,CCN2,CDKN1A,EGR1,FHL2,FOS,FOSB,GTF2H5,MCL1 |
| IRF4 | transcription regulator |  | CANX,CEMIP2,CXCL10,GPRIN3,ICA1,IL12A,IL2,INPP4B,IRF5,JAK2 |
| SATB1 | transcription regulator |  | ARF5,CDKN1A,CEMIP2,EBI3,GADD45B,GATA3,HSP90AA1,HSPA8,IL2,IL5 |
| SNAI1 | transcription regulator |  | CDH1,COL1A1,COL1A2,CYP19A1,FN1,ID1,L1CAM,PEBP1,PTEN,RAB25 |
| HSF1 | transcription regulator |  | BMP7,CBX3,CCDC117,CDH1,CKS2,CSRP2,CTNNB1,DEDD2,DNMT1,EFEMP1 |
| CD274 | transmembrane receptor | Inhibited | ACTA2,ATM,CDH1,CDKN1A,COL1A1,CTNNB1,FANCL,FN1,FOS,GJA1 |
| ETV5 | transcription regulator |  | ALCAM,CDH1,CTNNB1,FN1,ITGB1,KRT7,KRT80,NRCAM,SNAI2,TJP1 |
| SP3 | transcription regulator |  | CDKN1A,CDKN1B,COL1A1,COL1A2,COL2A1,CYP17A1,CYP51A1,EGR1,HSD11B2,HSD17B1 |
| ZC4H2 | other |  | COX6B1,COX8A,NDUFA1,NDUFA3,NDUFB3 |
| IDO1 | enzyme | Activated | SLC1A5,SLC3A2,SLC6A9,SLC7A11,SLC7A5 |
| MNT | transcription regulator |  | CCNG2,DDIT3,GADD45B,KLF6,TXNIP |
| MCAM | other |  | CDH1,FN1,ID1,SNAI2,VIM |
| MED16 | transcription regulator | Activated | CCN1,CCN2,CDH1,SLC5A5,TAGLN,VIM |
| CALR | transcription regulator |  | CDKN1A,COL1A2,DDIT3,FN1,HSPA5,IGFBP1 |
| EPHA2 | kinase | Inhibited | CXCL2,EGR1,FOS,FOSB,JUN,KLF6,SGK1,ZFP36 |
| TWIST2 | transcription regulator |  | BMI1,CDH1,CDKN1A,CTNNB1,FN1,PRKAA1,SNAI2,TFAM,VIM |
| FDX1 | transporter |  | DNAJB1,EGR1,FN1,HSPA1A/HSPA1B,HSPA5,ID1,JUN,SLC3A2,TUBA4A |
| ATF2 | transcription regulator |  | DDIT3,DMBT1,DUSP10,FN1,JUN,MCL1,PTEN,TOP2A,TRIB3 |
| PRKCA | kinase |  | CDKN1A,CXCL10,EGR1,FN1,HS2ST1,HSPA1A/HSPA1B,IGFBP3,JUN,PEG10,PTEN |
| SNAI2 | transcription regulator |  | BBC3,BMI1,CDH1,CDH15,CDKN1A,ITGB1,L1CAM,MYOM3,SNAI2,TNNC2 |
| CDKN1A | kinase | Activated | CDC25A,CDH1,CDKN1A,CDKN1B,CTSK,FN1,FST,HIF1A,IL18,IRF6 |
| KDM1A | enzyme |  | ASF1B,CDH1,CDKN1A,CENPU,CLCN2,COL1A1,COL1A2,CRYM,CTNNB1,CXCL10 |
| SIX2 | transcription regulator |  | ATP2A3,BBC3,CACNA1C,CACNA1D,CALM1 (includes others),CAMK1D,CCNB2,CDC25A,CDKN1A,CDKN1B |
| STAT5A/B (family) | group |  | CDKN1A,CDKN1B,CDKN2C,FCMR,MCL1,MYC,PCK1,PRDM1,RBP1,RFTN1 |
| BRCA1 | transcription regulator |  | ACTB,ATM,CCNB2,CDKN1A,CDKN1B,CTNNB1,CYP19A1,DDIT3,EGR1,FHIT |
| PKC (family) | group |  | BCL11B,CDKN1A,DDIT3,DNAJC3,ERN1,FOS,GADD45A,GADD45B,IL2,IRF6 |
| IGF1 | growth factor |  | BBC3,BHLHE40,BMP4,CDKN1A,CDKN1B,DDIT3,EFNB2,FN1,FOS,HIF1A |
| STX18 | transporter |  | ADA,ARHGAP29,BCAM,CCN2,CEP131,COL5A1,COL7A1,DPYSL3,FBLN1,FBN2 |
| NCOA3 | transcription regulator |  | CCN1,CCN2,CDC25A,CDKN1A,DDIT3,HMOX1,IGF1,IGFBP1,LAMP3,NCOA3 |
| CCN6 | growth factor |  | BMP4,CDH1,TNFSF11,VIM |
| ALDH2 | enzyme | Activated | ACTA2,CCN2,COL1A1,FN1 |
| ZBTB7B | transcription regulator |  | COL1A1,COL1A2,COL2A1,EOMES |
| RPS20 | other |  | BBC3,CDKN1A,MDM4,MYC |
| SF1 | transcription regulator |  | ACTA2,COL1A1,FN1,MYL6 |
| CYB561A3 | enzyme |  | ALDOC,ATF5,COTL1,HSPA1A/HSPA1B,HSPA6,MT1X,TFRC |
| PUM1 | other |  | AGO2,ANK2,CALM1 (includes others),CDKN1A,CDKN1B,FOXP1,PTPRS |
| USP36 | peptidase | Inhibited | CCN1,CCN2,FJX1,GADD45A,IGFBP3,MYC,YAP1 |
| HOXD13 | transcription regulator |  | COL5A2,FBN2,FN1,LAMC1,NT5E,SERPINE2,TGFBR1 |
| UBA1 | enzyme |  | BIRC2,HSP90B1,HSPA5,HSPA8,NEFL,TAGLN,VIM |
| mir-17 (includes others) | microRNA |  | AKT1,CCN2,CDH1,CDKN1A,COL11A1,FN1,VIM |
| SMARCD3 | transcription regulator |  | ACAN,ALCAM,COL1A1,COL2A1,KLF4,MGP,SOX6 |
| FOXO4 | transcription regulator |  | CCN2,CCNG2,CDKN1A,GADD45A,GADD45B,IGFBP1,SGK1 |
| EZH2 | transcription regulator |  | BMI1,BMP4,BMP7,C3,C4BPB,CABLES1,CACNA1E,CD82,CDH1,CDKN1A |
| HGF | growth factor |  | AKT1,ATM,BIRC2,CCNG2,CDH1,CDKN1A,CTSK,EGR1,FAM3C,FOS |
| ISLR | other |  | CCN1,CCN2,FOS,FSTL1,HSPA6,PRSS23,SLC7A11,SNAI2 |
| MOS | kinase |  | NDUFA1,NDUFB1,NDUFS6,TLE6,ZAR1L,ZP1,ZP2,ZP4 |
| NOTCH3 | transcription regulator | Activated | CDH1,CDKN1A,CTNNB1,ID1,MUC2,PTEN,SNAI2,VIM |
| HNRNPA2B1 | other |  | ADGRG2,CDH1,CEMIP,CPS1,CPVL,CRB1,DCLK1,DSE,FN1,FSCN1 |
| METTL3 | enzyme |  | ACTA2,ATP1A2,CASP9,CD9,CDH1,CDKN1A,CXCL10,CYCS,JUN,NDUFA4 |
| EGF | growth factor |  | CDH1,CEBPB,EGR1,FOS,FUT3,HIF1A,HSD17B1,LIN28A,MCL1,MUC2 |
| HDAC2 | transcription regulator |  | ACAN,AURKA,BBC3,CDH1,CDKN1A,COL11A1,COL1A2,COL2A1,DPT,IGF1 |
| CRTC2 | other |  | BACH2,CYP19A1,MYC,RHOB,SMAD6,SMARCA2 |
| PADI2 | enzyme | Inhibited | ACTA2,COL1A1,FN1,MYC,SNAI2,VIM |
| LINC00963 | other |  | CAB39L,CREB3L1,DDIT3,IL2RG,PCK2,TRIB3 |
| BAP1 | peptidase | Inhibited | CDKN1B,DDIT3,HIF1A,HSPA5,TNFRSF10B,XBP1 |
| LDB1 | transcription regulator |  | ALCAM,CARMIL1,IL18,KIT,KITLG,MKRN3,NKX3-1,SEMA6A,SLC15A1,SLC16A9 |
| NR0B1 | ligand-dependent nuclear receptor |  | CYP19A1,HSPA4L,KCNN2,NR0B1,STAR |
| AKT2 | kinase |  | CDH1,ITGB1,MTSS1,SLC5A5,VIM |
| SF3B4 | other | Inhibited | CDH1,CDKN1A,CDKN1B,TJP1,VIM |
| DKK1 | growth factor |  | BMP4,BMP6,CTNNB1,TNNT2,WNT5A |
| TNFAIP6 | other |  | ACTA2,CDH1,ITIH5,SNAI2,VIM |
| GPAT4 | enzyme | Activated | DDIT3,ERN1,HSP90B1,HSPA5,XBP1 |
| RPS15 | other |  | BBC3,CDKN1A,IGF2BP1,MDM4,PTEN |
| KRAS | enzyme |  | ARG2,AURKA,BMP6,CDKN1A,CDKN1B,COL1A2,COL5A1,CTNNB1,E2F5,EPN3 |
| LH (complex) | complex |  | ACP5,ACTA2,ACTB,ACTR2,ALPL,CAPZA1,CASP9,CYP19A1,DAB2,DUSP9 |
| mir-21 (includes others) | microRNA | Inhibited | ACTA2,ARF4,BTG2,C8orf44-SGK3/SGK3,CDKN1A,FAM3C,FBXO11,FN1,PTEN,SESN1 |
| SIN3A | transcription regulator |  | CCNG2,COL1A1,COL1A2,DDIT3,GADD45B,KLF6,MYC,TXNIP,YAP1 |
| PTGES | enzyme |  | ANXA1,CDH1,CDKN1A,CDKN1B,EGR1,MYC,NOP2,PPA1,VIM |
| ERK1/2 (family) | group |  | ALPL,APH1B,BIRC2,C3,CALR,CCL8,CDC42EP5,CDKN1B,CEBPB,COL3A1 |
| GROWTH HORMONE (family) | group |  | ARID5B,BMP4,CDH1,FOSL2,FZD5,FZD7,IGFBP3,LDLR,MYC,NFKBIA |
| ATF3 | transcription regulator |  | ADA,AURKA,CD82,CDC25A,CTNNB1,DDIT3,HSPA5,ID1,LDLR,PAICS |
| NOTCH1 | transcription regulator |  | ACTA2,CDH1,CDKN1A,CDKN1B,CEBPB,CTNNB1,EFNB2,HIF1A,IL18,MUC2 |
| GSTO1 | enzyme |  | CD9,FUT8,IFI6,LMO7,MTUS1,NID2,NPDC1,NR4A2,SCN9A,SHISAL1 |
| E2F1 | transcription regulator |  | BMI1,BMP4,CALM1 (includes others),CCNA1,CDC25A,CDCA4,CDH1,CDKN1A,CDKN1B,CDKN2C |
| IGG (complex) | complex |  | CALR,CD9,CDKN1A,CEBPB,CLEC5A,CXCL10,DDIT3,DSP,EFNA3,FABP5 |
| CD44 | other | Inhibited | ACTA2,ACTR6,AKT1,ALDOC,BIRC2,BMI1,CDH1,CDKN1A,DNMT1,ENO1 |
| LIN28A | other |  | CCN1,CCN2,CDC25A,CDH1,MYC,TAGLN,TIA1,VIM,YAP1 |
| HEIH | other | Inhibited | AKT1,FLNC,FOS,GADD45B,IGF1,JUN,KITLG,PDGFC,STMN1 |
| UPF2 | other |  | BMP5,CACNA1D,CAMK2N1,DSP,NEO1,NRP1,TSPAN12,TSPAN13,TSPAN8 |
| NRG1 | growth factor |  | CCN1,CDH1,CDKN1A,FN1,FOS,MYC,PTEN,SLC4A7,VIM |
| PTP4A3 | phosphatase | Inhibited | CDH1,FN1,HSP90AA1,PCBP1,PTEN,STMN1 |
| EZR | other |  | CDH1,CTNNB1,DDIT3,DDIT4,MYC,TRIB3 |
| DAB2IP | other |  | CDH1,CDKN1A,EGR1,IL32,MYC,VIM |
| ADORA2A | G-protein coupled receptor |  | COL3A1,CXCL10,FOS,HMOX1,NFKBIA,NR4A2 |
| NFKB (complex) | complex | Activated | BEX2,BIRC2,BMI1,C3,CASP9,CAV1,CCL8,CCNB2,CD83,CDKN1A |
| SOX9 | transcription regulator |  | CDKN1A,COL2A1,DMBT1,KLF4,MYC,PPARG,PRKCA,TSPAN8 |
| ADGRE2 | other | Inhibited | CDKN1A,COL6A3,FOS,FOXP1,FSTL1,GPNMB,HMOX1,JUN,MYC,WNT5A |
| PTPN3 | phosphatase |  | CDKN1A,COL1A1,FN1,MYC,TAGLN,TGFBR1,VDR |
| TCF4 | transcription regulator |  | ADAMTS9,AKT1,ARHGEF6,BHLHE40,BMP7,BMPR1A,CCT6A,CDH1,DPT,ECEL1 |
| SMAD2 | transcription regulator |  | ACTA2,CCNG2,CDH1,COL3A1,FN1,MIXL1,SERPINE2,SNAI2,TGFBR1,VIM |
| MTO1 | enzyme |  | CDH1,CTNNB1,MYC,VIM |
| SENP7 | peptidase |  | BIRC2,CACNA1C,CDH1,VIM |
| PRDM4 | transcription regulator | Activated | AKT1,CDKN1A,CDKN1B,PTEN |
| ST6GALNAC1 | enzyme |  | CDH1,CTNNB1,SNAI2,VIM |
| ATP6V1C2 | transporter | Inhibited | CDH1,CTNNB1,FN1,VIM |
| mir-541 | microRNA |  | CDH1,CTNNB1,MYC,VIM |
| FHIT | enzyme |  | BBC3,CDKN1A,SNAI2,VIM |
| RASAL2 | other |  | CDH1,TIAM1,VIM,YAP1 |
| SKI | transcription regulator |  | ACTA2,CDH1,CDKN1A,CDT1 |
| CCN3 | growth factor |  | BMI1,CTNNB1,GPNMB,ITGB1 |
| BLACAT1 | other | Inhibited | CDH1,CTNNB1,MYC,VIM |
| TARBP2 | other |  | MYC,NKX3-1,PROS1,THBS1 |
| RBCK1 | transcription regulator |  | CCN1,CCN2,MYC,YAP1 |
| IMMUNOGLOBULIN LAMBDA LIGHT CHAIN (family) | group |  | DDIT3,ERN1,HSPA5,XBP1 |
| INHBA | growth factor |  | ACTA2,CDC25A,CDH1,EFNB2,F13A1,GRIA3,GULP1,IGF1,IL12A,LEFTY1 |
| FALEC | other |  | BTG2,CDKN1A,ECM1,PTEN,TP53I3 |
| HOXA-AS2 | other |  | BRAF,CDKN1A,DDIT3,HOXA3,KLF2 |
| LOXL2 | enzyme |  | CDH1,DNAJC3,FN1,RAMP3,VIM |
| CCAT1 | other | Inhibited | CDH1,CDKN1A,CDKN1B,MYC,VIM |
| SH3KBP1 | other |  | CDH1,FN1,MYC,PRDM1,XBP1 |
| EIF2AK4 | kinase | Activated | DDIT3,DDIT4,EGR1,PCK2,PRDM1 |
| GSK3 (family) | group | Activated | BBC3,DDIT3,FOSB,JUN,LDLR,MYC,NR4A2,TNFRSF10B,XIAP |
| LAS1L | other |  | BTG2,CCNG2,CDC25A,CDKN1A,CEMIP,EPN3,INPP4B,LOXL4,SLC1A3,SPACA6 |
| TET2 | enzyme |  | AKR1C1/AKR1C2,AKR1C3,BMP4,BMP6,CCN2,CRB1,FOSB,GLI2,HOXA4,HSD17B1 |
| CD3 (complex) | complex |  | ATM,CBL,CD74,CDKN1B,CTLA4,EBI3,GATA3,HIF1A,IL2,IL2RG |
| QKI | other |  | CDH1,CDKN1A,CXCL10,FN1,FSCN1,HERC5,IFI44L,TAGLN,TNNT2,VIM |
| OTUB1 | enzyme |  | BIRC2,CCN1,CCN2,GADD45A,GLI2,GREB1,NT5E,NUAK2,PTPN14,YAP1 |
| RBPJ | transcription regulator |  | CDKN1A,CDKN1B,CR2,FGF11,FOS,JUN,MAPK14,MAPK6,MUC2,MYC |
| PTEN | phosphatase |  | AKT1,ARF4,BBC3,BMI1,CCNB2,CDC25A,CDKN1A,CDKN1B,ERRFI1,FGF9 |
| MET | kinase |  | AKT1,CDH1,CDKN1A,CDKN1B,FN1,HMOX1,ITGB1,MYC,NT5E,TGFA |
| CDK4/6 (family) | group |  | AFAP1,AHI1,AHNAK,ARHGEF2,EEF1B2,ENO1,ERRFI1,ITSN2,JPT1,LASP1 |
| RAD51 | enzyme |  | ATM,BTG3,CCNB2,CDKN1A,DDIT3,FANCD2,GADD45A,GADD45B,HMOX1,LGALS3 |
| BCL6 | transcription regulator |  | ACAN,ALCAM,CDKN1A,CHM,COL1A1,COL2A1,FCGRT,GADD45A,HERC5,KLF6 |
| PPP2CA | phosphatase |  | DNMT1,ICOS,IL2,IL2RG,NT5E,PTPRC,SELL,SOCS3 |
| GLI2 | transcription regulator |  | ACTA2,ANO1,CDKN1B,COL1A1,HHIP,HSD11B2,MYC,XIAP |
| NOX4 | enzyme |  | BMF,CDH1,CDKN1A,HIF1A,MCL1,TFAM,TGFA,TJP1 |
| UQCC3 | other |  | ALDOC,ATP2B4,ATP5MF,ATP5PB,ATP6AP2,ATP6V1C2,COX20,COX5B,COX6B1,COX7A2 |
| AGAP2-AS1 | other | Inhibited | CDH1,CTNNB1,FOXP2,MYC,SOX4,VIM |
| NUP62 | transporter |  | ADA,BBC3,CDKN1A,GLI2,IHH,PERP |
| PPP1R13L | transcription regulator |  | CDKN1A,CTNNB1,DSP,GJA1,ITGB1,PERP |
| SLIT2 | other | Activated | CDH1,CTNNB1,IL12A,NPY1R,NR0B1,SMARCA2 |
| CDK5 | kinase |  | CASP9,CDKN1A,IGFBP3,JUN,SPOCK1,XBP1 |
| LEP | growth factor |  | BID,CASP9,CDH1,CDKN1A,CDKN1B,COL6A3,ENO1,GADD45A,MTTP,MYC |
| MAPK9 | kinase |  | CAV1,CDKN1A,CDKN1B,GADD45B,GPAT4,LMO7,LPL,MYC,NR3C2,PDIA3 |
| FGFR4 | kinase |  | AHNAK,CDH1,CYRIB,ERCC5,FOS,FSCN1,GATA6,H3-3A/H3-3B,MYC,RBP1 |
| MFAP5 | other |  | CACNA2D3,CRELD2,ECM1,EFEMP1,GPX3,HSPE1,KIF4A,MRAS,NOTCH2,RAD51AP1 |
| SNCA | enzyme |  | ABLIM3,CACNA1D,CCBE1,CDH15,CDH9,DYNC1H1,EFHB,EFNA1,FABP7,GOLGA8K (includes others) |
| MAX | transcription regulator |  | CCNG2,CDC25A,DDIT3,GADD45A,GADD45B,GLYR1,KLF6,MYC,RBBP4,TXNIP |
| RAC1 | enzyme | Inhibited | CDH1,CTNNB1,FOXO1,GREB1,GSPT1,JUN,KLF4,MYC,SNAI2,WNT5A |
| NEDD9 | other |  | BHLHE40,CDH1,DDIT4,ERRFI1,FOS,KIT,PCK1,PLAC8,TXNIP,VIM |
| RAF (family) | group |  | CDKN1A,EGR1,ETV5,MYC,PTEN,SEMA3C,SEMA6A,SLC43A3,STK17A,STON1 |
| RBM5 | other |  | ANXA1,BMP5,BTG2,HSP90AA1,IL32,MCL1,MYO1B,NCOA3,RAB1A,THAP12 |
| MAP2K1 | kinase |  | CDKN1A,CDKN1B,DNMT1,FOS,HSPA5,JUN,LGALS3,RAB38,RAP2B,SNAI2 |
| STAT2 | transcription regulator |  | CAV1,CCL19,CXCL10,IFI6,IL12A,IRF5,ST13,WARS1 |
| PLA2R1 | transmembrane receptor |  | ACSL3,COX5B,MGST1,NDUFA5,PEBP1,SQOR,TFAM,UQCRFS1 |
| EP300 | transcription regulator |  | ACTA2,BBC3,CCNG2,CDC25A,CDKN1A,CYP19A1,DNMT1,FN1,FOS,FOXP1 |
| FOXM1 | transcription regulator |  | AURKA,CAV1,CCNA1,CCNB2,CDC25A,CDH1,CDKN1A,CDKN1B,CDKN2C,CKS2 |
| TRPC1 | ion channel |  | CDKN1A,CDKN1B,FOXO1,HIF1A,IGFBP1 |
| PTPN6 | phosphatase |  | CDKN1B,FN1,IL2,PCK1,VIM |
| VHL | transcription regulator |  | CDH1,CDKN1A,HIF1A,MAD2L1,NFE2,RUNX1,TFRC,TGFA,VIM |
| VDR | transcription regulator |  | ABCD4,CDKN1A,COL1A1,CTNNB1,CXCL10,IFI44L,IGFBP3,PDLIM2,THBD |
| NFKB1 | transcription regulator |  | AGO2,BHLHE40,CCL19,CCN1,CCN2,CD82,COL1A1,CR2,CXCL10,CXCL2 |
| SOX10 | transcription regulator |  | CDKN1A,FN1,IRF4,MYC,PTK2,RB1,SNAI2 |
| CIP2A | other |  | EBI3,ELAPOR1,GADD45A,GPNMB,LASP1,LDHB,LUM,LXN,PRG4,RHOD |
| IGF1R | transmembrane receptor |  | CDH1,CDKN1B,CEBPB,EGR1,HIF1A,IGF1,IL16,MYC,SNCG,STAR |
| CYTOR | other |  | CDH1,MAPK14,MYC,SLC25A1,SNAI2,VIM |
| CTBP1 | enzyme |  | BMP7,C8orf44-SGK3/SGK3,CCNG2,CDH1,CDKN1A,SMAD6 |
| SPARC | other |  | CDH1,FN1,JAK2,NOTCH2,SNAI2,TBX3 |
| CRP | other |  | AGER,CDH1,CTNNB1,EGR1,LPL,PRKCA |
| WDR73 | other | Activated | FN1,ITGA8,JUN,THBS1 |
| HERC3 | enzyme |  | CCN2,CDH1,VIM,YAP1 |
| ARHGAP4 | other |  | CDH1,CTNNB1,PTK2,TJP1 |
| HMMR | transmembrane receptor |  | BMI1,CDH1,FN1,VIM |
| TUBB4A | other | Inhibited | CDH1,CTNNB1,MYC,VIM |
| KRT17 | other | Activated | CDH1,SNAI2,TJP1,VIM |
| THBS4 | other |  | CDH1,COL2A1,COL3A1,VIM |
| MYB | transcription regulator |  | AURKA,BHLHE40,CCNA1,CDH1,FN1,GATA3,IL5,JUN,KLF4,MYC |
| SOX4 | transcription regulator |  | CEMIP2,CTNNB1,HDAC8,NRP1,PLXNA2,SEMA3C,SERPINE2,VIM |
| MAPK8 | kinase |  | CDKN1A,CDKN1B,CYP19A1,FOXO1,JUN,PTEN,RBP1,TBP |
| DHX9 | enzyme |  | CDKN1A,CXCL10,FN1,MAOA,MYC,PTPN13,SNAI2,YAP1 |
| HOXA5 | transcription regulator |  | CDH1,CDKN1A,CXCL13,IGFBP1,NFKBIA,SNAI2,TJP1,VIM |
| ELAVL1 | other |  | ARF5,ARG2,CALM1 (includes others),CCN1,CCN2,CD83,CDH1,CDKN1A,GATA3,GSS |
| SYVN1 | transporter |  | ACSL3,ASF1B,BHLHE40,CALM1 (includes others),CDA,DAB2,DAG1,GANAB,HERC5,ITGB1 |
| IFNG | cytokine | Activated | ACE2,ADA2,BBC3,BMF,C3,CDKN1A,CDKN1B,CEBPB,CTNNB1,CXCL10 |
| FTO | enzyme | Inhibited | CDH1,CDKN1A,CDKN1B,CTNNB1,FN1,HOXA5,JAK2,JUN,MYC,PDGFC |
| RYBP | transcription regulator |  | BMP4,BMP6,CCN2,FOSB,GLI2,ID1,JUN,RASSF6,WNT4,WNT5A |
| KLF4 | transcription regulator |  | ALCAM,BCL11B,CDH1,CDKN1A,CDKN1B,COL1A2,CTNNB1,LGR5,SLC4A7,SNAI2 |
| KDM3B | enzyme |  | ABCA12,ADRA2A,APOD,ARG2,ARL6IP1,C1R,CCN2,COLEC12,DEPDC1,EFNB2 |
| HOTAIR | other |  | ACTA2,CDH1,CDKN1A,JAM2,LAMC2,MYC,NFKBIA,PCK2,PPARG |
| THEM6 | other | Activated | CYP51A1,DDIT3,EDEM1,HERPUD1,HSP90B1,HSPA5,MSMO1,NIBAN1,XBP1 |
| KLF6 | transcription regulator |  | CDH1,CDKN1A,CDKN1B,CXCL2,HIF1A,MCL1,MSLN,MYC,NFKBIA,TFPI2 |
| TEAD2 | transcription regulator |  | ARID5B,CCN1,CCN2,COL3A1,COL5A2,EDNRA,FJX1,RHOBTB1,TAGLN,THBS1 |
| ERG | transcription regulator |  | ARHGAP31,ARHGEF2,ARHGEF6,BACE2,BMI1,CAMK1D,CCNB1IP1,CDH1,CEBPG,COLGALT1 |
| TGFBR2 | kinase |  | ACSL3,ACTA2,CFL1,CHRNA7,COL4A6,DSG2,GADD45B,JUN,PDPN,PFN1 |
| FOXC1 | transcription regulator |  | DSC1,ECM1,EGR1,FOS,FOSB,IFI35,IFI6,IFNAR1,IGFBP3,IRF6 |
| BCR (complex) | complex |  | ATP6V1C2,CBX3,CD27,CDKN1A,COX6B1,COX7A2,ENO1,FOS,FOSB,GRWD1 |
| RNF31 | enzyme |  | APOA1,AURKA,C8orf44-SGK3/SGK3,EGR3,GREB1,PDZK1,STAR |
| HLX | transcription regulator |  | EGR1,ELK1,FER1L6,JUN,MYC,PRDM1,RB1 |
| MAP3K12 | kinase |  | ACTB,JUN,STMN1,SYT10,TUBB,TUBB2A,TUBB4B |
| SPHK1 | kinase |  | BIRC2,BMP4,CASP9,CCN2,CDKN1A,HIF1A,IL2 |
| MRTFB | transcription regulator |  | ARID5B,CCN1,CCN2,COL3A1,COL5A2,EDNRA,EGR3,EPPK1,FJX1,ID1 |
| MYOC | other |  | ANXA1,DAB2,DDIT3,DDIT4,ESRP1,FN1,FSCN1,HHIP,HSPA5,INHBE |
| ZFAS1 | other |  | CDH1,KLF2,NKD2,SKA1,VIM |
| DANCR | other |  | CDH1,CTNNB1,RAB1A,SNAI2,VIM |
| ENO1 | enzyme |  | CDH1,ENO1,MYC,SNAI2,VIM |
| PTH | other |  | ALPL,DDIT3,FOS,HSPA5,IGF1 |
| RPA1 | other |  | FOCAD,NAV2,SYT16,TANC2,TPD52L1 |
| SMURF2 | enzyme |  | CDH1,FN1,HHIP,TGIF1,TRIM28 |
| RARA | ligand-dependent nuclear receptor |  | ABLIM1,APOA1,CCPG1,CD9,CDKN1A,CDKN1B,CENPU,CYP19A1,GREB1,H4C3 |
| ST6GAL1 | enzyme |  | ALCAM,CDH1,FN1,HIF1A,NRP1,SNAI2 |
| RRAS2 | enzyme |  | ALCAM,CAV1,CD74,COL6A3,CTLA4,DSC1,EGR1,EGR3,FSCN1,GPC3 |
| ARID2 | transcription regulator |  | BLK,COL1A1,DAB2,EFNB2,EXT1,FOS,KIRREL3,PTK2,SSC5D,THBS1 |
| HDAC6 | transcription regulator |  | CDH1,COL1A1,HIF1A,IGF1,JUN,MYC,TGFBR1,VIM |
| CDK8 | kinase |  | CDH1,CXCL2,FST,KLF2,MYC,SOX15,TBP,TBX3 |
| TRAP1 | enzyme |  | ALDH1L2,CHCHD3,COX5B,COX8A,GARS1,HSPE1,ISCU,MARS1,MTHFD2,SARS1 |
| BLM | enzyme |  | COL1A2,COL3A1,HMOX1,IFI44L,L1CAM,PLP1,RFTN1,TBX3,VAV3 |
| MMP1 | peptidase |  | CDH1,CDKN1A,IGF1,JUN,MAP2K1,MYC,NTRK3,TGFBR1,VIM |
| CDKN2A | transcription regulator |  | BBC3,BMI1,CCNA1,CDKN1A,CDKN2C,FOS,JUN,LGALS3,MCL1,MDM4 |
| SUZ12 | enzyme |  | CBX6,CCPG1,CDC25A,CDH1,IGFBP3,JAM2,LAMC2,LGALS3,MFN2,MYC |
| EFNA1 | other |  | ABCA12,BACH2,CCN2,CDKN1A,DSC1,ETV5,FABP5,GATA6,MYC,NT5E |
| A2M | transporter |  | EIF2A,ERN1,FOXO1,GADD45B,HSPA5,XBP1,XIAP |
| FN1 | other | Inhibited | CDH1,CTNNB1,ITGB1,KRT7,MUC2,MYC,SNAI2 |
| CAV1 | transmembrane receptor |  | CAV1,CDH1,CDKN1A,CTNNB1,CXCL10,SNAI2,VIM |
| NSD2 | transcription regulator |  | BACE2,BTG2,CDC25A,GADD45A,IGF1,ITGB1,JAM2,PRKCA,TOP2A |
| VCP | enzyme | Inhibited | CDKN1A,CDKN1B,CLNS1A,DDIT3,DIAPH2,DNPH1,HSPA5,IFT25,NUP35,RCC2 |
| IDH2 | enzyme |  | CDH1,EGR1,FN1,HIF1A,RAB25,VIM |
| INSIG1 | other |  | CYP51A1,DDIT3,ERN1,HSPA5,JUN,TRIB3 |
| PTTG1 | transcription regulator |  | CDH1,IGF1,MYC,SLC5A5,TGFA,VIM |
| PAK1 | kinase | Inhibited | CCN1,CCN2,CDKN1A,CTNNB1,FN1,MYC |
| ADAM32 | peptidase |  | KLF4,SNAI2 |
| IL2 | cytokine | Activated | ACVR1B,ATM,BCCIP,CACNA1E,CCNG2,CDC25A,CDKN1A,CDKN1B,CDKN2C,EEF1E1 |
| RUNX3 | transcription regulator | Activated | AKT1,BBC3,CDKN1A,RUNX1,TOX2 |
| TGFA | growth factor |  | AKT1,CEBPB,HSD17B1,MUC2,VIM |
| CLDN7 | other |  | ABLIM1,C1S,C3,CCN2,CCNA1,FOXQ1,IFI6,LGALS3,MT1X,PHGDH |
| mir-154 (includes others) | microRNA |  | ACTA2,CDH1,CDKN1B,KIT,MYC,PTEN |
| SPZ1 | transcription regulator | Inhibited | CDH1,FN1,HIF1A,SNAI2,VIM,WNT5A |
| AURKB | kinase |  | BBC3,CD82,CDKN1A,SORCS3,TP53I3,ZMAT3 |
| CDK4 | kinase |  | AURKA,C7,CDKN2C,CENPH,CTNNA2,DDIAS,ERI1,FZD7,GALNT11,GAS2L3 |
| TERT | enzyme | Activated | BMI1,CAV1,CDKN1A,CXCL10,GATA6,KLF4,LGR5,MYC,NFKBIA,RB1 |
| RNF187 | enzyme | Inhibited | ANKRD11,BHLHE40,BTG2,CDKN1A,HMOX1,ID1,JUN,KLF4,LDLR,NECTIN4 |
| NFE2L2 | transcription regulator |  | AKR1C1/AKR1C2,AKR1C3,CCN2,CDH1,DDIT3,HMOX1,NR0B1,PHGDH,PSAT1,SHMT2 |
| WWTR1 | transcription regulator |  | CCN1,CCN2,CDKN1A,DTYMK,FN1,THY1,TK1,TYMS,YAP1 |
| CSF1 | cytokine |  | ACP5,CDKN1A,CTSK,FN1,HSP90B1,HSPA5,IL12A,TNFRSF1B,XBP1 |
| TFRC | transporter |  | CDKN1A,F13A1,FOS,GADD45A,JUN,KIT,PPARG,SLC2A14,SULF2 |
| RRP15 | other |  | CTNNB1,FRZB,FZD7,PRKCA,WNT4,WNT5B,YBX2 |
| F7 | peptidase |  | CCN2,CXCL2,EGR1,FOS,GADD45A,MYC,ZFP36 |
| HOXA11-AS | other |  | CDH1,CDKN1A,CTNNB1,SNAI2 |
| SIRT6 | enzyme | Activated | CCN1,CCN2,COL1A1,COL3A1 |
| mir-3648 (includes others) | microRNA | Activated | CTNNB1,HIF1A,JUN,MYC |
| ASAH1 | enzyme |  | ACAN,CCNB2,CERS3,COL2A1,CYP17A1,NR0B1,NR4A2,STAR |
| ATG7 | enzyme | Activated | ACTA2,BMF,COL1A1,COL1A2,FN1,JUN,PTEN,SNAI2 |
| MAPK1 | kinase |  | AURKA,C1S,CCDC82,CDKN1A,COL7A1,CTNNA2,DSE,DXO,FLNC,FN1 |
| PAK4 | kinase |  | CCNA1,CDH1,SNAI2,TJP1,VIM |
| MXI1 | transcription regulator |  | IARS1,ID1,MTHFD2,MYC,SLC7A1 |
| CDK2 | kinase |  | BMI1,CDKN1A,FOS,MYC,TNFRSF10B |
| CBX3 | transcription regulator |  | CDC25A,CDH1,HSD11B2,MYC,PPL |
| MYH9 | enzyme | Inhibited | CDH1,CTNNB1,MYC,VIM,WNT5A |
| NANOG | transcription regulator |  | CCDC85A,CDC25A,CNOT7,COL3A1,EGR1,EOMES,FOS,FOSB,GATA6,HOPX |
| TWIST1 | transcription regulator |  | ACTA2,BMI1,C3,CDH1,CDKN1A,COL1A1,FN1,FOXO1,IFI6,IGFBP1 |
| DNMT3B | enzyme |  | BMI1,CDH1,CDKN1A,DNAJB1,EMILIN2,GLT8D2,IRF5,JAKMIP2,LACC1,LRRC34 |
| GDF2 | growth factor |  | ANOS1,CDH9,DAZL,GATA3,HTRA3,ID1,KIT,NRP1,SLC7A5,SMAD6 |
| MED1 | transcription regulator |  | AURKA,BHLHE40,CDKN1A,CYP17A1,DTYMK,DYSF,E2F5,EML1,HSD17B12,HYDIN |
| IKZF1 | transcription regulator |  | ANOS1,CAV1,CAV2,CEMIP2,FN1,FOS,GAS6,IFI6,IRF4,KLF4 |
| CAPN6 | peptidase |  | BIRC2,BMP4,BMP6,BMP7,BMPR1A,CCN2,FZD5,FZD7,GLI2,ID1 |
| PRDM5 | transcription regulator |  | CACNA1C,CD99,CEMIP,EDNRA,GADD45B,H4C16,LRRC37A3 (includes others),MYC,NAV2,NOTCH2 |
| DLX1 | transcription regulator |  | CDH1,DAB2,EML1,FBLN1,FSTL1,GJA1,HTRA1,KIT,TPM2,VIM |
| MACROH2A1 | other |  | BMI1,CDKN1A,CDKN2C,EGR1,FN1,GADD45A,HERC5,HIF3A,ID1,IL16 |
| FGFR3 | kinase | Inhibited | ANXA9,CDH1,CDKN1A,CR2,FN1,VIM |
| MAPK3 | kinase | Activated | CDKN1A,MYC,SLC4A7,TNFRSF10B |
| PCBP2 | other |  | CCN1,CCN2,CDKN1A,FANCD2 |
| KIF4A | other | Inhibited | CDH1,CDKN1A,ITGB1,VIM |
| ITGA11 | other | Inhibited | ACTA2,CCN2,COL1A1,FN1,THBS1 |
| NCR2 | transmembrane receptor |  | IL2,NR4A2,THBS1,TNFSF11,XCL1 |
| UHRF2 | enzyme |  | AKT1,CRIP2,CSE1L,FOXO1,JAG2,LY6H |
| NAMPT | cytokine |  | CXCL10,ID1,IL16,NAT1,NPY1R,PEG10 |
| SAFB2 | other |  | BBC3,BCCIP,FOS,JUN,L1CAM,PFDN4 |
| SYK | kinase |  | ATP8A1,CDKN1B,CXCL10,FCMR,FOXP1,FST,GADD45A,GATA3,IL2,KLF2 |
| EHF | transcription regulator |  | ACTA2,BMP4,CCBE1,CDKN1B,CEBPG,FOXN1,HMOX1,HOPX,KLK5,NOTCH2 |
| ODC1 | enzyme |  | BMP6,CDKN1A,CXCL16,FGF11,IL18,MANF,OSGIN2,WNT4 |
| NDRG3 | other |  | BOD1,DDX10,DPYSL3,EFNB2,EGR1,HSD17B12,IGFBP3,MEST,SEL1L,SLC45A4 |
| CD24 | other |  | CAV1,CDKN1A,DEPDC1,DIAPH2,ECM1,EMP3,HS2ST1,MTUS1,NAGK,NCOA3 |
| CDH1 | other |  | ACTB,BIRC2,CDH1,CTNNB1,FN1,GLI2,TJP1,VIM,XIAP |
| IRF3 | transcription regulator | Activated | CCL19,CXCL10,FN1,IFI44L,IFI6,IL12A,IRF5,SNAI2,VIM |
| HDAC (family) | group |  | CCNG2,CTNNB1,DDIT3,EGR1,EGR3,FOS,GADD45B,IGFBP3,JUN,KLF6 |
| IKZF3 | transcription regulator |  | ANOS1,CAV1,CAV2,CEMIP2,FN1,IFI6,KLF4,MYC,PROS1,SCN9A |
| ZBTB48 | transcription regulator |  | COL11A1,FOSB,GPNMB,TMEM100,TMEM63C,TUBA4A,VWA5A |
| SRC | kinase |  | CDH1,CTNNB1,HIF1A,ID1,MYC,PLAT,SLC4A7 |
| NDRG1 | kinase |  | CAV1,CD82,CDH1,CTNNB1,ERRFI1,SNAI2,VIM |
| FOXD2-AS1 | other |  | CDH1,CDKN1A,CTNNB1,PHGDH |
| ISG15 | other | Inhibited | COL1A1,COL1A2,COL5A2,COL7A1,GPX7,HERC5,IFI6,VCAN |
| NDUFA13 | enzyme | Activated | CDH1,CDKN1A,HIF1A,VIM |
| CLU | other |  | AKT1,ATP7A,CDH1,CDKN1A |
| STAU1 | transporter |  | CCNG2,EEF2K,KLF2,MYC |
| HNF1A-AS1 | other | Inhibited | CDH1,CTNNB1,ENO1,H2BC21,HMGB2 |
| SNHG11 | other |  | CTNNB1,ENO1,MYC,VIM |
| SKP2 | other |  | CDH1,CDKN1A,CDKN1B,VIM |
| PCSK9 | peptidase |  | CDH1,CDKN1A,LDLR,MIF |
| TEAD (family) | group | Inhibited | CAV2,CCN1,CCN2,FJX1,FN1,GADD45A |
| mir-221 (includes others) | microRNA |  | BMF,CDKN1B,PIK3R1,PTEN |
| PARP1 | enzyme |  | CDH1,CDKN1A,CTLA4,CXCL10,NAT1,PEG10,TNFRSF10B |
| MTOR | kinase | Inhibited | ATP6V1C2,CDH1,CDKN1A,CTNNB1,CXCL2,DDIT4,ENO1,HIF1A,HSPH1,MCL1 |
| MTDH | transcription regulator |  | CDH1,DDX17,PTEN,VIM |
| SPI1 | transcription regulator |  | ACTA2,CCNB2,CDKN1A,CXCL10,FCGRT,IFI44L,IFI6,LAMP3,MT1X,PRDM1 |
| BCL2L1 | other | Inhibited | ECM1,FN1,MCL1,THBS1,TNFRSF10B |
